# Charting the cognitive development of children using adult ‘polygenic g scores’

**DOI:** 10.64898/2025.12.19.695378

**Published:** 2025-12-23

**Authors:** Yujing Lin, Robert Plomin

**Affiliations:** 1 Social, Genetic and Developmental Psychiatry Centre, King’s College London

**Keywords:** Polygenic scores, cognitive ability, educational achievement, educational attainment, mental health

## Abstract

The most highly predictive polygenic scores in the behavioural sciences are for cognitive traits, especially general cognitive ability (g) and educational achievement. We combined polygenic scores derived from genome-wide association studies of adult g and educational attainment, conditioned on their prediction of adult g, to create adult ‘polygenic g scores’ which we used to chart the course of cognitive development of 10,000 white British children from infancy through early adulthood.

We integrated cross-sectional regression, latent growth curve, and cross-time common factor analysis to systematically characterise cognitive development. Polygenic g score showed minimal prediction in infancy, modest prediction in childhood, and substantial prediction by early adulthood, accounting for 12% of the variance. Higher polygenic g scores were associated with faster cognitive growth in latent growth models. Prediction was strongest for a cross-time common cognitive factor (15%), reflecting substantial stable genetic influences across development. We also examined the polygenic g score’s prediction of educational achievement, behaviour problems, and anthropometric outcomes and found similar developmental increases in prediction for educational achievement.

Together, our findings demonstrated that adult polygenic g scores can be a useful tool for charting the development of cognitive traits.

## Lede

Adult ‘polygenic g scores’ increasingly predict variance in cognitive ability across development from about 0.4% in infancy to 5% in childhood, 10% in adolescence, and 12% in early adulthood.

One of the most important outcomes of the DNA revolution for research on intelligence and cognitive abilities is the polygenic score (Plomin, 2019). Genome-wide association (GWA) studies associate DNA differences, typically common genetic variants called single nucleotide polymorphisms (SNPs), with cognitive abilities. Many such genetic variants contribute to the substantial heritability of cognitive abilities, and the largest effect sizes are minuscule, accounting for less than .05% of the phenotypic variance (Plomin & von Stumm, 2018; Visscher et al., 2021). The small effect sizes make it difficult to trace pathways from genes to brain to behaviour. In contrast, polygenic scores aggregate thousands of these small but additive effects, translating GWA discoveries into a genetic predictor of individual differences among unrelated individuals in the population.

Despite the availability of many polygenic scores for cognitive outcomes to date, two scores consistently drive the strongest predictions (Procopio et al., 2025). The first is a polygenic score for intelligence. The largest GWA analysis of adult intelligence (N = ~270,000) produced what has been called the IQ3 polygenic score, which predicts about 5% of the variance of adult intelligence in independent samples (Savage et al., 2018). GWA analyses require large sample sizes to detect the small effects of DNA variants. Conducting a GWA study of intelligence requires not only obtaining and genotyping DNA but also testing for intelligence, which is difficult with such large samples. For this reason, years of schooling (educational attainment) has been used as a proxy for intelligence (Rietveld et al., 2014). Educational attainment correlates strongly with intelligence, about 0.50 phenotypically (Deary, 2012) and 0.75 genetically (Hill et al., 2019). It is assessed with a single self-reported item about the highest level of education and, crucially, is routinely collected in most GWA studies as a demographic descriptor, making it possible to assemble huge samples for meta-analysis. The largest GWA analysis of educational attainment had a sample size of three million that yielded a polygenic score (EA4) predicting up to 9% of the variance of intelligence, with the strongest prediction observed for verbal ability (Okbay et al., 2022). EA4 predicts more of the variance of intelligence than IQ3, even though educational attainment is a coarse proxy for intelligence, because the EA4 sample size is more than ten times greater than the IQ3 sample size. Here, we combined IQ3 and EA4 weighted by their prediction of adult g and refer to this composite as an adult ‘polygenic g score’.

Because inherited DNA differences are fixed at conception, polygenic scores remain constant during development, which is unique in developmental research: Adult polygenic g score can predict adult g from birth just as well as in adulthood. If we turn this around, we can use the adult polygenic g score as a tool to chart the emergence, growth, and changes of cognitive abilities across development and to pinpoint the earliest cognitive traits that are genetically linked to adult cognitive abilities. To achieve this, we need longitudinal cognitive data that follow children from infancy to adulthood.

Such longitudinal research takes three decades, and as a result, few data exist. Meta-analytic evidence indicates that while cognitive ability tends to fluctuate in infancy, its stability increases rapidly across early childhood, becoming highly stable by adolescence (Breit et al., 2024). Although these longitudinal phenotypic correlations represent a ceiling for predicting adult g from infant measures, adult polygenic g scores can be used as a sharper scalpel for dissecting facets of early cognitive development linked *genetically* to adult g.

A recent study leveraging adult-derived PGS found that, similar to phenotypic correlations, genetic prediction follows a trajectory of increasing strength across development (Gustavson et al., 2025). The highest prediction reported was for EA4 PGS, explaining 18% of the variance in cognitive ability by age 16, which is twice as much as the usual result of 9% for adult samples (Savage et al., 2018). Twin analysis showed a similar pattern of increasing heritability. The IQ3 PGS also showed a parallel pattern but with lower predictive strength. Furthermore, the authors used confirmatory factor analysis for polygenic prediction and Cholesky decomposition for twin analysis to reveal the source of stability over time. They found that the increased prediction is attributable to the amplification of stable genetic effects shared across ages, rather than the emergence of new, age-specific genetic factors.

Our study builds on these findings using data from the Twins Early Development Study (TEDS; Lockhart et al., 2023), a population-based cohort with over 10,000 British white children. TEDS offers significantly greater power with a nearly tenfold increase in sample size compared to the Gustavson et al. (2025) report. TEDS children were assessed using diverse measures of cognitive development in infancy and early childhood (2, 3, and 4 years), middle childhood (7 and 9 years), adolescence (12, 14 and 16 years), and adulthood (26 years), providing repeated measures of g, verbal ability, and nonverbal ability.

We aim to replicate prior cross-sectional and common factor findings while addressing several developmental aspects that were not covered in previous work. First, we create a polygenic g score as a composite predictor by weighting EA4 and IQ3 based on their joint prediction of adult g (Maier et al., 2018). Second, we include latent growth curve modelling to chart developmental trajectories as predicted by the polygenic g score. Third, we look at the tails of the polygenic distribution to investigate if cognitive development diverges for the highest and lowest scorers. Finally, educational achievement, behaviour problems, and anthropometric outcomes were also assessed longitudinally, enabling a comprehensive examination of polygenic g score prediction across trait domains.

In our preregistration (https://osf.io/vhuyj/overview), we specified five hypotheses: 1) the polygenic g score significantly predicts most phenotypic outcomes, with educational achievement yielding the strongest correlation, 2) prediction for cognitive abilities increases from infancy to early adulthood; 3) the polygenic g score is also predictive of behaviour problems and anthropometric outcomes; 4) prediction is linear across the distribution, indicating that high and low extremes are quantitatively, not qualitatively, different from the rest of the distribution; and 5) there are no significant sex differences in polygenic g prediction.

## Methods

### Sample

We leveraged data from the Twins Early Development Study (TEDS), a longitudinal cohort of 13,759 families with twins born between 1994 and 1996 in England and Wales (Lockhart et al., 2023b). Phenotypic data were collected across multiple waves, including assessments at approximately ages 2, 3, 4, 7, 8, 9, 10, 12, 14, 16, 18, 21, 25, and 26 years. Genotypic data were available for 10,346 participants. Ethical approval for TEDS was obtained from King’s College London Research Ethics Committee (References: PNM/09/10–104 and HR/DP-20/2122060), and informed consent was obtained prior to each wave of data collection.

For cross-sectional analyses, all participants with available DNA data and at least one phenotypic measure were included. To calculate a total score for a given measure, participants were required to have completed at least half of the scale. For measures composed of multiple subscales, the same rule applied to each subscale, and a participant was included only if at least half of the subscales were available.

For longitudinal analyses, we included participants with data available for at least two ages (N = ~4500 to ~8000).

### Measures

#### Phenotypic Measures

The present study focuses on outcomes selected for their consistent collection across the ages. From ages 2 to 26, we assessed cognitive abilities, educational achievement, behaviour problems, and anthropometric measures consistently using age-appropriate measures. Additional measures specific to one age or one developmental stage are provided in Appendix A. Complete documentation of all measures across ages is available in the TEDS data dictionary (https://datadictionary.teds.ac.uk/home.htm).

##### Early Cognitive Abilities (Ages 2–4).

Phenotypic measures at early ages were collected via booklets sent to families. At ages 2 and 3, booklets were sent only to families of twins born in 1994 and 1995, as twins born in 1996 were not age-appropriate for the tests. At age 4, booklets were sent to all families.

Verbal ability at ages 2 to 4 included vocabulary (what children can say) and grammar (how children use words). Vocabulary was assessed via parent-reported checklists. At age 2, parents completed a 100-word checklist adapted from the MacArthur Communicative Development Index (MCDI; Fenson et al., 1993, 2000). At age 3, the checklist included 45 MCDI words and 55 new words from a literature review and pilot testing with an additional two questions about whether the child was talking and combining words. At age 4, 48 words selected from the literature review and pilot testing were used. Grammar was also measured using questions derived from the MCDI. At ages 2 and 3, parents completed the 6-item word use and 12-item sentence complexity scales. At age 3, the word use scale was expanded to 12 items. At age 4, a single 6-point global rating scale assessed language complexity from ‘not yet talking’ to ‘talking in long and complicated sentences.’ The verbal ability composite for the MCDI was calculated as the standardised mean of vocabulary and grammar scores. More detailed descriptions of the TEDS verbal measures between ages 2 to 4 are available in the TEDS data dictionary and previous TEDS publications (Dionne et al., 2003; Hayiou-Thomas et al., 2012).

Nonverbal ability was assessed using the Parent Report of Children’s Abilities (PARCA; Saudino et al., 1998), including parent-administered tasks and parent-report questionnaires. At age 2, parent-administered tasks included matching (8 items), brick building (4 items), folding (1 item), and copying (7 items) from the Bayley Scales of Infant Development (Bayley, 1993) and a design drawing task (4 items) adapted from the McCarthy Scales (McCarthy, 1972). The parent-report component assessed conceptual knowledge (26 items). At age 3, parent-administered tasks included odd-one-out (16 items), design drawing (6 items), and matching (16 items), with conceptual knowledge assessed via 24 items. At age 4, parent-administered tasks comprised the age 3 odd-one-out and design drawing tasks, plus draw-a-man (1 item) and puzzles (12 items). Conceptual knowledge was assessed via 12 items. The nonverbal ability composite was calculated as the standardised mean of parent-administered and parent-report PARCA scores, following the TEDS data dictionary and established practice in previous TEDS publications (Asbury et al., 2005; Oliver et al., 2004; Petrill et al., 2001; Saudino et al., 1998).

General cognitive ability at each age was calculated as the standardised mean of verbal and nonverbal composites.

##### Cognitive Abilities at Later Ages (Ages 7–25).

Cognitive ability measures at ages 7, 9, 10, 12, 16, and 25 have been described in detail in a previous TEDS publication and are only briefly summarised here (see Lin et al., 2025 supplementary materials).

At age 7, cognitive assessments were conducted via telephone interviews. Verbal ability was measured using the Wechsler Intelligence Scale for Children (WISC-III) Similarity and Vocabulary tests (Wechsler, 1992). Nonverbal ability was assessed using the Conceptual Grouping Test and WISC Picture Completion Test (McCarthy, 1972).

At ages 9 and 10, verbal ability was assessed using WISC-III as a Process Instrument (WISC-III-PI) Vocabulary and General Knowledge tests (Kaplan et al., 1999). Nonverbal ability was measured using the Cognitive Abilities Test 3 (CAT3) figure classification and figure analogy tests at age 9 and WISC-III-UK Picture Completion and Raven’s tests at age 10 (Raven et al., 1996; Smith et al., 2001). Assessments were administered via mailed booklets at age 9 and online at age 10 and all subsequent ages.

At age 12, verbal ability comprised language tests (syntax, semantics, pragmatics) and reading tests (comprehension and fluency) (GOAL plc (2002), n.d.; Hammill et al., 1994; Markwardt, 1997; Torgesen et al., 1999; Wiig et al., 1989; Woodcock et al., 2001). Nonverbal ability was assessed using mathematical ability tests from the National Foundation for Education Research (Smith et al., 2001). General cognitive ability was independently assessed using WISC-III-PI Vocabulary, General Knowledge, Picture Completion (Wechsler, 1992), and Raven’s Pattern test (Raven et al., 1996).

At age 14, verbal ability was assessed using a 27-item WISC-III-PI vocabulary multiple-choice test (Kaplan et al., 1999). Nonverbal ability was measured using the 30-item Raven’s Standard Progressive Matrices (Raven et al., 1996).

At age 16, verbal and nonverbal abilities were assessed using the Mill Hill Vocabulary test (Raven et al., 1998) and Raven’s Standard and Advanced Progressive Matrices (Raven et al., 1996).

Between ages 7 and 16, except for age 12, verbal and nonverbal composites were calculated as standardised means of their respective component tests, and general cognitive ability was calculated as the standardised mean of the verbal and nonverbal composites.

Age 18, assessment only included two spatial ability online measures developed by TEDS researchers (Malanchini et al., 2020; Rimfeld et al., 2017): a bricks test and a navigation study. The Bricks test measured spatial ability through mental rotation and visualisation using both 2D and 3D stimuli across six subtests (9 items each): 2D rotation, 2D rotation and visualisation, 2D visualisation, 3D rotation, 3D rotation and visualisation, and 3D visualisation. Both individual subtest scores and the overall Bricks total score (mean of all six subtests) were included in the present study. The Navigation test included 30 tasks across six types (5 items each): orientation-direction, orientation-landmarks, map reading without memory, map reading with memory, perspective, and scanning. Each task generated accuracy, speed, and total scores; only the overall total score (mean of the six task types) was used in analyses.

At age 25, cognitive abilities were assessed using Pathfinder, a gamified web-based measure developed by TEDS researchers (Malanchini et al., 2021). Verbal ability (20 items) included the Mill Hill vocabulary, missing letter, and verbal reasoning tests. Nonverbal ability (20 items) included Raven’s standard progressive matrices and three visual puzzle tests on analogies, grouping, and logical sequences. Unlike earlier ages, cognitive ability scores at age 25 were not standardised; general cognitive ability scores ranged from 0–40, while verbal and nonverbal scores each ranged from 0–20.

##### Educational Achievement.

Educational outcomes were examined using educational achievement from primary school to university. Educational outcomes at ages 7, 9, 10, 12, 16, 18, 21, and 26 have been described previously (Lin et al., 2025). The present study extended these general outcomes by including subject-specific grades up to age 18 and adding assessment at age 14.

At ages 7, 9, 10, and 12, teachers rated achievement in English and mathematics (starting at age 7) and science (starting at age 9) based on the National Curriculum Levels (https://www.gov.uk/national-curriculum/overview). The ratings ranged from 0–4 at age 7 and 0–9 at later ages.

At age 14, parents reported grades in English, mathematics, and science and the grades were translated to the 0–9 National Curriculum Levels.

At age 16, General Certificate of Secondary Education (GCSE) is a national-level exam taken at the end of compulsory education. GCSE exam grades were obtained for core subjects (English, mathematics, and science), humanities, and languages. The grades ranged from 4 (G) to 11 (A*).

At age 18, A-level and AS-level qualifications were assessed across English, mathematics, science, technology, humanities, languages, and vocational subjects. A-levels are two-year qualifications completed after compulsory education and required for university entry. AS-levels represent completion of the first year only. When A-level grades were unavailable, AS-level grades were used. Grades ranged from 1 (E) to 6 (A*).

At age 21, university degree classification was self-reported on a scale from 1 (lowest pass) to 5 (first-class honours).

At age 26, most twins have completed their education. Therefore, educational attainment (i.e., years of schooling) was used to measure educational outcomes. For twins missing age 26 data, age 21 educational attainment was used (correlation between ages: r = 0.86).

##### Behaviour Problems.

Behaviour problems were primarily assessed using the Strengths and Difficulties Questionnaire (SDQ) (Goodman, 1997), which was administered consistently across ages from 2 to 26 and across multiple informants. The SDQ yields five subscales: conduct problems, emotional problems, hyperactivity, peer problems, and prosocial behaviour. The first four problem subscales were summed to create a total problems score at each age.

Between the ages 2 to 4, the Preschool Behaviour Questionnaire (Behar scales) was used to measure parent-reported behaviour problems (Behar, 1977), with items converted to SDQ-comparable components by TEDS researchers (https://datadictionary.teds.ac.uk/pdfs/4yr/234yr_behaviour_items.pdf). From age 7 onward, the standard 25-item SDQ was administered with multiple informants: parent reports at ages 7, 9, 12, 16, and 21 (emotional and peer problem subscales were not available from parents at age 16); teacher reports at ages 7, 9, and 12; and self-reports at ages 12, 16, 21, and 26.

We also included measures of anxiety and ADHD symptoms collected at multiple ages. Additional behaviour problems assessed at one or two ages were examined as outcomes; these results are presented in the Appendices.

##### Socioeconomic Status (SES).

Family SES was assessed at birth and at ages 7, 16, and 21. Each SES composite was standardised as z-scores and calculated from parental employment status (coded according to the UK Standard Occupational Classification or SOC, https://www.ons.gov.uk/methodology/classificationsandstandards/standardoccupationalclassificationsoc/), parental highest educational qualifications, and household income.

#### Genetic Measures

Genotyping for the TEDS participants was conducted on one of two platforms: the Affymetrix Genome-Wide Human SNP Array 6.0 and the Illumina HumanOmniExpressExome-8v1.2. DNA was obtained from either buccal cheek swabs or saliva samples collected over several waves.

Following quality control, genotypes from both platforms were separately phased using EAGLE2 and then imputed to the Haplotype Reference Consortium (release 1.1) (Durbin, 2014; Loh et al., 2016; McCarthy, 2016). After imputation, harmonisation, and merging of the two datasets, a final set of 7,363,646 SNPs for 10,346 twins remained for analysis. More details of the genotyping and imputation processes are described in previous TEDS publications (Lin et al., 2025; Selzam et al., 2018).

We constructed polygenic scores using LDpred2-auto, a Bayesian method that adjusts GWAS summary statistics for linkage disequilibrium (LD) using the HapMap3+ reference panel (Privé et al., 2021). Approximately 1.1 million SNPs common between the TEDS sample and the HapMap3+ panel were included. The most recent GWAS summary statistics of educational attainment (EA4) and intelligence (IQ3) were used (Okbay et al., 2022; Savage et al., 2018).

To maximise the prediction of general cognitive ability, we combined the EA4 and IQ3 polygenic scores using SMTPred, which applies an ordinary least squares (OLS) weighting approach (Maier et al., 2018). In our sample, EA4 was weighted 0.08 and IQ3 0.05. The resulting combined score, termed the *polygenic g score*, correlated highly with IQ3 (r = 0.81) and EA4 (r = 0.87) polygenic scores in our sample. This polygenic g score was used as the primary predictor for all cognitive abilities, educational achievement, behaviour problems, and anthropometric outcomes in the present study.

### Statistical Analyses

The present study was pre-registered at https://osf.io/vhuyj/overview. Analyses were performed using RStudio 2023.09.1+494 with codes available on GitHub (https://github.com/YujingLinn/Cog-PGS).

Before carrying out the main analyses, we conducted sensitivity tests to examine potential effects of age, sex, zygosity, twin birth order, genotyping chip, and the first ten genomic principal components (PCs) on all phenotypes and family socioeconomic status measured across development. Sensitivity analyses of the polygenic g score were also conducted, except for age effects. Results are detailed in Appendix Table B1.

Sensitivity analyses revealed significant associations between several covariates and the phenotypes. Specifically, age, sex, zygosity, genotyping chip, and the first ten genomic PCs showed significant effects on about half of the phenotypes. Twin birth order showed no significant effects. For the polygenic g score, significant effects were detected for zygosity, genotyping chip, and the ninth and tenth PCs. Thus, we adopted a conservative approach by including age, sex, genotyping chip, and the first ten genomic PCs as covariates in all cross-sectional analyses for consistency, even though not all phenotypes were significantly associated with every covariate. Age was excluded as a covariate in longitudinal analyses. Since including zygosity as a covariate is relatively unconventional, we performed additional analyses stratifying the sample into monozygotic and dizygotic twins to examine whether the polygenic g score predictions were robust across zygosity groups.

#### Polygenic Score Prediction

Our main analysis was to examine associations between our polygenic g score and outcomes at each age using a subsample of unrelated individuals (one randomly selected twin per pair). All phenotypes were analysed, including both composite scores and their constituent components or specific test scores.

All continuous outcomes were standardised prior to analysis. Models included age, sex, genotyping chip, and the first ten PCs as covariates to control for batch effects and population structure. We report standardised beta coefficients for the polygenic g predictor. Incremental variance explained was calculated as the difference in R^2^ between the full model and a reduced model containing only covariates. Confidence intervals were estimated using percentile bootstrapping with 1000 iterations.

To ensure the robustness of our findings, we repeated all prediction analyses separately among females, males, monozygotic twins, and dizygotic twins.

#### Common Factor Analyses

Next, we examined the polygenic g score prediction of cross-time common factor of the phenotypes. We extracted cross-time common factors from repeated measures of the same construct, assuming these reflect stable underlying latent traits. Using multilevel structural equation modelling (SEM) to account for twin structure (i.e., family clustering), we conducted confirmatory factor analyses (CFA) for g, verbal ability, nonverbal ability, SDQ, anxiety, and ADHD. Whereas cross-sectional analyses used one randomly selected twin per family to ensure independence, SEM analyses included both twins from each pair to increase statistical power, retaining the full sample while appropriately adjusting for within-family non-independence. Common factors were extracted for both total scales and subscales where applicable.

For cognitive and educational outcomes, TEDS employed the most appropriate informant depending on the developmental stage. Cognitive measures at early ages were administered by parents, and then the children took the tests themselves. Educational achievement was mainly evaluated by teachers, while national exam scores were provided by parents. Because different informants were used at different ages (e.g., parent-administered tests in early childhood, self-administered tests in adolescence), the common factors for cognitive abilities and educational achievement reflect developmental changes over time as well as potential method variance associated with different informants.

For behaviour outcomes, multiple informants were used at the same age. For example, SDQ was reported by parents at ages 2, 3, 4, 7, 9, 12, 16, and 21; by teachers at ages 7, 9, and 12; and via self-report at ages 12, 16, 21, and 26. This multi-informant approach reflects developmental appropriateness: parent reports are most suitable in early childhood when children cannot report reliably, teacher reports provide complementary school-based perspectives during school age, and self-reports become increasingly valid as adolescents develop greater self-awareness and autonomy.

We therefore extracted both cross-rater common factors (combining all informants) and within-rater common factors when three or more assessments from the same informant were available for a given construct.

We then used the polygenic g score to predict these common factors and compared predictive validity against the age-specific cross-sectional predictions. Models included age, sex, genotyping chip, and the first 10 genomic principal components as covariates, with standardised beta coefficients and incremental R^2^ reported. All analyses were repeated separately for female and male subsamples.

#### Latent Growth Curve Model

We conducted latent growth curve analyses to examine how the polygenic g score predicts both baseline levels (intercept) and developmental trajectories (slope) across time. A positive association with the intercept indicates that higher polygenic g scores predict higher initial levels of the phenotype, while a positive association with the slope indicates that higher polygenic g scores predict steeper increases in the phenotype over time. These analyses were conducted for measures with repeated assessments across development, including general cognitive ability, verbal and nonverbal abilities, educational outcomes, the subscales of the SDQ, anxiety, ADHD, height, and BMI.

For measures with multiple potential informants, we prioritised consistency across developmental stages. For most phenotypes, we used parent reports before age 18 and self-reports from age 18 onwards. When parent reports were unavailable in childhood or adolescence, we prioritised teacher reports, followed by child reports; however, in most cases, child reports were used when parent reports were unavailable.

Missing data in the repeated measures were handled using Full Information Maximum Likelihood within the latent growth curve models (Enders & Bandalos, 2001). This approach uses all available data points for each participant, allowing inclusion of individuals with partially missing timepoints. Participants were included if they had data available for at least two timepoints. No additional imputation was performed for other variables included in the models.

Like the confirmatory factor analyses, all latent growth models used the full twin sample with multilevel modelling to account for family clustering, maximising statistical power. Sex was included as a covariate in models for the whole sample. We also conducted multi-group analyses to compare intercepts and slopes between females and males, with family clustering accounted for within each sex-stratified sample.

#### Profile Analysis of Extremely High and Extremely Low Polygenic g Scores

We examined the developmental trajectories of participants with extreme polygenic g scores, defined as scores above or below three standard deviations from the population mean.

To maximise sample size, we assigned polygenic g scores to the MZ co-twins of genotyped individuals, as only one twin per MZ pair was genotyped because MZ twins share identical genomes. This yielded 19 participants with scores three standard deviations above the mean and 14 participants with scores three standard deviations below the mean.

We plotted the observed values of cognitive, educational, and behaviour problem outcomes for these extreme groups across development (standardised for comparability). Socioeconomic status was also plotted alongside the trajectories for context, rather than included as a covariate, given the potential circularity between polygenic g scores and SES.

To formally compare differences in outcomes across development between extreme groups, we divided the full sample into deciles based on polygenic g scores and conducted independent samples t-tests comparing the top and bottom deciles at each age.

#### Nonlinearity Tests

Finally, to test whether the relationship between polygenic g scores and outcomes is linear, we conducted regression analyses including a quadratic term for the polygenic g score. A significant quadratic term would indicate nonlinearity, representing either accelerating or decelerating effects at the extremes of the distribution. Conversely, a non-significant quadratic term would support linearity, suggesting that high and low extremes differ only quantitatively, not qualitatively, from the rest of the distribution.

#### Multiple Testing Correction

We applied the false discovery rate (FDR) correction to account for multiple testing across all regression analyses. All reported p-values are FDR-adjusted values.

## Results

We focus primarily on cognitive, educational, and behaviour problem phenotypes in this section, with other phenotypes discussed as relevant. Full results for all phenotypes, including anxiety, ADHD, anthropometric outcomes, and those assessed at just one or two ages, are reported in the appendices. Descriptive statistics for all phenotypes and the polygenic g score are shown in Appendix Table B2 and correlation matrices in Appendix Figure C1.

### Polygenic g Score Prediction from Infancy to Early Adulthood

**Figure 1.**
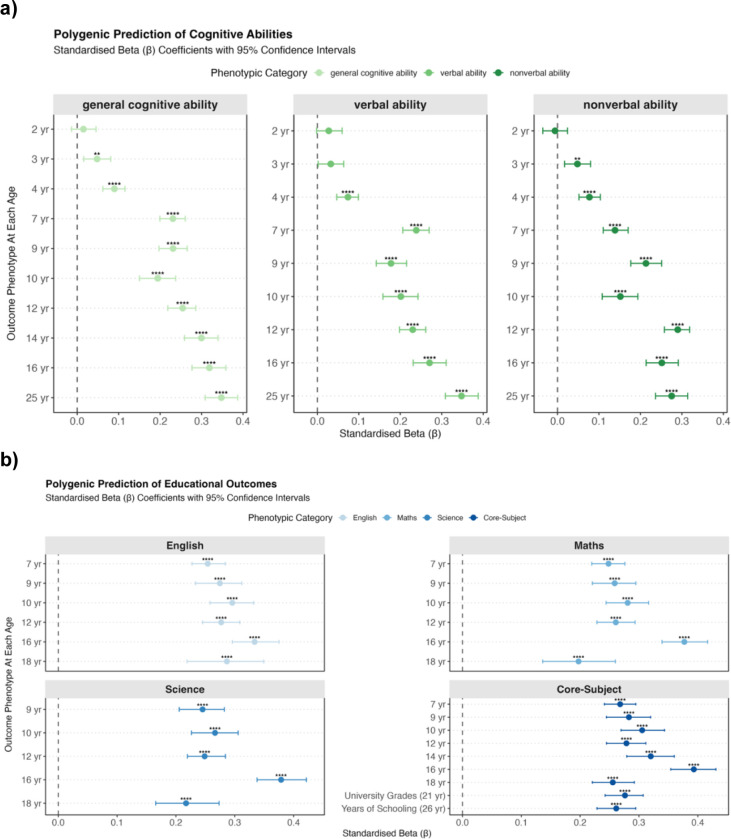

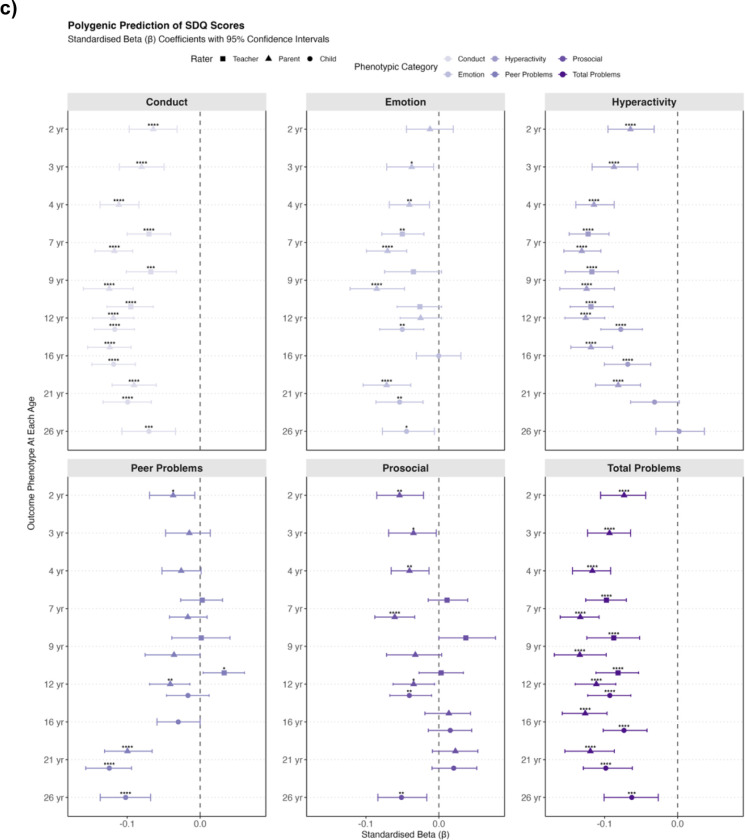
Polygenic g score prediction of cognitive abilities, educational achievement, and behaviour problems across development. Standardised beta (β) coefficients with 95% bootstrapped confidence intervals (1000 iterations) for the prediction of developmental outcomes by polygenic g score. Analyses were conducted in the unrelated sample by randomly selecting one twin from each pair. Panel a: General cognitive ability (g) composite, and domain-specific verbal and nonverbal ability composites measured from ages 2 to 25. Panel b: Educational achievement in English, mathematics, and science, and a core-subject composite. Science was first measured at age 9, while English and mathematics were also assessed at age 7. The core-subject composite was derived from English, mathematics, and science (where available) from ages 7 to 18, with general university grades used at age 21 and years of schooling at age 26. For plotting purposes, ‘core subject’ serves as an overarching category representing educational achievement and attainment through age 26. Panel c: Strengths and Difficulties Questionnaire (SDQ) five subscales (conduct problems, emotion problems, hyperactivity, peer problems, and prosocial behaviour) and total problems score measured from ages 2 to 26. For SDQ, the rater is indicated by a symbol shape (parent, teacher, or child). For other outcomes, performance-based measures were used, with parent ratings at early ages and self-reports or test-based assessments at later ages. The vertical dashed line at β = 0 represents no association. Asterisks denote statistical significance: * p < 0.05, ** p < 0.01, *** p < 0.001, **** p < 0.0001. Complete numerical results, including sex-stratified and zygosity-stratified analyses, are reported in Appendix Table B3. Individual tests comprising the cognitive composites are presented in Appendix Figures C2 and C3.

Polygenic g score significantly predicted most outcomes across phenotypes at most ages from childhood onwards. As shown in Figure 1a, for cognitive abilities, the prediction was weak or absent in infancy (ages 2 to 4). From childhood, the prediction increased steadily and reached a peak in early adulthood (age 25) with standardised beta coefficients of 0.35 (95% CI [0.31, 0.39]) for both g and verbal ability, and 0.28 [0.24, 0.31] for nonverbal ability.

We also examined polygenic g score prediction for the individual verbal and nonverbal tests used to construct the cognitive ability composites (see Appendix Figures C2 and C3). Similar patterns as in the composites were identified: weak or absent prediction in infancy that strengthened with age. Among verbal tests (Appendix Figure C2), the strongest prediction emerged for the Verbal Reasoning task from the Pathfinder battery at age 25 (β_standardised_ = 0.32 [0.28, 0.36]). Among nonverbal tests (Appendix Figure C3), the strongest prediction was observed for the Understanding Number task at age 16 (β_standardised_ = 0.31 [0.27, 0.35]).

For educational outcomes (see Figure 1b), polygenic g score associations were moderate at age 7 (β_standardised_ ≈ 0.25), increased through mid-adolescence. Prediction strength peaked at age 16 for GCSE grades: English (β = 0.33 [0.30, 0.38]), mathematics (β_standardised_ = 0.38 [0.34, 0.42]), science (β_standardised_ = 0.38 [0.34, 0.42]), and the core-subject composite (β_standardised_ = 0.39 [0.35, 0.43]). Polygenic g score associations declined for A-level, university grades and years of schooling.

For behaviour problems, we focused on SDQ measures (Figure 1c). Negative associations were observed for most behaviour problem measures across ages and raters. Significant predictions emerged in infancy for all SDQ subscales. For conduct problems and hyperactivity, associations strengthened slightly and peaked at approximately β_standardised_ = −0.12 and −0.13, respectively, in late childhood and adolescence, then weakened in early adulthood—becoming non-significant for hyperactivity but remaining significant for conduct problems. For emotional and peer problems, associations were largely non-significant from infancy to adolescence but became significantly negative though weak in adulthood. We also examined associations between the polygenic g score and the total problem scale. A consistent pattern of significant weak negative associations was observed across ages and raters, ranging from β_standardised_ = −0.06 to −0.13.

For prosocial behaviour, the polygenic g score explained negligible variance (all <0.5%) after accounting for age and sex effects. Associations were generally weak and inconsistent across raters and ages, with some being slightly negative early in life.

For other behaviour problems (anxiety in Appendix Figure C4; ADHD in Appendix Figure C5), weak negative associations were observed from infancy onward. Interesting patterns emerged for height and BMI, with non-significant associations until early adulthood, then positive associations with height and negative associations with BMI (Appendix Figures C6 and C7). For other miscellaneous outcomes, associations were largely positive for education- and cognitive-related phenotypes and showed mixed patterns for wellbeing-related phenotypes (Appendix Figure C8).

### Polygenic g Score Prediction of Cross-Time and Cross-Rater Latent Factor

We extracted cross-time latent factors to systematically examine polygenic g score prediction across age and raters. For parent, teacher, and child reports of behaviour problems, we extracted both cross-rater and within-rater latent factors.

All observed variables loaded significantly onto their respective latent constructs (Appendix Table B4), with standardised loadings ranging from 0.27 to 0.83. Model fit indices are provided in Appendix Table B5. For cognitive abilities (Appendix Figure C9), model fit was suboptimal compared to behaviour problems, yet predictions for the latent factors matched the strongest individual measure predictions for g (β_standardised_ = 0.39 [0.37, 0.41]), verbal ability (0.36 [0.34, 0.38]), and nonverbal ability (0.33 [0.31, 0.35).

For behaviour problems, model fits were mostly good. Cross-rater predictions showed that the polygenic g score was negatively associated with total SDQ problems (β_standardised_ = −0.19), conduct problems (−0.21), hyperactivity (−0.20), emotional problems (−0.11), peer problems (−0.08), and prosocial behaviour (−0.05; Appendix Figure C9). Cross-rater estimates approximated the average of within-rater estimates, with no clear pattern about which rater showed stronger prediction. Within-rater models tended to show slightly better fit, which is expected given that combining ratings across informants introduces additional method variance due to informant effects.(Achenbach et al., 1987; Glaser et al., 1997). Standardised path diagrams for all 39 models appear in Appendix Figure C10.

### Polygenic g Score Prediction of Developmental Trajectories

Beyond examining associations at individual ages and cross-time common factors, we used latent growth curve models to examine how the polygenic g score predicts both baseline levels (intercepts) and rates of developmental change (slopes). Only longitudinal measures were included for cognitive abilities, educational achievement, behaviour problem and anthropometric outcomes. The full results are presented in Appendix Table B6 for the full sample and in Appendix Table B7 for the sex-stratified sample.

At the baseline, the polygenic g score already displayed significant positive associations with cognitive, educational, and anthropometric outcomes. The strongest baseline predictions were for science achievement and core-subject achievement (both β_standardised_ = 0.35), followed by cognitive composites: g and verbal ability (both 0.10) and nonverbal ability (0.09). In contrast, all behaviour problem intercepts showed negative associations ranging from −0.02 to −0.17, indicating that children with higher polygenic g scores exhibited fewer behaviour problems.

Across development, the polygenic g score predicted steeper developmental increases for most cognitive and educational outcomes, including both g and verbal ability (β_standardised_ = 0.31), which were followed by science achievement (0.28), nonverbal ability (0.27), and core-subject achievement (0.13). Together, the positive baseline and growth effects for cognitive and educational outcomes implied that children with higher polygenic g scores started with higher cognitive and educational performance and continued to increase at a faster rate across development.

For behaviour problems, although half of the growth effects were negative and non-significant, the significant effects were primarily positive. For example, positive associations were found for ADHD and its subscales (β_standardised_ = 0.09 to 0.11), for ARBQ negative affect (0.06), and for SDQ hyperactivity (0.07). Peer problems showed the only significant negative slope (−0.09). Together with the negative baseline effects, the positive growth effects suggested that children with higher polygenic g scores begin with fewer behaviour problems but show slightly larger increases over time, gradually moving closer to the average developmental trajectories. In contrast, we found the opposite for BMI, which yielded a combination of a positive intercept (0.08) and a negative slope (−0.13), indicating that children with higher polygenic g scores started with a higher BMI, which increased at a slower rate with age.

Additionally, across models, consistently negative correlations between intercepts and slopes were identified, ranging from −0.31 to −0.68, indicating that individuals with higher baseline levels tended to show slower rates of increase over time.

### Polygenic g Score Prediction Across the Distribution

To examine whether polygenic g score prediction varies across the distribution, we first tested for nonlinear effects by including both linear and quadratic terms in the same model. Across all outcomes, no significant quadratic effects were observed in either the full sample or sex-stratified analyses (all incremental R^2^ < 0.01, all FDR-adjusted p > 0.05; Appendix Table B8). These results indicate that associations are linear throughout the distribution, with no evidence of stronger or weaker effects at the ends of the polygenic score distribution.

Second, we compared phenotypic outcomes between individuals at the distributional tails—those in the top versus bottom polygenic g score deciles (Appendix Table B9). For cognitive abilities, individuals at the top of the distribution consistently scored significantly higher than those at the bottom from around age 7 onwards, with occasional exceptions (e.g., spatial ability in adolescence and adulthood). Differences were non-significant in infancy. For educational achievement, all comparisons between distributional extremes were statistically significant. For behaviour problems, approximately half of the phenotypes showed non-significant differences between the top and bottom deciles. For example, peer problems across most ages and raters, as well as height and BMI, showed no significant differences between top and bottom deciles. The magnitude of decile differences generally corresponded to population-level prediction strength. Outcomes with stronger overall associations (such as cross-age and cross-rater composite scores) consistently showed significant differences between the top and the bottom polygenic g score deciles.

### Polygenic g Score Prediction for the Highest and Lowest Individuals

**Figure 2.**
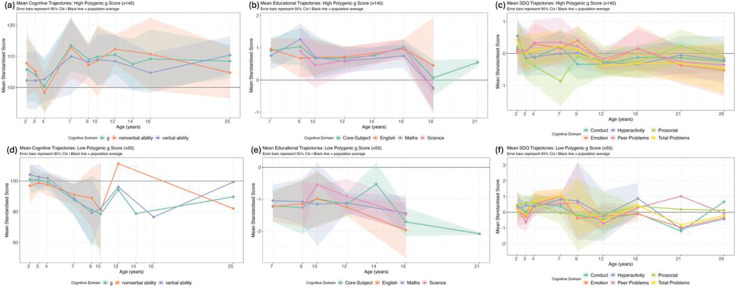
Mean developmental trajectories for individuals with the highest and lowest polygenic g scores. Mean trajectories across development for individuals with polygenic g scores >145 (panels a-c) and <55 (panels d-f). Panel a/d: General cognitive ability (g) composite and domain-specific composites (nonverbal and verbal ability). Panel b/e: educational achievement outcomes including English, mathematics, science, and core-subject composite (sum of English, mathematics, and science). Panel c/f: Strengths and Difficulties Questionnaire (SDQ) five subscales and total problem score. All measures are standardised to mean = 0, SD = 1, except for cognitive ability measures (mean = 100, SD = 15). Shaded areas represent 95% confidence intervals; absence of confidence intervals indicates that only one individual was measured at that age. For phenotypes with multiple raters, one rater per age is used: parent ratings before age 18 and child/self-ratings at age 18 and older. Black horizontal line at y = 0 represents the population average.

To examine outcomes at the distributional extremes in greater detail, we identified individuals with the highest (N = 19) and lowest (N = 14) polygenic g scores (Figure 2). Sample characteristics are presented in Appendix Table B2 and group comparisons in Appendix Table B10. Due to small sample sizes, most comparisons were not statistically significant. Notably, SES differed significantly between groups at all ages.

For cognitive abilities, both groups initially performed within one standard deviation (SD) of the population mean in infancy and early childhood (Figures 2a and 2d). By middle childhood, the high polygenic score group had IQ-equivalent scores of about 110. The low polygenic score group had IQ-equivalent scores between 80 and 90 in middle childhood but experienced substantial attrition; by adulthood, only one individual remained in the study.

Educational achievement yielded similar developmental trends. The high polygenic score group (Figure 2b) performed about one SD above the population mean, while the low polygenic score group performed about one SD below the mean (Figure 2e). For behaviour problems, neither group differed significantly from the population mean or from each other (Figures 2c and 2f). Mean trajectory comparisons for the additional phenotypic groups are presented in Appendix Figure C11. Individual trajectories with SES annotations for all participants with the highest and lowest polygenic g scores are shown in Appendix Figures C12 and C13, respectively.

### Polygenic g Score Prediction Among Subsamples

Polygenic g score predictions showed high consistency across sexes at each age, the latent factor across ages, developmental trajectories, across distributions, and among the most extreme scorers. Estimates were typically within one to two standard errors of each other, well within the range of overlapping 95% confidence intervals. Cognitive composites and individual cognitive tests showed nearly identical predictions for males and females. Educational outcomes showed similar patterns for both sexes, with predictions increasing through high school and declining thereafter. For behaviour problems, predictions were also comparable between sexes, with only occasional deviations (e.g., peer problems at age 21 showed β_standardised_ = −0.17 for females and −0.06 for males). On average, the differences in standardised beta coefficients between sexes were smaller than 0.01.

Zygosity comparisons likewise revealed minimal differences. Predictions were highly similar for monozygotic and dizygotic twins, with an average difference in standardised beta coefficients of only 0.01 across all phenotypes. The largest observed difference was for English achievement at age 16 (β_standardised_ = 0.37 for monozygotic twins vs. 0.24 for dizygotic twins), though such differences were rare exceptions rather than a consistent pattern.

## Discussion

Polygenic scores derived from adult intelligence and educational attainment continue to be the strongest predictors of cognitive development. By combining IQ3 and EA4, we created an adult polygenic g score to chart cognitive development from infancy (age 2) to early adulthood (age 26). Our polygenic g score improved prediction from 9% variance explained using IQ3 alone to 12% for g at age 25. Prediction was even higher for cross-time latent factors, reaching 15% for g. Additionally, for educational achievement, the polygenic g score predicted up to 16% of variance at age 16, and up to 2% for behaviour problems and anthropometric outcomes.

The polygenic g score predicts cognitive outcomes as early as age 4 (β_standardised_ = 0.07 to 0.09). Although the prediction was largely negligible in infancy, it increased steadily from childhood to early adulthood, peaking at β_standardised_ = 0.35 at age 25, supporting our first two hypotheses on the significant and increased polygenic score prediction with age for cognitive abilities. These age-related increases in PGS prediction are consistent with both twin and genomic studies. For example, twin heritability estimated in TEDS increased from 41% at age 9 to 66% at age 17 (Haworth et al., 2010). Another study extends the finding into late midlife and shows that cognitive heritability remains stable since young adulthood (Lyons et al., 2017). A similar pattern was identified in genomic studies using PGS (Allegrini et al., 2019; Gustavson et al., 2025). This pattern is known as the Wilson effect, which refers to the increasing heritability of IQ with age (Bouchard, 2013).

Several mechanisms help explain this phenomenon. First, discovery GWA studies of cognitive abilities are conducted in adults; thus, the SNP effect estimates in these GWA studies more closely match the genetic architecture of cognitive outcomes in later developmental stages than in infancy or early childhood. Second, the phenotypic measures become more stable and reliably assessed with age, which limits how well the polygenic g score derived from GWAS of adults can predict cognitive abilities earlier in life. For example, the phenotypic correlation of intelligence between ages 2 and 25 was modest (r = 0.11), but increased substantially by late adolescence, reaching r = 0.65 between ages 16 and 25. Third, increasing active and evocative gene–environment correlation amplifies genetic effects over time, as individuals increasingly select, evoke, and create environments that align with their genetic propensities (Plomin, 1994).

Despite potential constraints in early-age measurement, the polygenic g score is still one of the best predictors of cognitive development. By combining IQ3 and EA4, we introduced additional genetic signals relevant to cognitive abilities (Plomin & von Stumm, 2018), adding 3% variance explained from 9% using IQ3 alone to 12% in predicting g at age 25. We achieve even higher predictive power by extracting a cross-time common latent factor of g, verbal ability, and nonverbal ability—15%, 13%, and 11%, respectively. This is to be expected because genetic influences on cognition are stable across development (Gustavson et al., 2025). The latent factors had substantial loadings from all ages (0.45–0.72), indicating that even the earliest cognitive assessments contribute meaningfully to this stable variance. Latent factors aggregate information across repeated measurements, reducing age-specific measurement error and isolating the stable component of cognitive variation.

In addition, our latent growth curve models captured both baseline differences and developmental changes. Children with higher polygenic g scores showed higher baseline cognitive performance and steeper growth rates, consistent with increasing gene–environment correlation over time. These findings illustrate that the influence of polygenic g is not limited to predicting outcomes at isolated ages but also characterises the overall developmental trajectory of cognitive abilities from early childhood to adulthood.

We next examined educational achievement, behaviour problems, and anthropometric measures. Polygenic g score predicted most trait domains, supporting the third hypothesis. The strongest prediction was for educational achievement, consistent with previous research (Wilding et al., 2024). Prediction of educational increased until the end of secondary school, peaking at 16% for GCSE achievement, and then declined to approximately 7% for years of schooling at age 26. Similar developmental patterns were reported in independent research, with the later decline attributed to range restriction because after secondary school, only a subset of the sample entered higher education, reducing variability and attenuating prediction (Kvalvik et al., 2025).

As with cognitive traits, polygenic g predicted both higher baseline educational achievement and steeper growth than cognitive abilities. In contrast, predictions for behaviour problems and anthropometric outcomes were negligible to weak and showed less consistency. Behaviour problems showed negative baseline associations and small positive slope estimates, indicating that children with higher polygenic g scores began with fewer behaviour problems but exhibited slightly greater increases over time. This pattern might reflect regression to the mean rather than substantive developmental change. No consistent sex differences were observed.

Furthermore, prediction across the distribution of polygenic g scores was linear, in line with our fourth hypothesis. The absence of significant quadratic effects indicates that individuals at the end of the distribution differ quantitatively instead of qualitatively from the rest of the sample. At the distributional extremes of the polygenic g scores (+/−three standard deviations), individuals who were three standard deviations above the mean showed, on average, around one to two standard deviations advantage in cognitive and educational outcomes in adulthood. Behaviour problems and anthropometric outcomes showed almost no meaningful differences between extremes. Finally, consistent with our fifth hypothesis, we found no evidence for sex differences in the prediction of the polygenic g score across outcomes.

Our study has several limitations. First, prediction for nonverbal ability was weaker than for verbal ability, likely because GWA studies are more heavily weighted toward verbal measures. Even after incorporating EA4, some genetic signals for nonverbal ability may not be fully captured (Procopio et al., 2025). Future GWA studies could benefit from a greater balance in verbal and nonverbal abilities. Second, our g composite was constructed by averaging verbal and nonverbal abilities. Although this composite performed well, hierarchical phenotypic models would allow us to estimate g more precisely from the underlying tests and could improve prediction. Third, discovery GWA studies rely on common variants. Recent evidence suggests that rare genetic variants contribute substantially to cognitive differences and could improve prediction with whole-genome sequencing (Wainschtein et al., 2025). Finally, caution is needed when applying composite polygenic g scores in within-family contexts. Both IQ3 and EA4 contain family-level genetic contributions, meaning prediction may be attenuated among siblings (Lin et al., 2025).

As twin analyses suggested an increased heritability of intelligence and cognitive abilities from childhood to young adulthood, molecular genetic studies have made use of this finding by focusing on the genetics of adults. While twin designs rely on phenotypic measurements at each developmental stage to estimate heritability, GWA- and PGS-based approaches can quantify genetic liability from conception onward. After decades of research, we are now able to go back to the flip side using adult molecular genetics outcomes to chart child cognitive development as it is linked genetically to adult cognitive abilities. Our results show that prediction begins as early as age 4, increases steadily through development, and is especially powerful in estimating growth trajectories. Importantly, this predictive utility does not depend on a causal explanation of individual SNP effects, but rather on the aggregation of genetic signals associated with the phenotype (Lin et al., 2025; Plomin & von Stumm, 2022).

Together, we show that combining two highly predictive polygenic scores yields substantial insights into cognitive developmental trajectories from infancy to early adulthood. Outperforming other early-life predictors, polygenic scores fixed at conception continue to be one of the strongest predictors available for long-term cognitive development.

## Supplementary Material

Supplement 1

Supplement 2

Supplement 3

Supplement 4
