# Supplementary material for: Charting the cognitive development of children using adult ‘polygenic g scores’
Source: bioRxiv. 2025 Dec 23:2025.12.19.695378. Preprint. [Version 1] doi: 10.64898/2025.12.19.695378 (PMC12767516; doi:10.64898/2025.12.19.695378)
Supplement: Supplement 1 [file media-1.pdf]

Charting the cognitive development of children using adult 'polygenic g scores'

Appendices

Yujing Lin [1], Robert Plomin [1]
[1] Social, Genetic and Developmental Psychiatry Centre, King's College London

The Appendices are organised into three sections: Appendix A presents the supplementary phenotypic measures; Appendix B presents the supplementary tables; and Appendix C presents the supplementary figures.

Table of Contents

Appendix A. Supplementary Phenotypic Measures ***4***
ADHD Symptoms..4
Anxiety..4
Height and BMI 4

Additional Measures..**4**
Reading and Mathematics Environment..4
PISA Measures 4
Spatial and Mathematical Anxiety...5

Appendix B. Supplementary Tables (provided in a separate spreadsheet)........ ***7***
All supplementary tables referenced in the main text are compiled in the accompanying Excel spreadsheet (AppendixB_SupTables.xlsx). The following list provides an overview of the tables included. 7

Table B1. Sensitivity Analyses Examining Effects of Age, Sex, Zygosity, Twin Birth Order, Genotyping Chip Type, and the First Ten Principal Components on Phenotypes and Polygenic g Score **7**

Table B2. Sample Characteristics for Phenotypic and Genotypic Outcomes Across the Whole Sample and Subsamples...**7**

Table B3. Regression Results of Polygenic g Score Predicting Cognitive, Educational, and Behavioural Outcomes for Whole Sample, Sex-Stratified, and Zygosity-Stratified Samples **7**

Table B4. Standardised Factor Loadings for Cross-Age Latent Factors: Cross-Rater and/or Within-Rater Confirmatory Factor Analyses of Behavioural and Cognitive Outcomes **7**

Table B5. Confirmatory Factor Analysis Model Fit Indices and Polygenic g Score Prediction of Cross-Age Latent Factors..**7**

Table B6. Latent Growth Curve Model Fit Indices and Polygenic g Score Prediction of Latent Intercepts and Slopes **7**

Table B7. Sex-Stratified Latent Growth Curve Model Fit Indices and Polygenic g Score Prediction of Latent Intercepts and Slopes **7**

Table B8. Polynomial Regression Results Testing Nonlinear Associations Between Polygenic g Score and Cognitive, Educational, and Behavioural Outcomes **7**

Table B9. Mean Differences in Cognitive, Educational, and Behavioural Outcomes Between Top and Bottom Deciles of Polygenic g Score 7

Table B10. Phenotypic Differences Between High and Low Polygenic g Score Groups 7

Appendix C. Supplementary Figures 8

All supplementary figures referenced in the main text are presented in this appendix and in two accompanying compiled PDF files: AppendixC_SupFigureC1_CorrMatrices.pdf and AppendixC_SupFigureC10_CFAplots.pdf. 8

Figure C1. Heatmaps of Pearson correlations between the polygenic g score and observed phenotypic outcomes (provided in a separate PDF file) 8

Figure C2 to C9. Polygenic g score prediction of phenotypic outcomes in the whole sample 9

Figure C2. Polygenic prediction of individual verbal test scores 10
Figure C3. Polygenic prediction of individual nonverbal test scores 11
Figure C4. Polygenic prediction of anxiety subscales and total score 12
Figure C5. Polygenic prediction of ADHD subscales and total score 13
Figure C6. Polygenic prediction of height 14
Figure C7. Polygenic prediction of weight at birth and BMI from age 3 onward 15
Figure C8. Polygenic prediction of other outcomes (educational, environmental, behavioural, and wellbeing measures) 16
Figure C9. Polygenic prediction of common latent factors across age and raters 17

Figure C10. Confirmatory factor analysis (CFA) path diagrams (provided in a separate PDF file) 18

C10-1. General Cognitive Ability (g) − Cross Rater 19
C10-2. Verbal Ability − Cross Rater 19
C10-3. Nonverbal Ability − Cross Rater 19
C10-4. ARBQ Shyness − Cross Rater 19
C10-5. ARBQ Fear − Cross Rater 19
C10-6. ARBQ Obsessive-Compulsive − Cross Rater 19
C10-7. ARBQ Negative Affect − Cross Rater 19
C10-8. ARBQ Negative Cognition − Cross Rater 19
C10-9. ARBQ Anxiety Total − Cross Rater 19
C10-10. Conners Inattention − Cross Rater 19
C10-11. Conners Inattention − Parent Only 19
C10-12. Conners Hyperactivity-Impulsivity − Cross Rater 19
C10-13. Conners Hyperactivity-Impulsivity − Parent Only 19
C10-14. Conners Total − Cross Rater 19
C10-15. Conners Total − Parent Only 19
C10-16. SDQ Conduct − Parent Only 19
C10-17. SDQ Conduct − Teacher Only 19
C10-18. SDQ Conduct − Child Only 19
C10-19. SDQ Emotion − Parent Only 19
C10-20. SDQ Emotion − Teacher Only 19
C10-21. SDQ Emotion − Child Only 19
C10-22. SDQ Hyperactivity − Parent Only 19
C10-23. SDQ Hyperactivity − Teacher Only 19
C10-24. SDQ Hyperactivity − Child Only 19
C10-25. SDQ Peer Problems − Parent Only 19
C10-26. SDQ Peer Problems − Teacher Only 19
C10-27. SDQ Peer Problems − Child Only 19
C10-28. SDQ Prosocial − Parent Only 19
C10-29. SDQ Prosocial − Teacher Only 19
C10-30. SDQ Prosocial − Child Only 19
C10-31. SDQ Total Problems − Parent Only 19

C10-32. SDQ Total Problems − Teacher Only 19
C10-33. SDQ Total Problems − Child Only 20
C10-34. SDQ Conduct − Cross Rater 20
C10-35. SDQ Emotion − Cross Rater 20
C10-36. SDQ Hyperactivity − Cross Rater 20
C10-37. SDQ Peer Problems − Cross Rater 20
C10-38. SDQ Prosocial − Cross Rater 20
C10-39. SDQ Total Problems − Cross Rater 20

Figure C11. Mean developmental trajectories for behavioural and anthropometric outcomes in individuals with high and low polygenic g scores 21

Figure C12. Individual developmental profiles for individuals with high polygenic g scores (>145) 22

Figure C13. Individual developmental profiles for individuals with low polygenic g scores (<55) 24

References 26

Appendix A. Supplementary Phenotypic Measures

ADHD Symptoms

Attention-Deficit/Hyperactivity Disorder (ADHD) symptoms were assessed using the Conners Rating Scale based on Diagnostic and Statistical Manual of Mental Disorders, Fourth Edition (DSM-IV) criteria (Conners, 2003, 2008). The standard 18-item scale comprises two 9-item subscales measuring hyperactivity and inattentional behaviours.

Parent ratings were obtained at ages 8, 12, 14, 16, and 21, teacher ratings at age 14, and self-ratings at ages 14 and 21, all using the full 18-item scale. At age 26, only the 9-item inattention subscale was administered via self-report (with 2 additional quality control items included in the questionnaire).

Anxiety

Anxiety was assessed using the Anxiety-Related Behaviours Questionnaire (ARBQ), a measure designed to capture anxiety symptoms and anxiety-related temperamental traits in the general population (Eley et al., 2003). The ARBQ comprises five subscales: negative cognition, negative affect, fear, social anxiety (shyness), and obsessive-compulsive behaviours (OCB). Psychometric properties and construct validity of the measure have been reported in previous TEDS publications (Hallett et al., 2009; Trzaskowski et al., 2012).

Parent ratings were collected at ages 3 (5 items), 4 (12 items), 7 (21 items), 9 (20 items), and 16 (19 items). Teacher ratings (17 items) were collected at age 7. The questionnaire's length and content were adapted for developmental appropriateness by the TEDS researchers. At age 3, only the fear and social anxiety subscales were assessed; the OCB, negative affect, and negative cognition subscales were added at age 4. Total anxiety scores were calculated as the standardised mean of the available subscales for each participant at each age.

Height and BMI

Parent-reported birth weight (grams) and length (centimetres) were collected retrospectively during the first contact at 18 months. Height was also collected in centimetres at ages 7, 12, 14, 16, 21, and 26. Body Mass Index (BMI) was calculated at ages 3, 4, 7, 12, 14, 16, 21, and 26.

Additional Measures

Reading and Mathematics Environment

Reading and mathematics environments were assessed at ages 10 and 12 using child-completed web-based questionnaires adapted from the National Assessment of Educational Progress (NAEP) Grade 4 student background questionnaires for mathematics (https://nces.ed.gov/nationsreportcard/pdf/05BQstudentG4math.pdf) and reading (https://nces.ed.gov/nationsreportcard/pdf/05BQstudentG4read.pdf). The adapted questionnaire included 15 items assessing children’s experiences with mathematics and reading environments at home and school.

PISA Measures

At age 16, several measures were adapted from the Programme for International Student Assessment (PISA; OECD: https://www.oecd.org/en/about/programmes/pisa.html), drawing on items from the 2000, 2003, and 2006 student questionnaires. The measures included homework

behaviours (5 items), attitudes toward school (4 items), mathematics self-efficacy (8 items), mathematics interest (3 items), and time spent on mathematics (3 items).

Spatial and Mathematical Anxiety

At age 18, spatial and mathematical anxiety was measured using the Self-Perceived Ability and Anxiety (SPAA) questionnaire, developed by the TEDS team as part of the spatial ability testing battery based on existing scales (Hopko et al., 2003; Lawton, 1994). The questionnaire included subscales assessing spatial ability (8 items), spatial anxiety (10 items), and mathematics anxiety (9 items).

Financial and Socioeconomic Measures

At age 21, participants completed self-report measures assessing financial wellbeing, literacy, and attitudes. Financial wellbeing was measured using a 5-item shortened version of the Contentment with Life Assessment Scale (CLAS), assessing satisfaction with their financial situation (Lavallee et al., 2007). Financial literacy regarding products was measured using a 13-item adaptation of the OECD Financial Literacy instrument, shortened from 15 items with an additional quality control item (https://www.oecd.org/en/topics/financial-education.html). General money attitudes and behaviours were assessed using six relevant items from the OECD scale.

At age 26, socioeconomic outcomes were captured using derived indicators of economic vulnerability and a twin-specific SES composite. Economic vulnerability was quantified as an ordinal score (0–4), summing indicators of NEET status (not in education, employment, or training), low income, receipt of benefits, presence of children, and zero-hours employment. NEET status was coded as a binary variable, indicating whether the participant was not in education, employment, or training. The twin SES composite was a standardised continuous measure derived from educational attainment, income level, and economic vulnerability, calculated to reflect the individual twin's socioeconomic status. All variables were devised by TEDS researchers using survey responses and administrative coding according to the Standard Occupational Classification 2000 (https://www.ons.gov.uk/methodology/classificationsandstandards/standardoccupationalclassificationsoc/socarchive).

Wellbeing and Noncognitive Traits

At age 16, wellbeing and non-cognitive traits were assessed using a combination of established self-report scales. The measures included a 21-item life satisfaction scale, 6-item hopefulness scale, 6-item gratitude scale, 7-item curiosity scale with subscales for exploration and flow, 4-item subjective happiness scale, 13-item grit and ambition scale, 6-item optimism scale, and a 10-item academic self-concept scale.

Life satisfaction was measured using the Multidimensional Students' Life Satisfaction Scale, assessing satisfaction with family, friends, living environment, school, and self, as well as overall life satisfaction (Huebner, 1994). Hopefulness was assessed using the Children's Hope Scale, comprising agency and pathways subscales (Snyder et al., 1997). Additional wellbeing measures included Subjective Happiness (Lyubomirsky & Lepper, 1999), Gratitude (McCullough et al., 2002), and Curiosity (Kashdan et al., 2004), which captured both exploration and flow components.

Non-cognitive traits were measured using Ambition and Grit scales (Duckworth et al., 2007; Duckworth & Quinn, 2009), with Grit subdivided into consistency of interests and perseverance of effort subscales. Optimism was assessed using the Life Orientation Test–Revised (Scheier et al., 1994), and

Academic Self-Concept using a shortened version of Perceptions of Self as Learners and Problem Solvers scale (Burden, 1998).

Finally, composite indices of psychological wellbeing and subjective wellbeing were derived from relevant subsets of these wellbeing measures, including life satisfaction, hopefulness, gratitude, curiosity, and subjective happiness.

At age 21, several measures overlapped with those administered at age 16, alongside additional assessments of self-control, purpose in life, and depressive symptoms. Self-control was measured using a 6-item version of the Brief Self-Control Scale (Tangney et al., 2004), shortened from the original 13-item measure. The Consideration of Future Consequences Scale was abbreviated to 4 items, with the addition of a quality control item (Strathman et al., 1994). Life goals were assessed using a 9-item adaptation of the GOALS questionnaire, translated into English and focused on fulfilment- and relationship-oriented goals, excluding items related to social status or likelihood of success (Pöhlmann & Brunstein, 1997). Purpose in Life was measured using a 5-item short form of the Purpose in Life Test (Crumbaugh & Maholick, 1964). Ambition was assessed using the same 5-item Grit measure previously administered at age 16. Finally, depressive symptoms were measured using an 8-item version of the Short Moods and Feelings Questionnaire, adapted from the original 13-item version with one additional quality control item (Angold et al., 1995).

At age 26, wellbeing and mental health were re-evaluated. Subjective wellbeing (quality of life) was measured using a 3-item scale including two euthymic (positive emotion) items and one eudaimonic (meaning) item, adapted from the UK Biobank and Genetic Links to Anxiety and Depression (GLAD) Study mental health questionnaires. Depressive symptoms were assessed using the 13-item Moods and Feelings Questionnaire, a validated screening measure for current depressive symptoms, also including a quality control item (Angold et al., 1995).

Appendix B. Supplementary Tables (provided in a separate spreadsheet)

All supplementary tables referenced in the main text are compiled in the accompanying Excel spreadsheet (AppendixB_SupTables.xlsx). The following list provides an overview of the tables included.

Table B1. Sensitivity Analyses Examining Effects of Age, Sex, Zygosity, Twin Birth Order, Genotyping Chip Type, and the First Ten Principal Components on Phenotypes and Polygenic g Score

Table B2. Sample Characteristics for Phenotypic and Genotypic Outcomes Across the Whole Sample and Subsamples

Table B3. Regression Results of Polygenic g Score Predicting Cognitive, Educational, and Behavioural Outcomes for Whole Sample, Sex-Stratified, and Zygosity-Stratified Samples

Table B4. Standardised Factor Loadings for Cross-Age Latent Factors: Cross-Rater and/or Within-Rater Confirmatory Factor Analyses of Behavioural and Cognitive Outcomes

Table B5. Confirmatory Factor Analysis Model Fit Indices and Polygenic g Score Prediction of Cross-Age Latent Factors

Table B6. Latent Growth Curve Model Fit Indices and Polygenic g Score Prediction of Latent Intercepts and Slopes

Table B7. Sex-Stratified Latent Growth Curve Model Fit Indices and Polygenic g Score Prediction of Latent Intercepts and Slopes

Table B8. Polynomial Regression Results Testing Nonlinear Associations Between Polygenic g Score and Cognitive, Educational, and Behavioural Outcomes

Table B9. Mean Differences in Cognitive, Educational, and Behavioural Outcomes Between Top and Bottom Deciles of Polygenic g Score

Table B10. Phenotypic Differences Between High and Low Polygenic g Score Groups

Appendix C. Supplementary Figures

All supplementary figures referenced in the main text are presented in this appendix and in two accompanying compiled PDF files: AppendixC_SupFigureC1_CorrMatrices.pdf and AppendixC_SupFigureC10_CFAplots.pdf.

Figure C1. Heatmaps of Pearson correlations between the polygenic g score and observed phenotypic outcomes (provided in a separate PDF file)

All 10 correlation matrices are compiled in a single PDF file (AppendixC_SupFigureC1_CorrMatrices.pdf). Each page presents a heatmap of Pearson correlation coefficients among repeated measures or composite variables within a trait domain. Above the diagonal, the shape and colour indicate the strength and direction of the correlation; below the diagonal, the corresponding correlation coefficients are displayed.

The heatmaps include correlations between the polygenic g score and:

1. Cognitive ability composites — general cognitive ability (g), verbal ability, and nonverbal ability at each age
2. Individual verbal tests administered at each age
3. Individual nonverbal tests administered at each age
4. Educational achievement and attainment outcomes at each age
5. Strengths and Difficulties Questionnaire (SDQ) subscales and total problem score at each age
6. Anxiety-Related Behaviours Questionnaire (ARBQ) subscales and total anxiety score at each age
7. Conners Rating Scale subscales and total ADHD score at each age
8. Anthropometric traits — height and BMI at each age and birth weight
9. Additional educational, environmental, behavioural, and wellbeing measures, including school and home environment indicators at each age
10. Common latent factors within and across raters across age

Figure C2 to C9. Polygenic g score prediction of phenotypic outcomes in the whole sample

Figures C2 to C9 present the standardised beta coefficients and 95% bootstrapped confidence intervals, estimated using percentile bootstrapping with 1000 iterations. Analyses were conducted in the unrelated sample by randomly selecting one twin from each pair. Each figure corresponds to a specific trait domain. Raters are specified in the plot when multiple raters are available for the same measure. When only one rater is present across ages (typically the child), or when rater differences are not relevant (e.g., height and BMI), the rater is not specified. Please refer to the measure descriptions for abbreviations of the variable names.

The main results for general cognitive ability, verbal and nonverbal ability composites, educational outcomes, and behavioural outcomes (SDQ) are presented in the main text (Figure 1). Numerical estimates, model variance explained, and statistical significance values for these analyses, as well as sex-stratified and zygosity-stratified results, are reported in Table B3. The following list provides an overview of Figures C2 to C9:

- Figure C2. Individual verbal test scores
- Figure C3. Individual nonverbal test scores
- Figure C4. Anxiety subscales and total score
- Figure C5. ADHD subscales and total score
- Figure C6. Height
- Figure C7. Birth weight and BMI from age 3 onward
- Figure C8. Other outcomes (educational, environmental, behavioural, and wellbeing measures)
- Figure C9. Common latent factors across age and raters

Figure C2. Polygenic prediction of individual verbal test scores

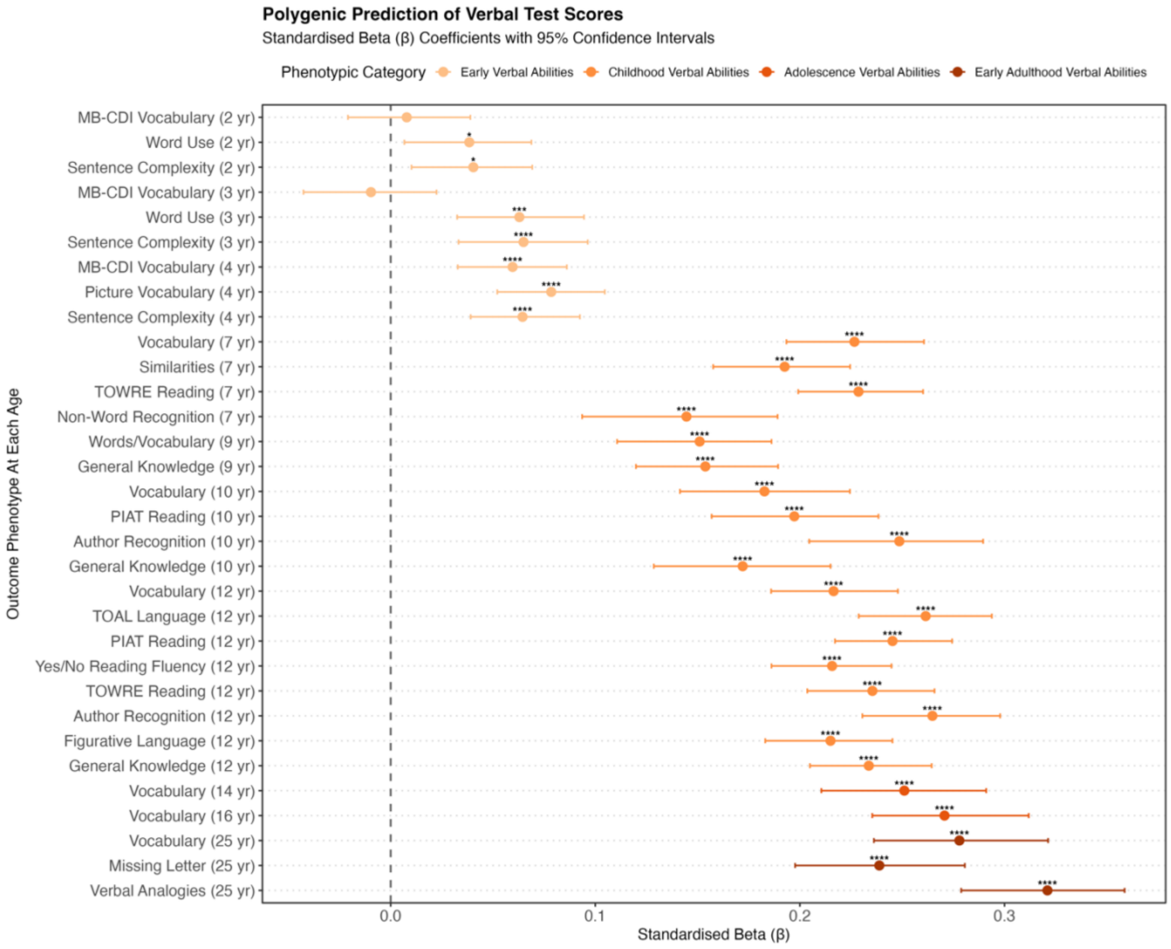

Figure C3. Polygenic prediction of individual nonverbal test scores

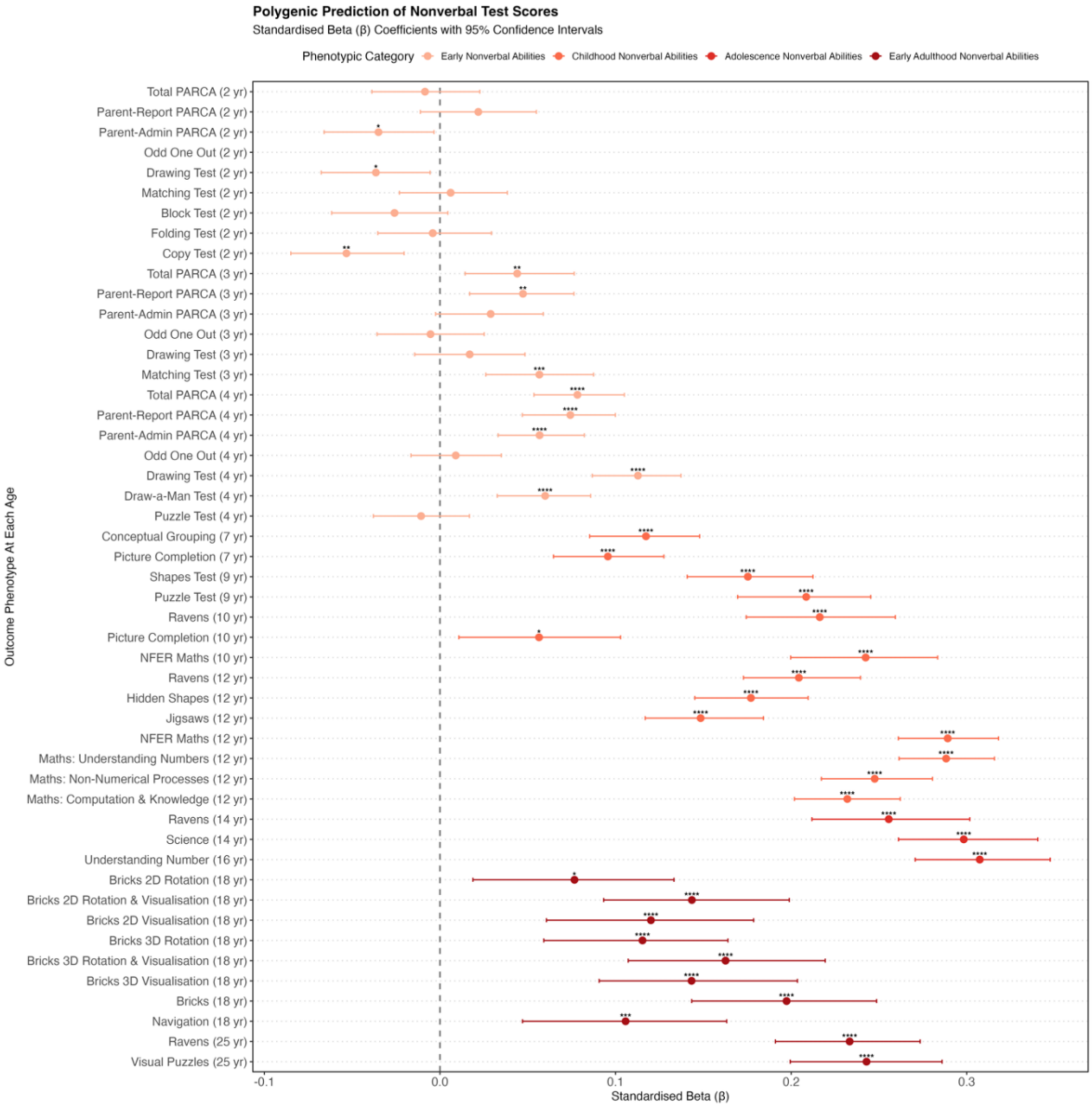

Figure C4. Polygenic prediction of anxiety subscales and total score

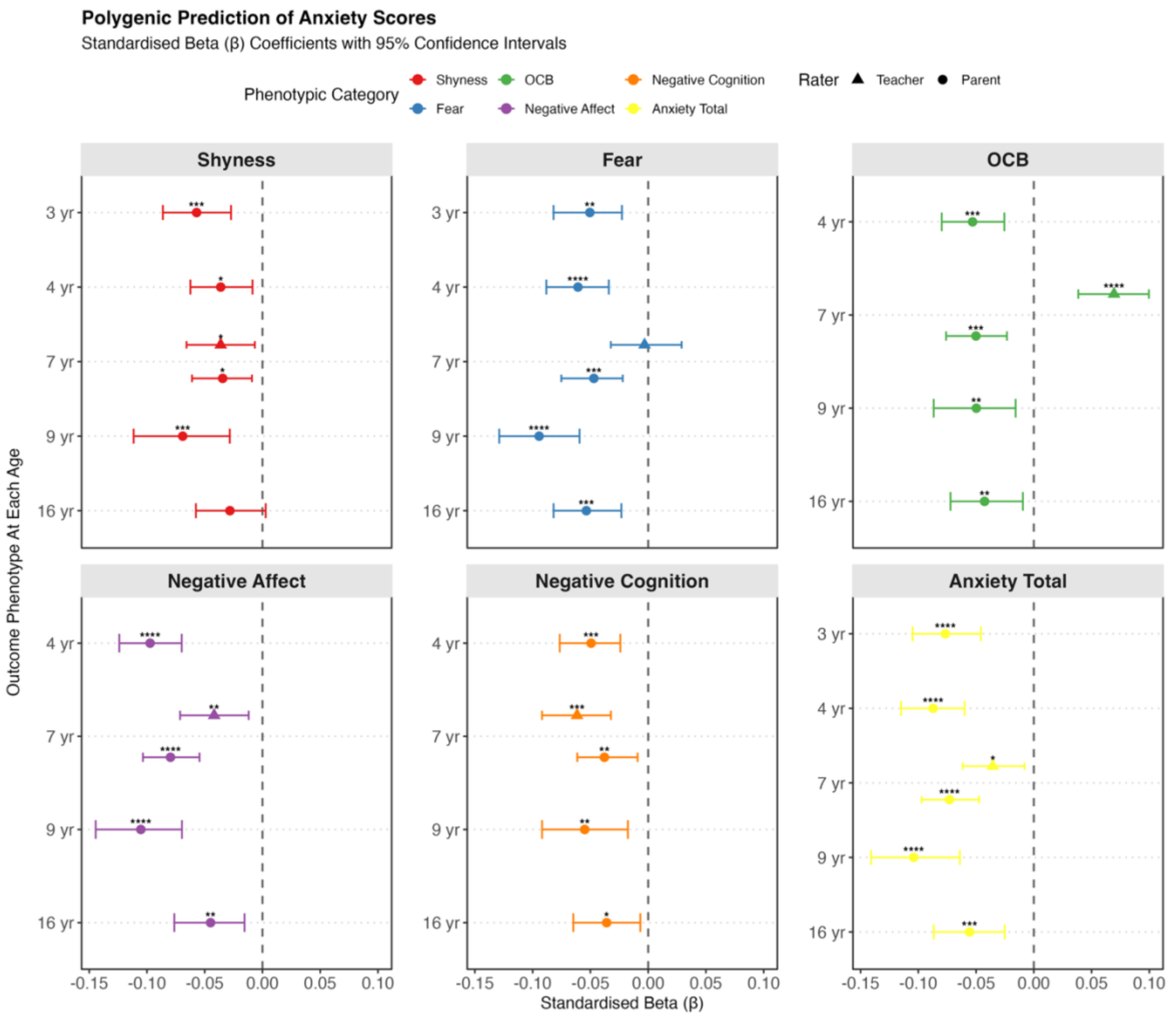

Figure C5. Polygenic prediction of ADHD subscales and total score

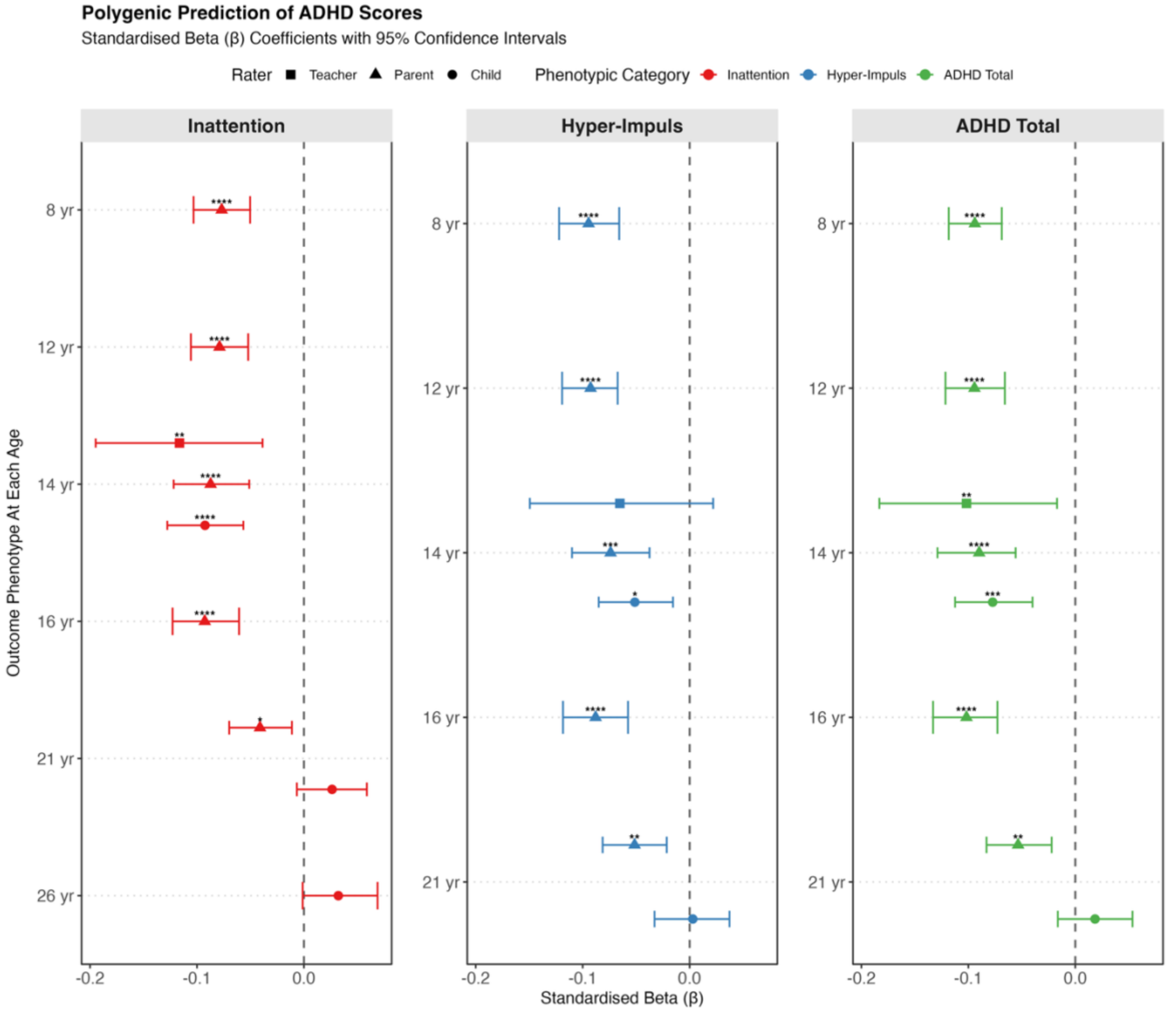

Figure C6. Polygenic prediction of height

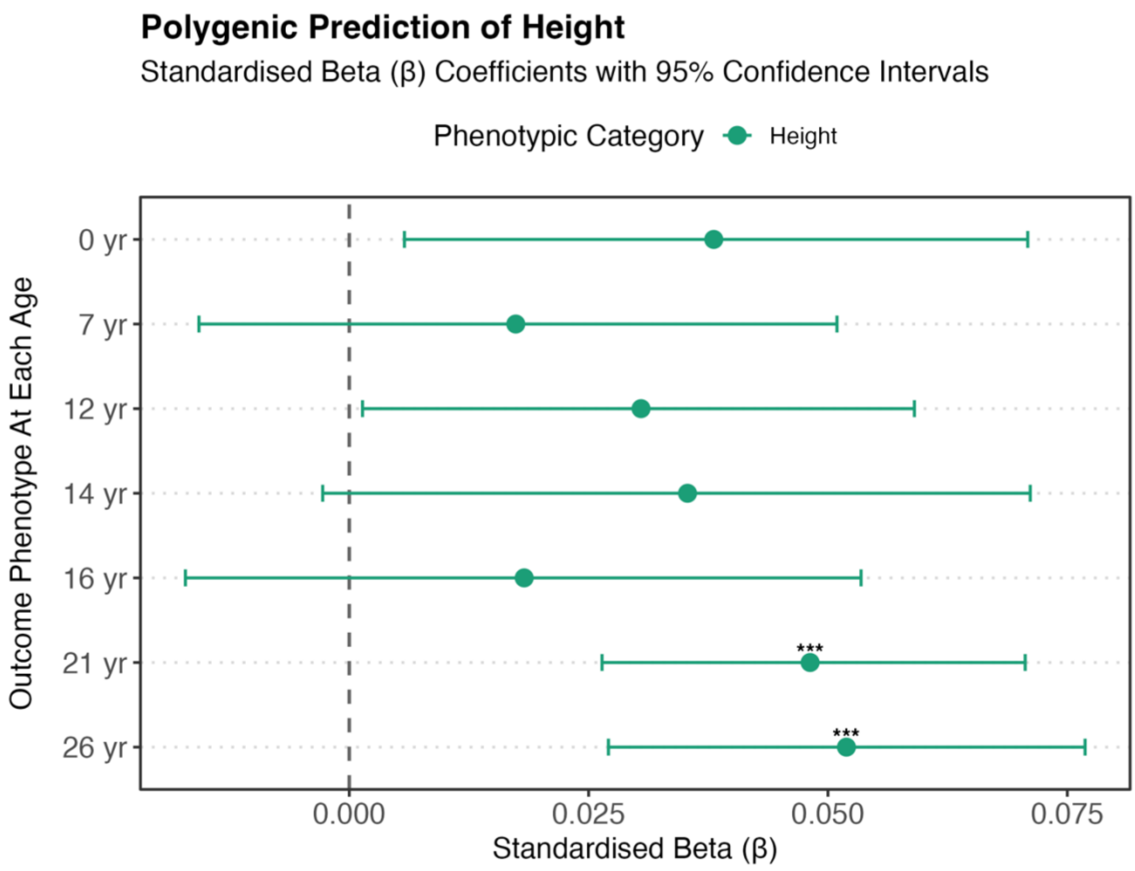

Figure C7. Polygenic prediction of weight at birth and BMI from age 3 onward

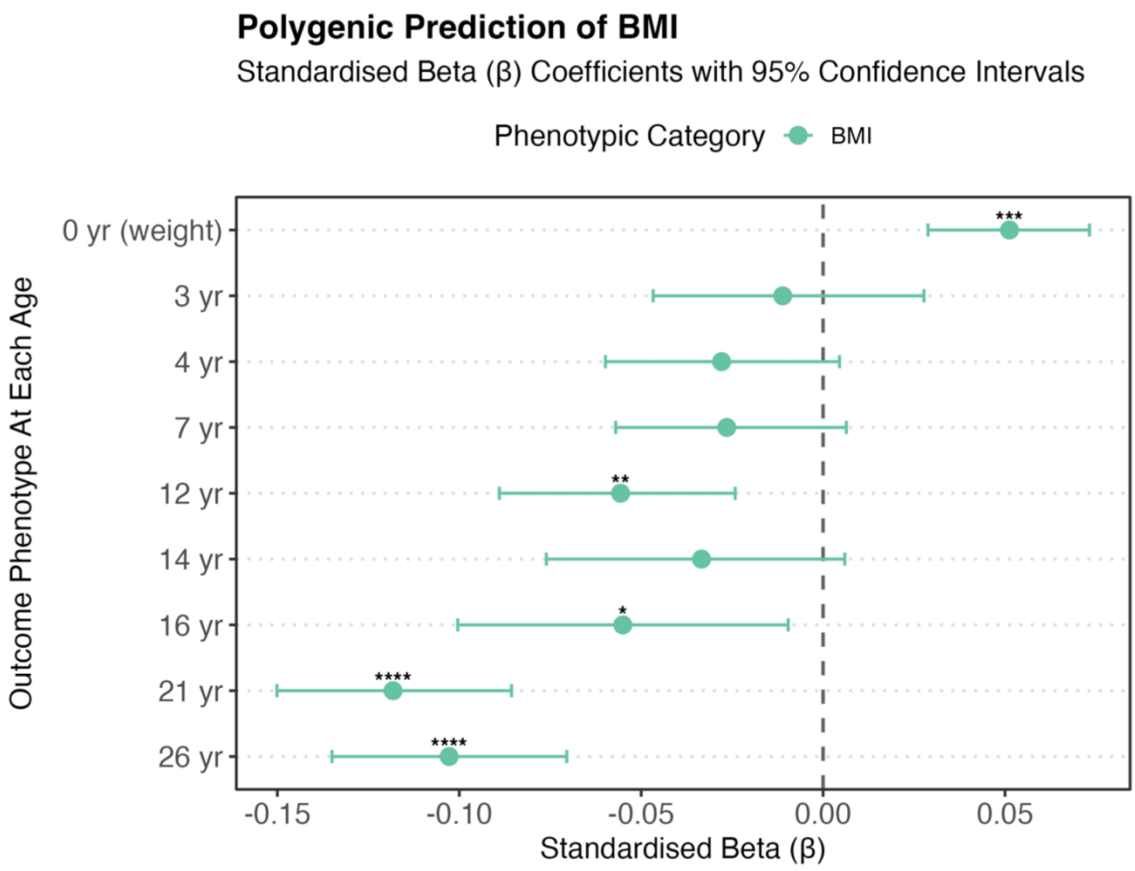

Figure C8. Polygenic prediction of other outcomes (educational, environmental, behavioural, and wellbeing measures)

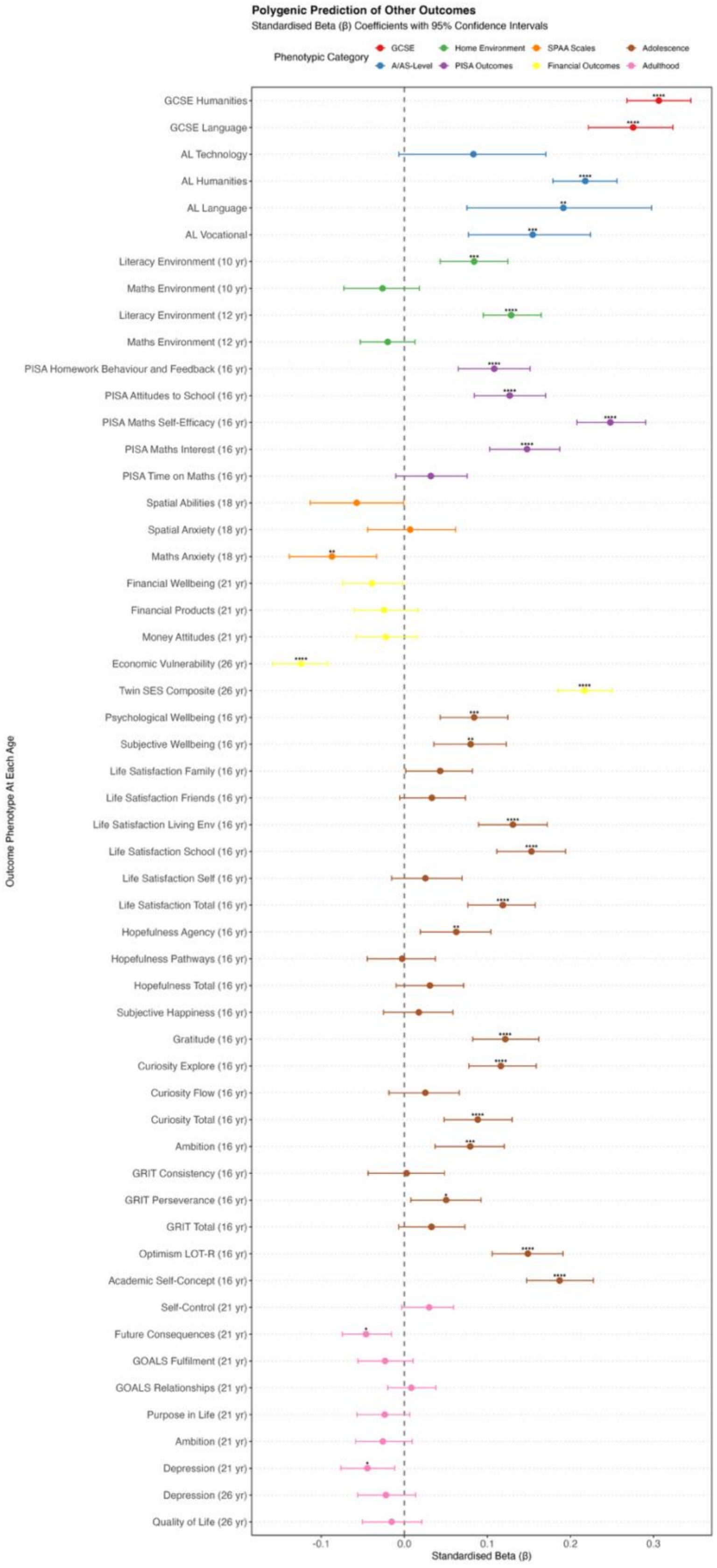

Figure C9. Polygenic prediction of common latent factors across age and raters

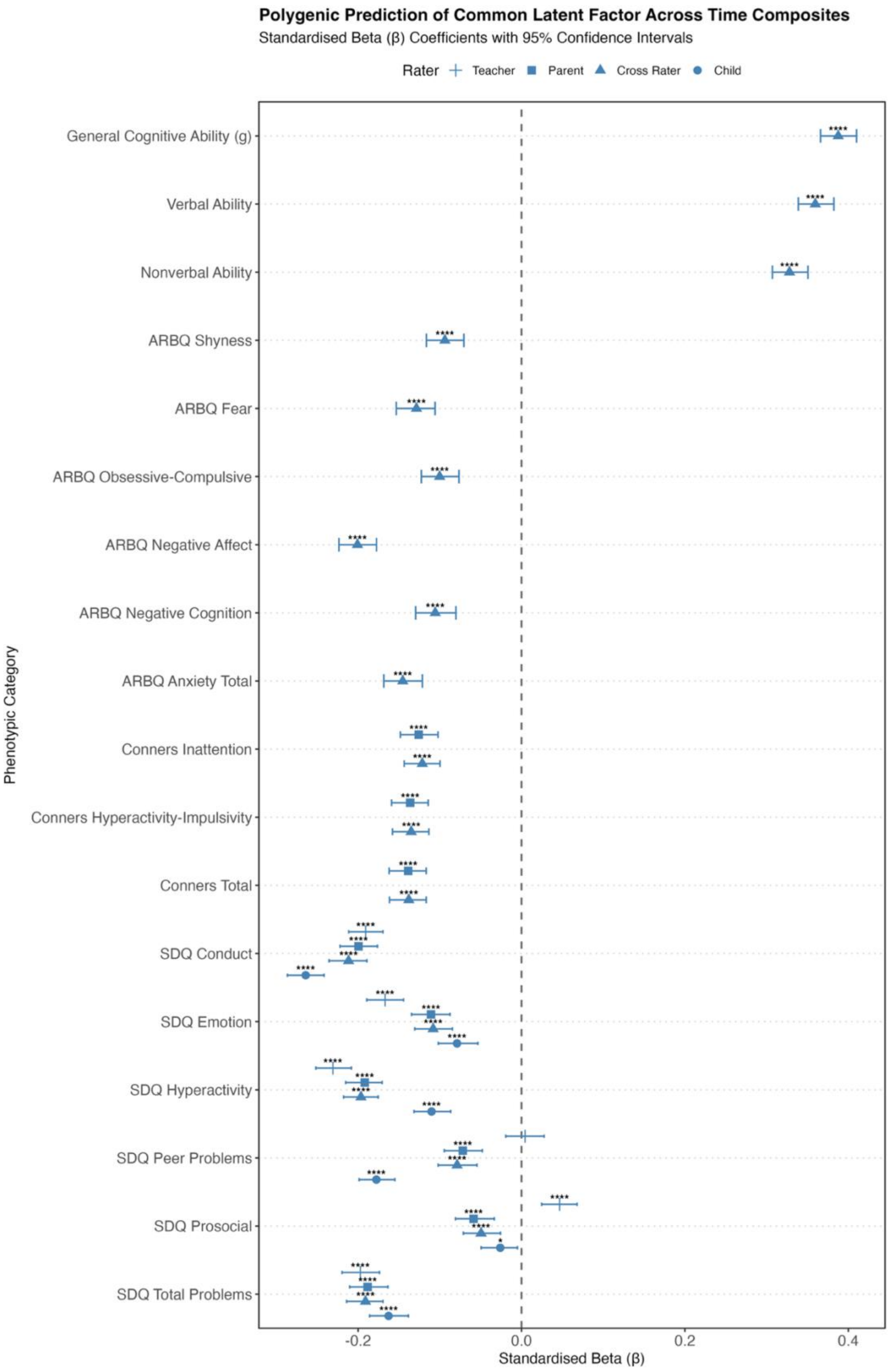

Figure C10. Confirmatory factor analysis (CFA) path diagrams (provided in a separate PDF file)

All 39 CFAs are compiled in a single PDF file ("AppendixC_SupFigureC10_CFAplots.pdf"), with models labelled C10-1 through C10-39. Path coefficients are standardised estimates. Models were fitted using confirmatory factor analysis in the *lavaan* package with full information maximum likelihood (FIML) estimation. The full twin sample was used with clustering by family specified to account for non-independence and increase statistical power. Complete numerical results are detailed in Tables B3 to B5.

All plots are constructed using the semPaths function from the *semPlot* package. Latent constructs are depicted as ovals, representing unobserved traits extracted from multiple observed indicators shown as rectangles. Observed variable labels specify the age of measurement and, for behavioural outcomes, the rater (e.g., yr7P for parent-rated phenotype at age 7, yr7T for teacher-rated). When multiple raters are used, the rater is specified in the label (P = Parent, T = Teacher, C = Child/Self); otherwise, a single rater (typically the child) is used across time, or non-overlapping raters are used developmentally (typically parent at younger ages, child at older ages). The observed polygenic g score (PGgS) appears as a rectangle at the top, modelled as a predictor of the latent factor.

All coefficients are standardised. Arrows from the latent factor (oval) to observed variables (rectangles) represent factor loadings (Table B4), indicating the strength of association between each measure and the underlying latent construct. The dotted line on the path to one indicator is a scaling artefact (this variable serves as the reference indicator) and does not affect interpretation of the standardised loading.

The path from PGgS to the latent factor shows the standardised regression coefficient (β), quantifying the direct predictive association between the polygenic score and the latent trait (Table B5). This coefficient is estimated within the CFA framework, where the measurement model and structural paths are fitted simultaneously. This approach differs from a two-stage analysis where factor scores are first extracted and then used in a separate regression, as the CFA accounts for measurement uncertainty in the latent construct during estimation.

Residual variances are displayed for both latent and observed variables. The value on the arrow on the left pointing to the latent factor represents its standardised residual variance (the proportion unexplained by PGgS), calculated as $1 - \beta^2$. Values on arrows pointing to observed variables on the bottom represent their standardised residual variances (the proportion unexplained by the latent factor), calculated as $1 - (\text{loading})^2$.

Small triangles represent the constant (1) used in the model specification to estimate intercepts (means) of variables. These are standard CFA notation and indicate that means are being modelled.

Path colours denote the direction of associations, with green indicating positive associations and red indicating negative associations.

The following list provides an overview of Figures C10-1 to C10-39, confirmatory factor analysis for:

C10-1. General Cognitive Ability (g) − Cross Rater

C10-2. Verbal Ability − Cross Rater

C10-3. Nonverbal Ability − Cross Rater

C10-4. ARBQ Shyness − Cross Rater

C10-5. ARBQ Fear − Cross Rater

C10-6. ARBQ Obsessive-Compulsive − Cross Rater

C10-7. ARBQ Negative Affect − Cross Rater

C10-8. ARBQ Negative Cognition − Cross Rater

C10-9. ARBQ Anxiety Total − Cross Rater

C10-10. Conners Inattention − Cross Rater

C10-11. Conners Inattention − Parent Only

C10-12. Conners Hyperactivity-Impulsivity − Cross Rater

C10-13. Conners Hyperactivity-Impulsivity − Parent Only

C10-14. Conners Total − Cross Rater

C10-15. Conners Total − Parent Only

C10-16. SDQ Conduct − Parent Only

C10-17. SDQ Conduct − Teacher Only

C10-18. SDQ Conduct − Child Only

C10-19. SDQ Emotion − Parent Only

C10-20. SDQ Emotion − Teacher Only

C10-21. SDQ Emotion − Child Only

C10-22. SDQ Hyperactivity − Parent Only

C10-23. SDQ Hyperactivity − Teacher Only

C10-24. SDQ Hyperactivity − Child Only

C10-25. SDQ Peer Problems − Parent Only

C10-26. SDQ Peer Problems − Teacher Only

C10-27. SDQ Peer Problems − Child Only

C10-28. SDQ Prosocial − Parent Only

C10-29. SDQ Prosocial − Teacher Only

C10-30. SDQ Prosocial − Child Only

C10-31. SDQ Total Problems − Parent Only

C10-32. SDQ Total Problems − Teacher Only

C10-33. SDQ Total Problems − Child Only

C10-34. SDQ Conduct − Cross Rater

C10-35. SDQ Emotion − Cross Rater

C10-36. SDQ Hyperactivity − Cross Rater

C10-37. SDQ Peer Problems − Cross Rater

C10-38. SDQ Prosocial − Cross Rater

C10-39. SDQ Total Problems − Cross Rater

Figure C11. Mean developmental trajectories for behavioural and anthropometric outcomes in individuals with high and low polygenic g scores

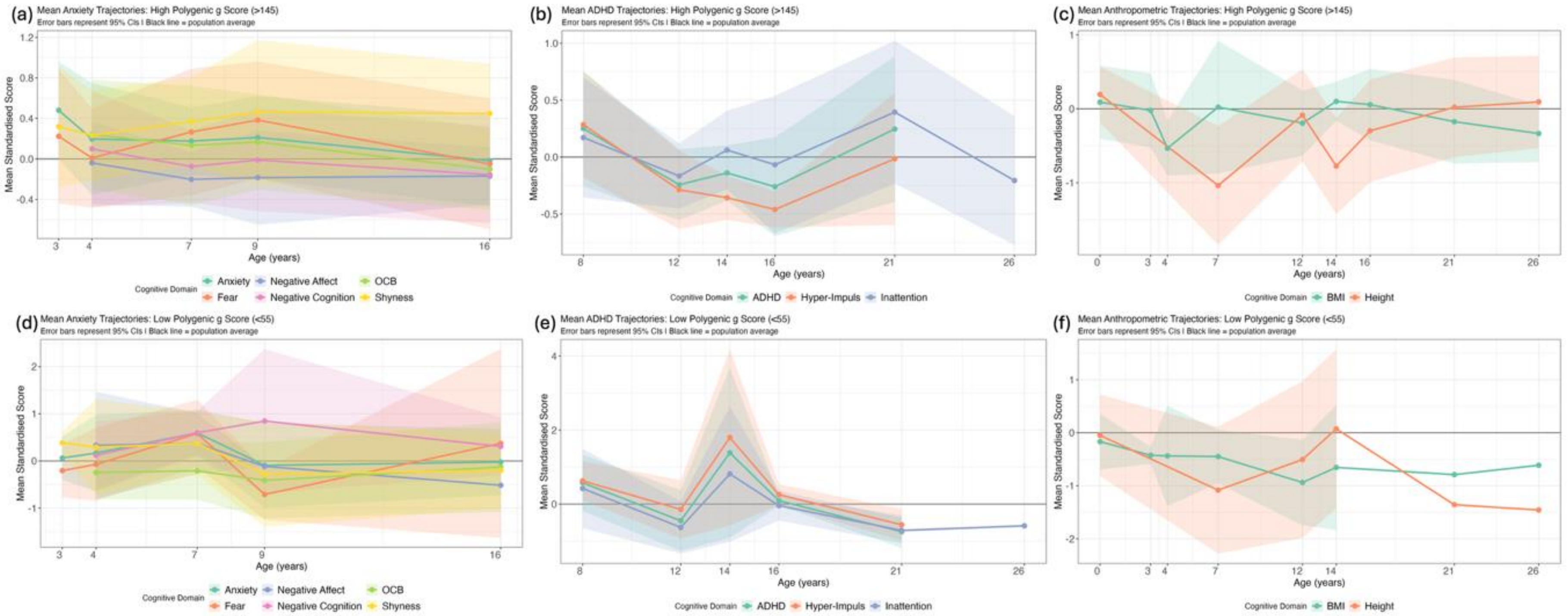

Note. Mean trajectories across development for individuals with polygenic g scores >145 (panels a-c) and <55 (panels d-f). Panel a/d: Anxiety-Related Behaviours Questionnaire (ARBQ) five subscales and total score. Panel b/e: Conners' Rating Scale ADHD two subscales (inattention and hyperactivity/impulsivity) and total score. Panel c/f: Height and body mass index (BMI). All measures are standardised to sample mean = 0, SD = 1. Shaded areas represent 95% confidence intervals; absence of confidence intervals indicates only one individual was measured at that age. For phenotypes with multiple raters, one rater per age is used: parent ratings before age 18 and child/self-ratings at age 18 and older. Black horizontal line at y = 0 represents the population average.

Figure C12. Individual developmental profiles for individuals with high polygenic g scores (>145)

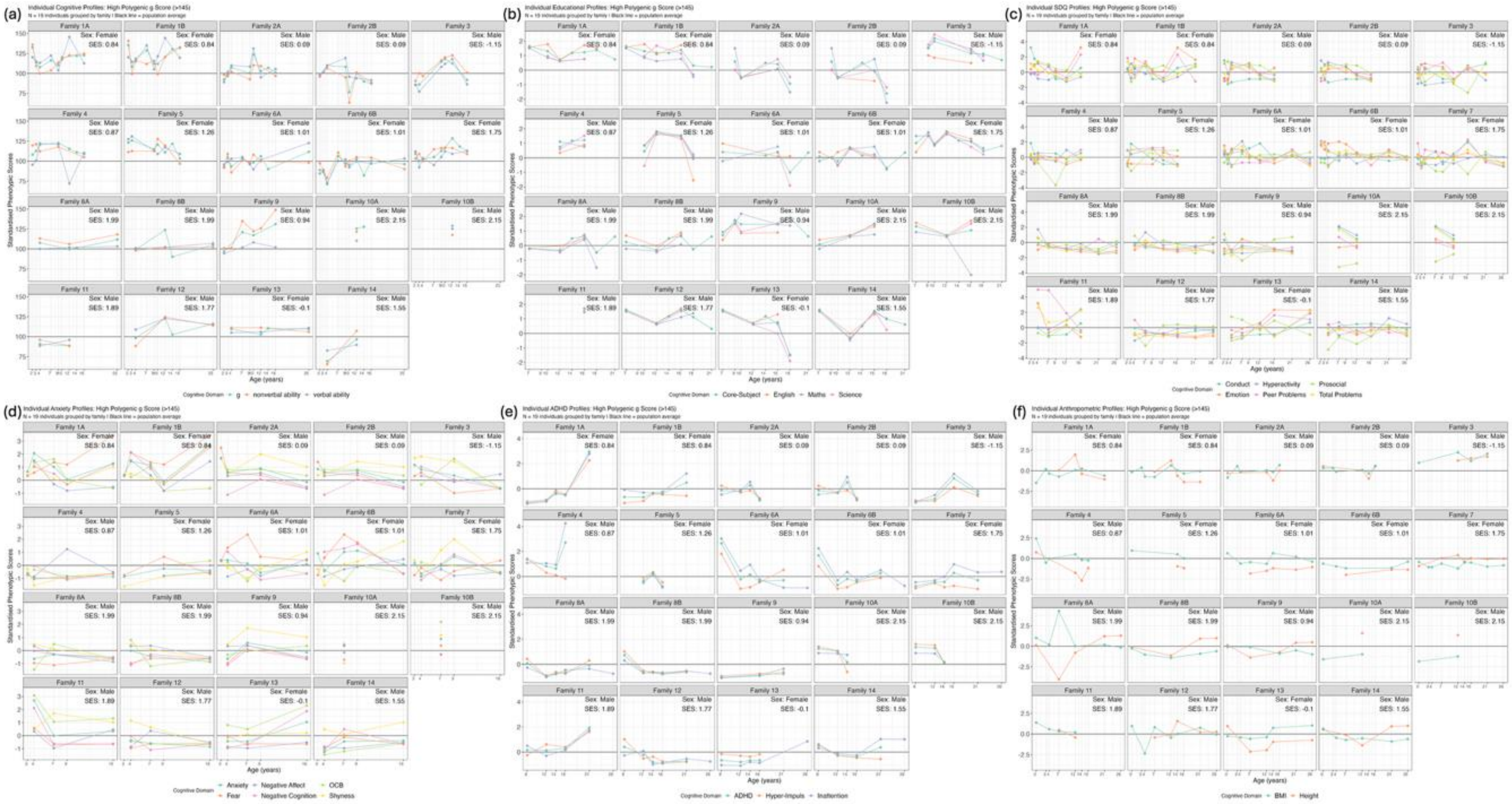

Note. Individual trajectories across development for all individuals with polygenic g scores >145. Each facet represents one individual, with family membership indicated in facet labels (e.g., Family 1A and Family 1B denote twins from the same family). Sex

and socioeconomic status (SES) are displayed within each facet. SES is standardised to mean = 0, SD = 1, using the earliest available measurement (birth, or if unavailable, age 7, 16, or 21 in that order). Panel a: General cognitive ability (g) composite and domain-specific composites (nonverbal and verbal ability). Panel b: Educational achievement outcomes including English, mathematics, science, and core-subject composite (sum of English, mathematics, and science). Panel c: Strengths and Difficulties Questionnaire (SDQ) five subscales and total problem score. Panel d: Anxiety-Related Behaviours Questionnaire (ARBQ) five subscales and total score. Panel e: Conners' Rating Scale ADHD two subscales (inattention and hyperactivity/impulsivity) and total score. Panel f: Height and body mass index (BMI). All measures are standardised to sample mean = 0, SD = 1, except for cognitive ability measures (mean = 100, SD = 15). For phenotypes with multiple raters, one rater per age is used: parent ratings before age 18 and child/self-ratings at age 18 and older. Black horizontal line at y = 0 represents the population average.

Figure C13. Individual developmental profiles for individuals with low polygenic g scores (<55)

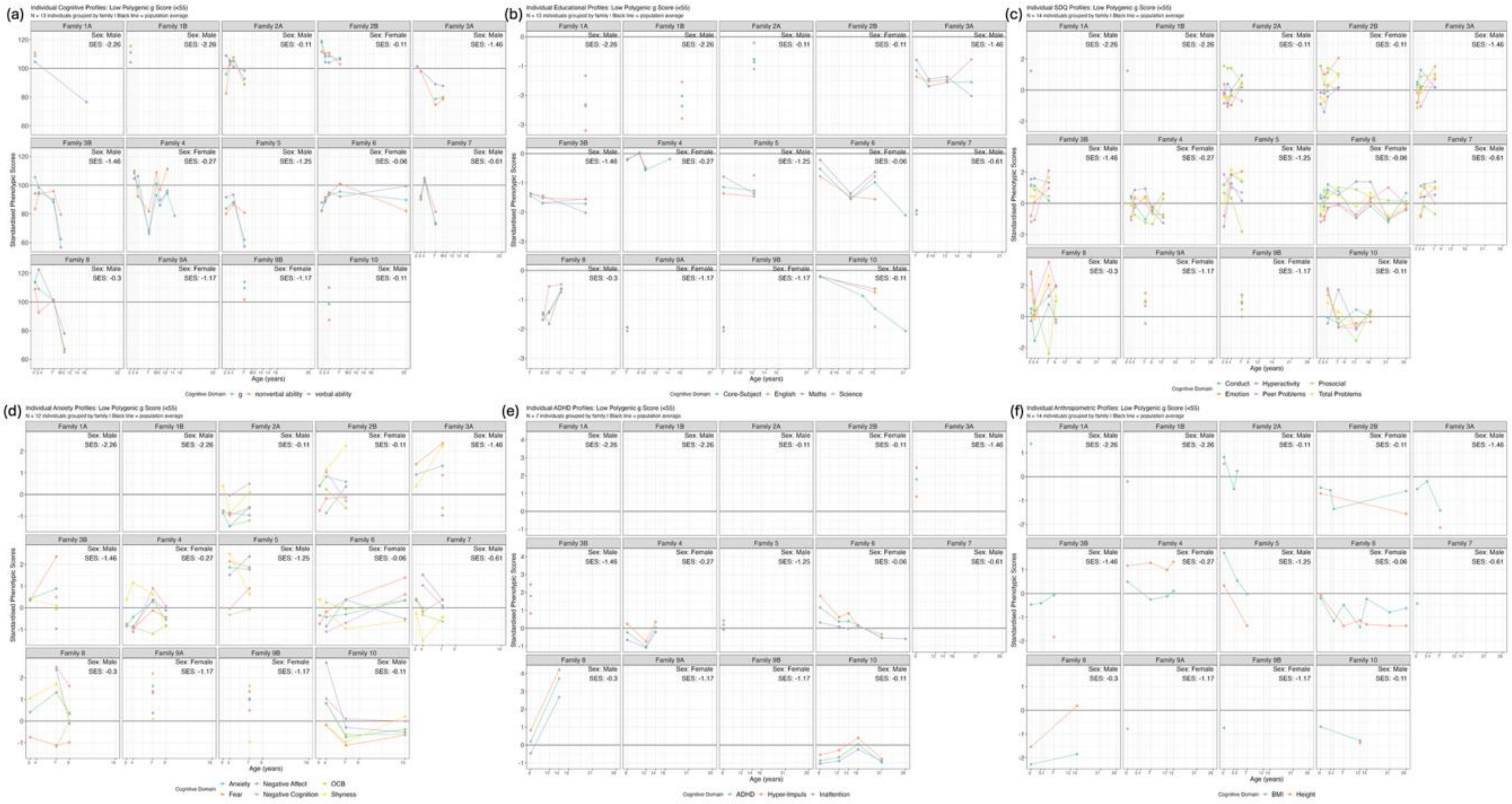

Note. Individual trajectories across development for all individuals with polygenic g scores <55. Each facet represents one individual, with family membership indicated in facet labels (e.g., Family 1A and Family 1B denote twins from the same family). Sex

and socioeconomic status (SES) are displayed within each facet. SES is standardised to sample mean = 0, SD = 1, using the earliest available measurement (birth, or if unavailable, age 7, 16, or 21 in that order). Panel a: General cognitive ability (g) composite and domain-specific composites (nonverbal and verbal ability). Panel b: educational achievement outcomes including English, mathematics, science, and core-subject composite (sum of English, mathematics, and science). Panel c: Strengths and Difficulties Questionnaire (SDQ) five subscales and total problem score. Panel d: Anxiety-Related Behaviours Questionnaire (ARBQ) five subscales and total score. Panel e: Conners' Rating Scale ADHD two subscales (inattention and hyperactivity/impulsivity) and total score. Panel f: Height and body mass index (BMI). All measures are standardised to sample mean = 0, SD = 1, except for cognitive ability measures (mean = 100, SD = 15). For phenotypes with multiple raters, one rater per age is used: parent ratings before age 18 and child/self-ratings at age 18 and older. Black horizontal line at y = 0 represents the population average.

References

Angold, A., Costello, E. J., Messer, S. C., & Pickles, A. (1995). Development of a short questionnaire for use in epidemiological studies of depression in children and adolescents. *International Journal of Methods in Psychiatric Research*, *5*(4), 237–249.

Burden, R. (1998). Assessing children’s perceptions of themselves as learners and problem-solvers: The construction of the Myself-as-Learner Scale (MALS). *School Psychology International*, *19*(4), 291–305. https://doi.org/10.1177/0143034398194002

Conners, C. K. (2003). *Conners’ Rating Scales-Revised: Technical Manual*. New York: Multi-Health System Inc.

Conners, C. K. (2008). *Conners 3rd Edition (Conners 3) Manual*. New York: MHS Assessments.

Crumbaugh, J. C., & Maholick, L. T. (1964). An experimental study in existentialism: The psychometric approach to Frankl’s concept of noogenic neurosis. *Journal of Clinical Psychology*, *20*(2), 200–207. https://doi.org/10.1002/1097-4679(196404)20:2%253C200::AID-JCLP2270200203%253E3.0.CO;2-U

Duckworth, A. L., Peterson, C., Matthews, M. D., & Kelly, D. R. (2007). Grit: Perseverance and passion for long-term goals. *Journal of Personality and Social Psychology*, *92*(6), 1087–1101. https://doi.org/10.1037/0022-3514.92.6.1087

Duckworth, A. L., & Quinn, P. D. (2009). Development and validation of the Short Grit Scale (GRIT–S). *Journal of Personality Assessment*, *91*(2), 166–174. https://doi.org/10.1080/00223890802634290

Eley, T. C., Bolton, D., O’Connor, T. G., Perrin, S., Smith, P., & Plomin, R. (2003). A twin study of anxiety-related behaviours in pre-school children. *Journal of Child Psychology and Psychiatry*, *44*(7), 945–960. https://doi.org/10.1111/1469-7610.00179

Hallett, V., Ronald, A., Rijsdijk, F., & Eley, T. C. (2009). Phenotypic and genetic differentiation of anxiety-related behaviors in middle childhood. *Depression and Anxiety*, *26*(4), 316–324. https://doi.org/10.1002/da.20539

Hopko, D. R., Mahadevan, R., Bare, R. L., & Hunt, M. K. (2003). The Abbreviated Math Anxiety Scale (AMAS): Construction, Validity, and Reliability. *Assessment*, *10*(2), 178–182. https://doi.org/10.1177/1073191103010002008

Huebner, E. S. (1994). Preliminary development and validation of a multidimensional life satisfaction scale for children. *Psychological Assessment*, *6*(2), 149–158. https://doi.org/10.1037/1040-3590.6.2.149

Kashdan, T. B., Rose, P., & Fincham, F. D. (2004). Curiosity and Exploration: Facilitating Positive Subjective Experiences and Personal Growth Opportunities. *Journal of Personality Assessment*, *82*(3), 291–305. https://doi.org/10.1207/s15327752jpa8203_05

Lavallee, L. F., Hatch, P. M., Michalos, A. C., & McKinley, T. (2007). Development of the Contentment with Life Assessment Scale (CLAS): Using Daily Life Experiences to Verify Levels of Self-Reported Life Satisfaction. *Social Indicators Research*, *83*(2), 201–244. https://doi.org/10.1007/s11205-006-9054-6

Lawton, C. A. (1994). Gender differences in way-finding strategies: Relationship to spatial ability and spatial anxiety. *Sex Roles: A Journal of Research*, *30*(11–12), 765–779. https://doi.org/10.1007/BF01544230

Lyubomirsky, S., & Lepper, H. S. (1999). A measure of subjective happiness: Preliminary reliability and construct validation. *Social Indicators Research*, *46*(2), 137–155. https://doi.org/10.1023/A:1006824100041

McCullough, M. E., Emmons, R. A., & Tsang, J.-A. (2002). The grateful disposition: A conceptual and empirical topography. *Journal of Personality and Social Psychology*, *82*(1), 112–127. https://doi.org/10.1037/0022-3514.82.1.112

Pöhlmann, K., & Brunstein, J. C. (1997). GOALS: Ein Fragebogen zur Messung von Lebenszielen. [GOALS: A questionnaire for assessing life goals.]. *Diagnostica*, *43*(1), 63–79.

Scheier, M. F., Carver, C. S., & Bridges, M. W. (1994). Distinguishing optimism from neuroticism (and trait anxiety, self-mastery, and self-esteem): A reevaluation of the Life Orientation Test. *Journal of Personality and Social Psychology*, *67*(6), 1063–1078. https://doi.org/10.1037/0022-3514.67.6.1063

Snyder, C. R., Hoza, B., Pelham, W. E., Rapoff, M., Ware, L., Danovsky, M., Highberger, L., Rubinstein, H., & Stahl, K. J. (1997). The development and validation of the Children's Hope Scale. *Journal of Pediatric Psychology*, *22*(3), 399–421. https://doi.org/10.1093/jpepsy/22.3.399

Strathman, A., Gleicher, F., Boninger, D. S., & Edwards, C. S. (1994). The consideration of future consequences: Weighing immediate and distant outcomes of behavior. *Journal of Personality and Social Psychology*, *66*(4), 742–752. https://doi.org/10.1037/0022-3514.66.4.742

Tangney, J. P., Baumeister, R. F., & Boone, A. L. (2004). High Self-Control Predicts Good Adjustment, Less Pathology, Better Grades, and Interpersonal Success. *Journal of Personality*, *72*(2), 271–324. https://doi.org/10.1111/j.0022-3506.2004.00263.x

Trzaskowski, M., Zavos, H. M. S., Haworth, C. M. A., Plomin, R., & Eley, T. C. (2012). Stable Genetic Influence on Anxiety-Related Behaviours Across Middle Childhood. *Journal of Abnormal Child Psychology*, *40*(1), 85–94. https://doi.org/10.1007/s10802-011-9545-z
