# Supplementary material for: Charting the cognitive development of children using adult ‘polygenic g scores’
Source: bioRxiv. 2025 Dec 23:2025.12.19.695378. Preprint. [Version 1] doi: 10.64898/2025.12.19.695378 (PMC12767516; doi:10.64898/2025.12.19.695378)

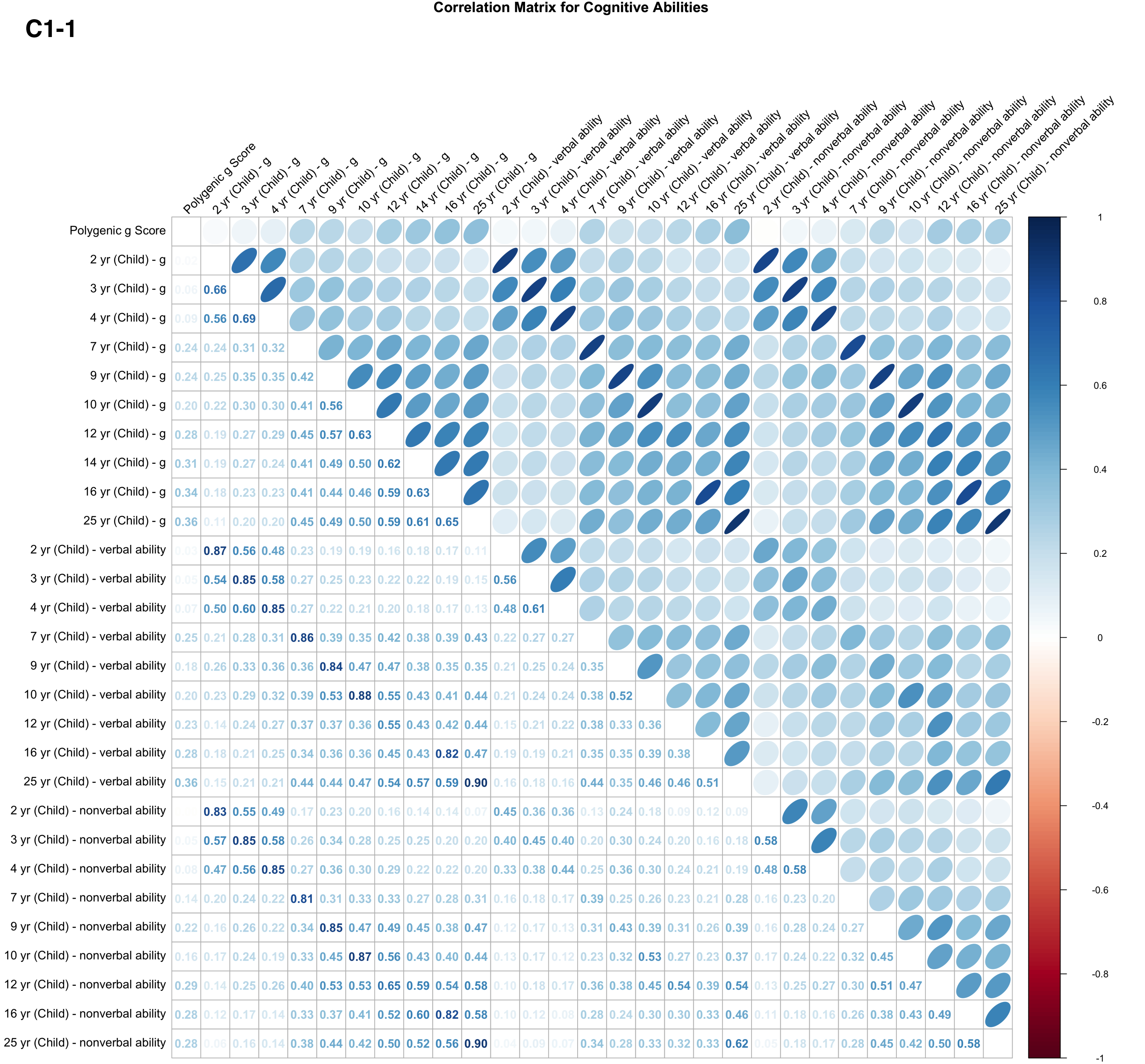
C1-1
Correlation Matrix for Cognitive Abilities
Polygenic g Score
2 yr (Child) - g
3 yr (Child) - g
4 yr (Child) - g
7 yr (Child) - g
9 yr (Child) - g
10 yr (Child) - g
12 yr (Child) - g
14 yr (Child) - g
16 yr (Child) - g
25 yr (Child) - g
2 yr (Child) - verbal ability
3 yr (Child) - verbal ability
4 yr (Child) - verbal ability
7 yr (Child) - verbal ability
9 yr (Child) - verbal ability
10 yr (Child) - verbal ability
12 yr (Child) - verbal ability
16 yr (Child) - verbal ability
25 yr (Child) - verbal ability
2 yr (Child) - nonverbal ability
3 yr (Child) - nonverbal ability
4 yr (Child) - nonverbal ability
7 yr (Child) - nonverbal ability
9 yr (Child) - nonverbal ability
10 yr (Child) - nonverbal ability
12 yr (Child) - nonverbal ability
16 yr (Child) - nonverbal ability
25 yr (Child) - nonverbal ability
1
0.8
0.6
0.4
0.2
0
-0.2
-0.4
-0.6
-0.8
-1
2 yr (Child) - g 0.02
3 yr (Child) - g 0.06 0.66
4 yr (Child) - g 0.09 0.56 0.69
7 yr (Child) - g 0.24 0.24 0.31 0.32
9 yr (Child) - g 0.24 0.25 0.35 0.35 0.42
10 yr (Child) - g 0.20 0.22 0.30 0.30 0.41 0.56
12 yr (Child) - g 0.28 0.19 0.27 0.29 0.45 0.57 0.63
14 yr (Child) - g 0.31 0.19 0.27 0.24 0.41 0.49 0.50 0.62
16 yr (Child) - g 0.34 0.18 0.23 0.23 0.41 0.44 0.46 0.59 0.63
25 yr (Child) - g 0.36 0.11 0.20 0.20 0.45 0.49 0.50 0.59 0.61 0.65
2 yr (Child) - verbal ability 0.03 0.87 0.56 0.48 0.23 0.19 0.19 0.16 0.18 0.17 0.11
3 yr (Child) - verbal ability 0.05 0.54 0.85 0.58 0.27 0.25 0.23 0.22 0.22 0.19 0.15 0.56
4 yr (Child) - verbal ability 0.07 0.50 0.60 0.85 0.27 0.22 0.21 0.20 0.18 0.17 0.13 0.48 0.61
7 yr (Child) - verbal ability 0.25 0.21 0.28 0.31 0.86 0.39 0.35 0.42 0.38 0.39 0.43 0.22 0.27 0.27
9 yr (Child) - verbal ability 0.18 0.26 0.33 0.36 0.36 0.84 0.47 0.47 0.38 0.35 0.35 0.21 0.25 0.24 0.35
10 yr (Child) - verbal ability 0.20 0.23 0.29 0.32 0.39 0.53 0.88 0.55 0.43 0.41 0.44 0.21 0.24 0.24 0.38 0.52
12 yr (Child) - verbal ability 0.23 0.14 0.24 0.27 0.37 0.37 0.36 0.55 0.43 0.42 0.44 0.15 0.21 0.22 0.38 0.33 0.36
16 yr (Child) - verbal ability 0.28 0.18 0.21 0.25 0.34 0.36 0.36 0.45 0.43 0.82 0.47 0.19 0.19 0.21 0.35 0.35 0.39 0.38
25 yr (Child) - verbal ability 0.36 0.15 0.21 0.21 0.44 0.44 0.47 0.54 0.57 0.59 0.90 0.16 0.18 0.16 0.44 0.35 0.46 0.46 0.51
2 yr (Child) - nonverbal ability 0.83 0.55 0.49 0.17 0.23 0.20 0.16 0.14 0.14 0.07 0.45 0.36 0.36 0.13 0.24 0.18 0.09 0.12 0.09
3 yr (Child) - nonverbal ability 0.05 0.57 0.85 0.58 0.26 0.34 0.28 0.25 0.25 0.20 0.20 0.40 0.45 0.40 0.20 0.30 0.24 0.20 0.16 0.18 0.58
4 yr (Child) - nonverbal ability 0.08 0.47 0.56 0.85 0.27 0.36 0.30 0.29 0.22 0.22 0.20 0.33 0.38 0.44 0.25 0.36 0.30 0.24 0.21 0.19 0.48 0.58
7 yr (Child) - nonverbal ability 0.14 0.20 0.24 0.22 0.81 0.31 0.33 0.33 0.27 0.28 0.31 0.16 0.18 0.17 0.39 0.25 0.26 0.23 0.21 0.28 0.16 0.23 0.20
9 yr (Child) - nonverbal ability 0.22 0.16 0.26 0.22 0.34 0.85 0.47 0.49 0.45 0.38 0.47 0.12 0.17 0.13 0.31 0.43 0.39 0.31 0.26 0.39 0.16 0.28 0.24 0.27
10 yr (Child) - nonverbal ability 0.16 0.17 0.24 0.19 0.33 0.45 0.87 0.56 0.43 0.40 0.44 0.13 0.17 0.12 0.23 0.32 0.53 0.27 0.23 0.37 0.17 0.24 0.22 0.32 0.45
12 yr (Child) - nonverbal ability 0.29 0.14 0.25 0.26 0.40 0.53 0.53 0.65 0.59 0.54 0.58 0.10 0.18 0.17 0.36 0.38 0.45 0.54 0.39 0.54 0.13 0.25 0.27 0.30 0.51 0.47
16 yr (Child) - nonverbal ability 0.28 0.12 0.17 0.14 0.33 0.37 0.41 0.52 0.60 0.82 0.58 0.10 0.12 0.08 0.28 0.24 0.30 0.30 0.33 0.46 0.11 0.18 0.16 0.26 0.38 0.43 0.49
25 yr (Child) - nonverbal ability 0.28 0.06 0.16 0.14 0.38 0.44 0.42 0.50 0.52 0.56 0.90 0.04 0.09 0.07 0.34 0.28 0.33 0.32 0.33 0.62 0.05 0.18 0.17 0.28 0.45 0.42 0.50 0.58

C1-2

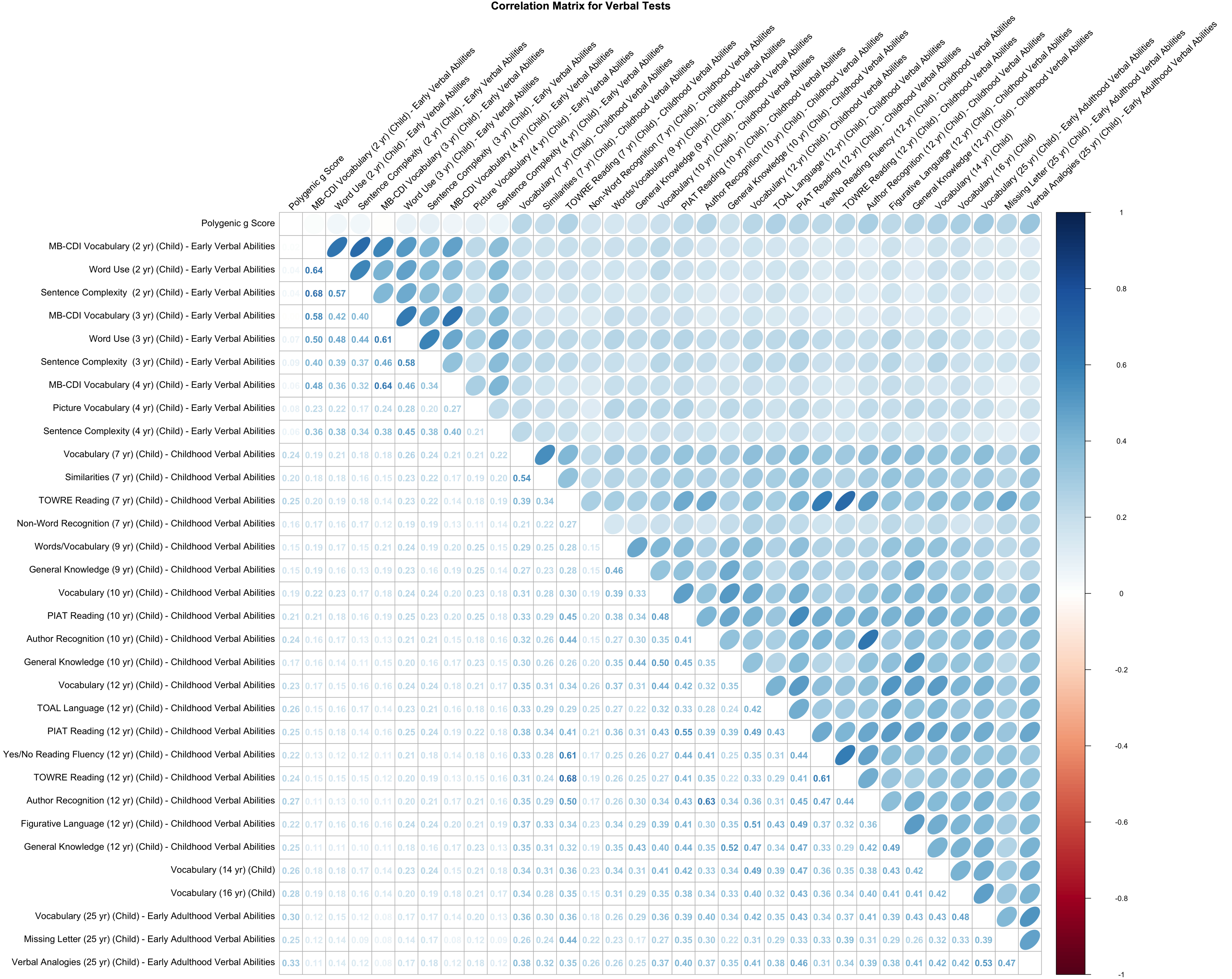
Correlation Matrix for Verbal Tests
Polygenic g Score
MB-CDI Vocabulary (2 yr) (Child) - Early Verbal Abilities
Word Use (2 yr) (Child) - Early Verbal Abilities
Sentence Complexity (2 yr) (Child) - Early Verbal Abilities
MB-CDI Vocabulary (3 yr) (Child) - Early Verbal Abilities
Word Use (3 yr) (Child) - Early Verbal Abilities
Sentence Complexity (3 yr) (Child) - Early Verbal Abilities
MB-CDI Vocabulary (4 yr) (Child) - Early Verbal Abilities
Picture Vocabulary (4 yr) (Child) - Early Verbal Abilities
Sentence Complexity (4 yr) (Child) - Early Verbal Abilities
Vocabulary (7 yr) (Child) - Childhood Verbal Abilities
Similarities (7 yr) (Child) - Childhood Verbal Abilities
TOWRE Reading (7 yr) (Child) - Childhood Verbal Abilities
Non-Word Recognition (7 yr) (Child) - Childhood Verbal Abilities
Words/Vocabulary (9 yr) (Child) - Childhood Verbal Abilities
General Knowledge (9 yr) (Child) - Childhood Verbal Abilities
Vocabulary (10 yr) (Child) - Childhood Verbal Abilities
PIAT Reading (10 yr) (Child) - Childhood Verbal Abilities
Author Recognition (10 yr) (Child) - Childhood Verbal Abilities
General Knowledge (10 yr) (Child) - Childhood Verbal Abilities
Vocabulary (12 yr) (Child) - Childhood Verbal Abilities
TOAL Language (12 yr) (Child) - Childhood Verbal Abilities
PIAT Reading (12 yr) (Child) - Childhood Verbal Abilities
Yes/No Reading Fluency (12 yr) (Child) - Childhood Verbal Abilities
TOWRE Reading (12 yr) (Child) - Childhood Verbal Abilities
Author Recognition (12 yr) (Child) - Childhood Verbal Abilities
Figurative Language (12 yr) (Child) - Childhood Verbal Abilities
General Knowledge (12 yr) (Child) - Childhood Verbal Abilities
Vocabulary (14 yr) (Child)
Vocabulary (16 yr) (Child)
Vocabulary (25 yr) (Child) - Early Adulthood Verbal Abilities
Missing Letter (25 yr) (Child) - Early Adulthood Verbal Abilities
Verbal Analogies (25 yr) (Child) - Early Adulthood Verbal Abilities
1
0.8
0.6
0.4
0.2
0
-0.2
-0.4
-0.6
-0.8
-1

C1-3

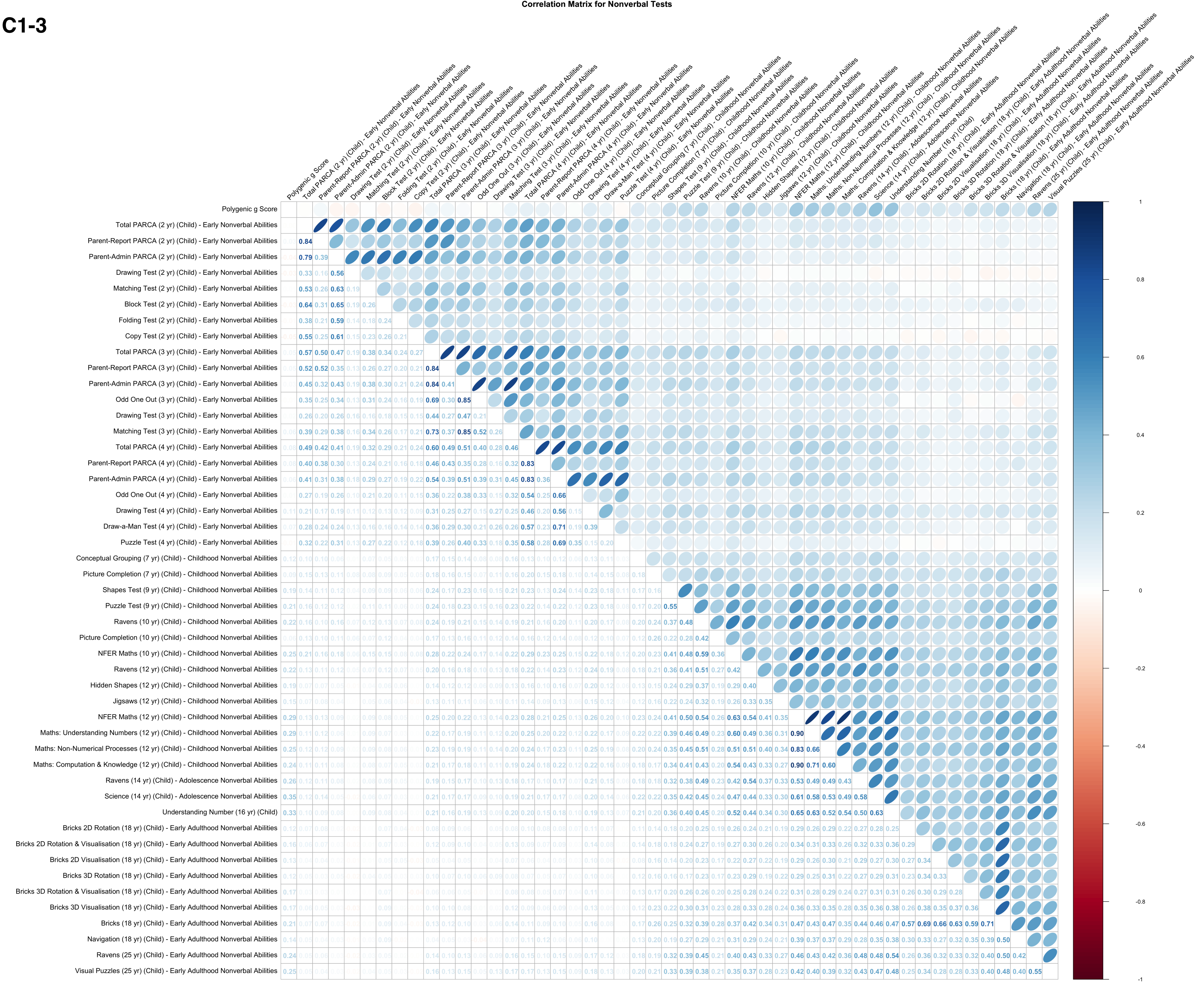
Correlation Matrix for Nonverbal Tests
Polygenic g Score
Total PARCA (2 yr) (Child) - Early Nonverbal Abilities
Parent-Report PARCA (2 yr) (Child) - Early Nonverbal Abilities
Parent-Admin PARCA (2 yr) (Child) - Early Nonverbal Abilities
Drawing Test (2 yr) (Child) - Early Nonverbal Abilities
Matching Test (2 yr) (Child) - Early Nonverbal Abilities
Block Test (2 yr) (Child) - Early Nonverbal Abilities
Folding Test (2 yr) (Child) - Early Nonverbal Abilities
Copy Test (2 yr) (Child) - Early Nonverbal Abilities
Total PARCA (3 yr) (Child) - Early Nonverbal Abilities
Parent-Report PARCA (3 yr) (Child) - Early Nonverbal Abilities
Parent-Admin PARCA (3 yr) (Child) - Early Nonverbal Abilities
Odd One Out (3 yr) (Child) - Early Nonverbal Abilities
Drawing Test (3 yr) (Child) - Early Nonverbal Abilities
Matching Test (3 yr) (Child) - Early Nonverbal Abilities
Total PARCA (4 yr) (Child) - Early Nonverbal Abilities
Parent-Report PARCA (4 yr) (Child) - Early Nonverbal Abilities
Parent-Admin PARCA (4 yr) (Child) - Early Nonverbal Abilities
Odd One Out (4 yr) (Child) - Early Nonverbal Abilities
Drawing Test (4 yr) (Child) - Early Nonverbal Abilities
Draw-a-Man Test (4 yr) (Child) - Early Nonverbal Abilities
Puzzle Test (4 yr) (Child) - Early Nonverbal Abilities
Conceptual Grouping (7 yr) (Child) - Childhood Nonverbal Abilities
Picture Completion (7 yr) (Child) - Childhood Nonverbal Abilities
Shapes Test (9 yr) (Child) - Childhood Nonverbal Abilities
Puzzle Test (9 yr) (Child) - Childhood Nonverbal Abilities
Ravens (10 yr) (Child) - Childhood Nonverbal Abilities
Picture Completion (10 yr) (Child) - Childhood Nonverbal Abilities
NFER Maths (10 yr) (Child) - Childhood Nonverbal Abilities
Ravens (12 yr) (Child) - Childhood Nonverbal Abilities
Hidden Shapes (12 yr) (Child) - Childhood Nonverbal Abilities
Jigsaws (12 yr) (Child) - Childhood Nonverbal Abilities
NFER Maths (12 yr) (Child) - Childhood Nonverbal Abilities
Maths: Understanding Numbers (12 yr) (Child) - Childhood Nonverbal Abilities
Maths: Non-Numerical Processes (12 yr) (Child) - Childhood Nonverbal Abilities
Maths: Computation & Knowledge (12 yr) (Child) - Childhood Nonverbal Abilities
Ravens (14 yr) (Child) - Adolescence Nonverbal Abilities
Science (14 yr) (Child) - Adolescence Nonverbal Abilities
Understanding Number (16 yr) (Child)
Bricks 2D Rotation (18 yr) (Child) - Early Adulthood Nonverbal Abilities
Bricks 2D Rotation & Visualisation (18 yr) (Child) - Early Adulthood Nonverbal Abilities
Bricks 2D Visualisation (18 yr) (Child) - Early Adulthood Nonverbal Abilities
Bricks 3D Rotation (18 yr) (Child) - Early Adulthood Nonverbal Abilities
Bricks 3D Rotation & Visualisation (18 yr) (Child) - Early Adulthood Nonverbal Abilities
Bricks 3D Visualisation (18 yr) (Child) - Early Adulthood Nonverbal Abilities
Bricks (18 yr) (Child) - Early Adulthood Nonverbal Abilities
Navigation (18 yr) (Child) - Early Adulthood Nonverbal Abilities
Ravens (25 yr) (Child) - Early Adulthood Nonverbal Abilities
Visual Puzzles (25 yr) (Child) - Early Adulthood Nonverbal Abilities
1
0.8
0.6
0.4
0.2
0
-0.2
-0.4
-0.6
-0.8
-1

C1-4

Correlation Matrix for Educational Achievement and Attainment

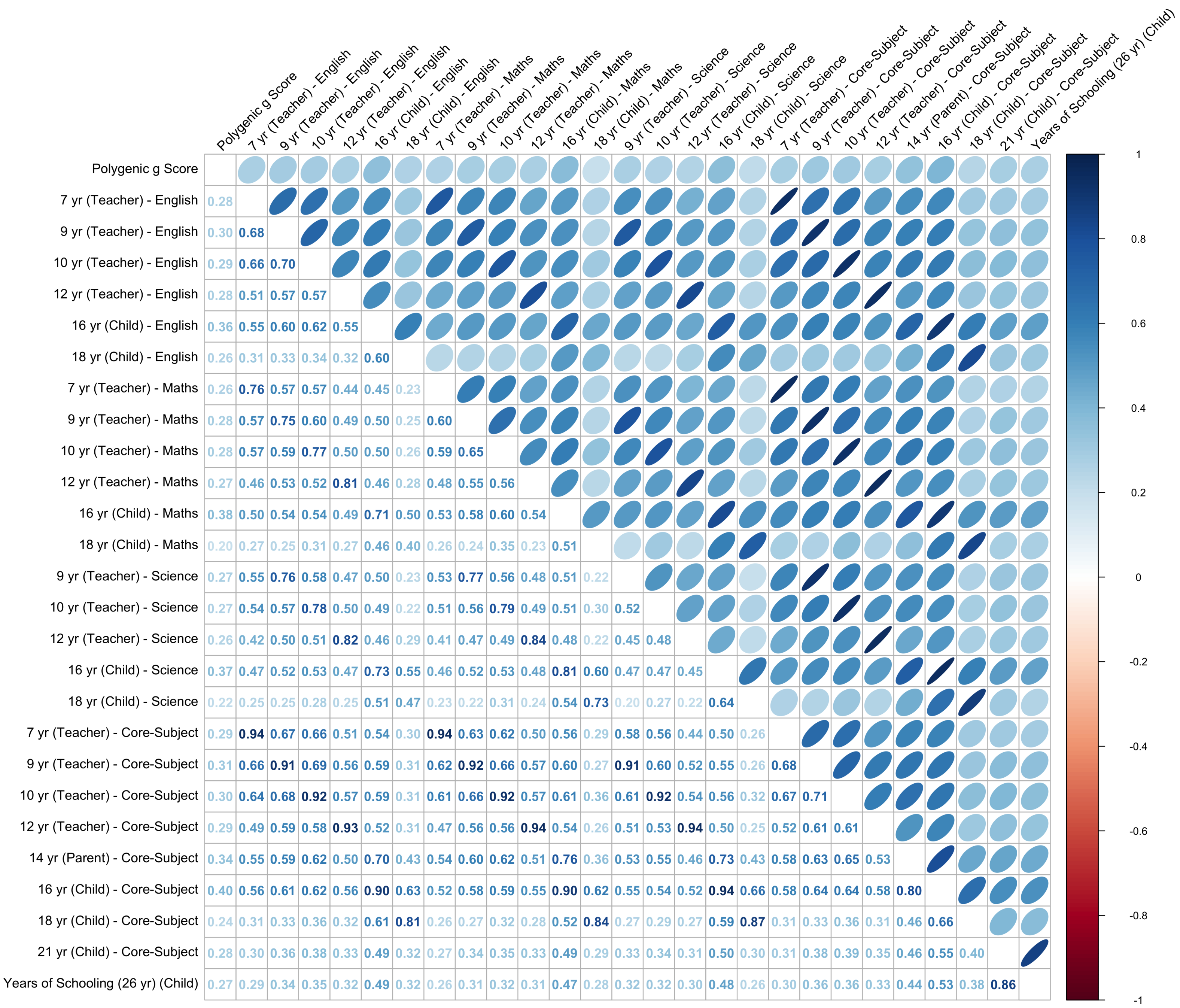

C1-5

Correlation Matrix for Strengths and Difficulties Questionnaire (SDQ)

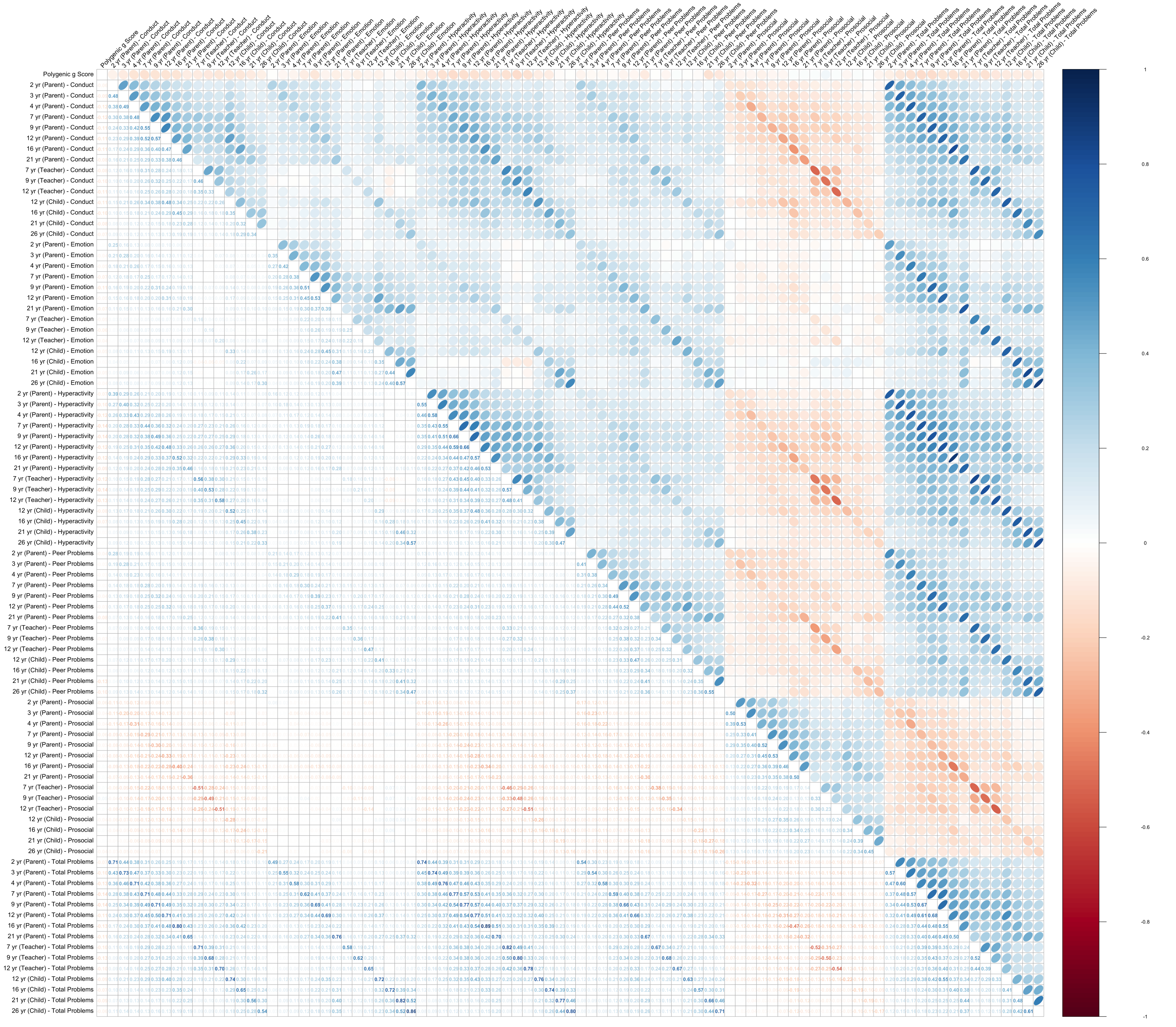

C1-6

Correlation Matrix for Anxiety Measures

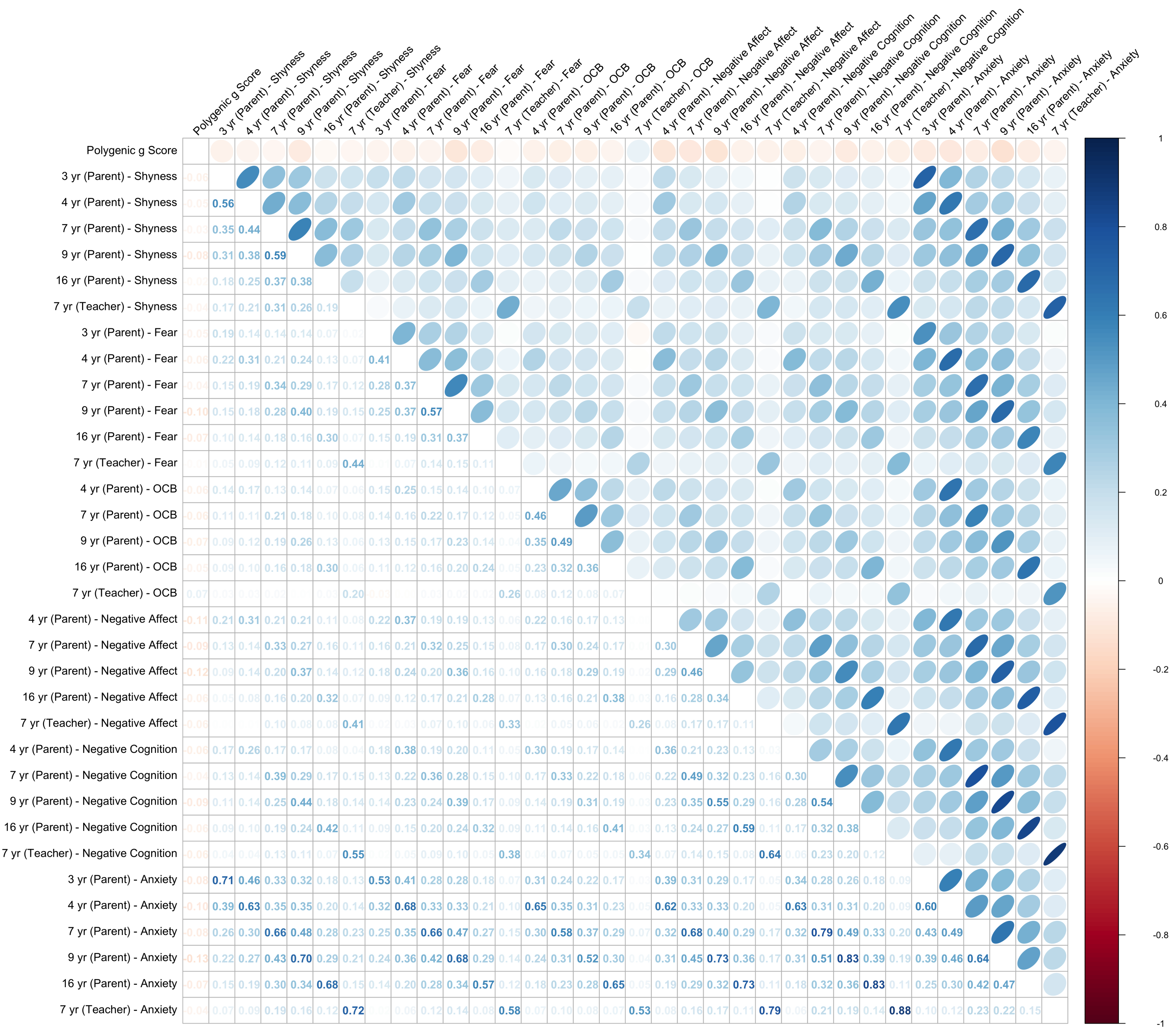

C1-7

Correlation Matrix for ADHD Measures

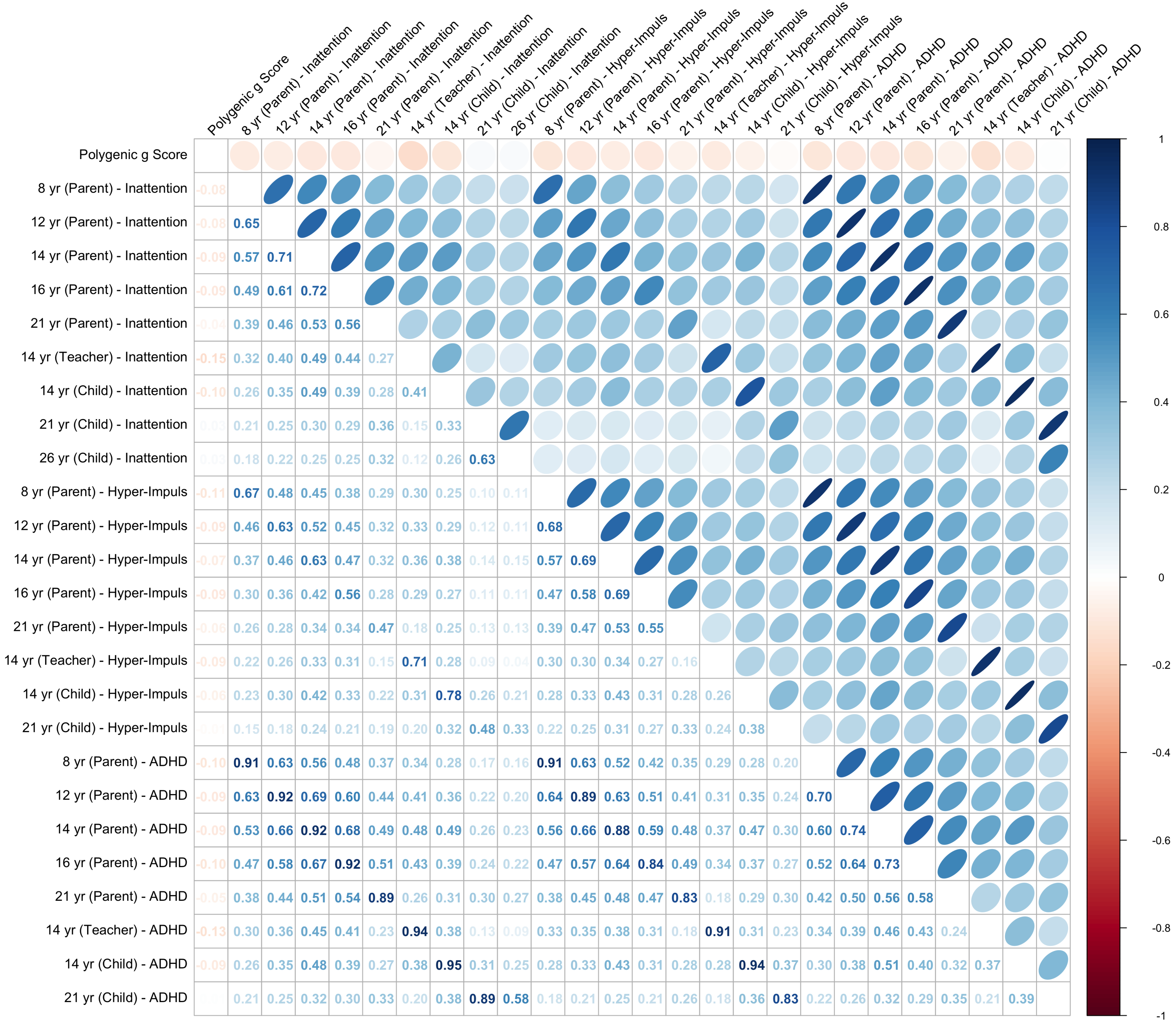

C1-8

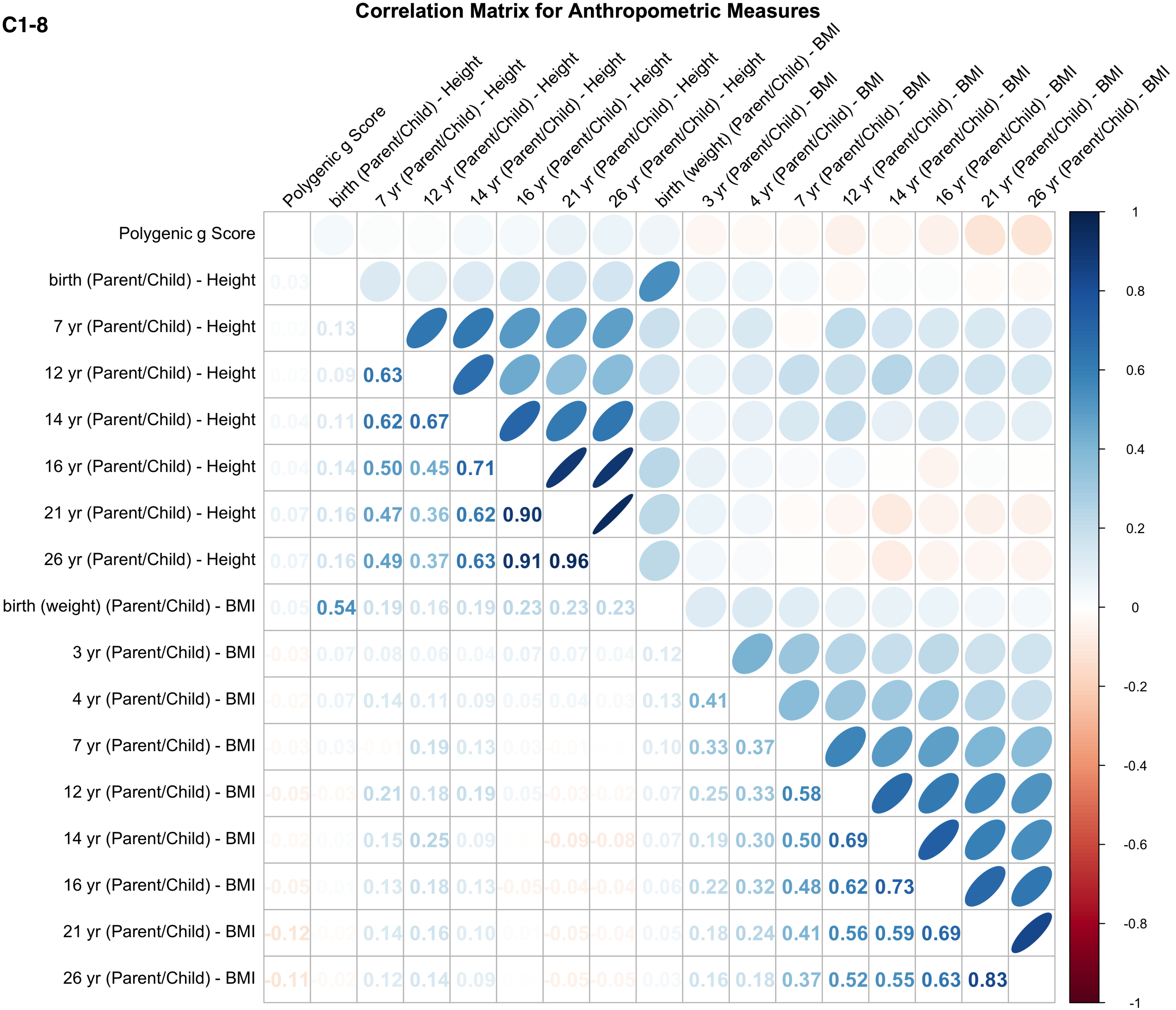
Correlation Matrix for Anthropometric Measures
Polygenic g Score
birth (Parent/Child) - Height
7 yr (Parent/Child) - Height
12 yr (Parent/Child) - Height
14 yr (Parent/Child) - Height
16 yr (Parent/Child) - Height
21 yr (Parent/Child) - Height
26 yr (Parent/Child) - Height
birth (weight) (Parent/Child) - BMI
3 yr (Parent/Child) - BMI
4 yr (Parent/Child) - BMI
7 yr (Parent/Child) - BMI
12 yr (Parent/Child) - BMI
14 yr (Parent/Child) - BMI
16 yr (Parent/Child) - BMI
21 yr (Parent/Child) - BMI
26 yr (Parent/Child) - BMI
0.03
0.13
0.09 0.63
0.11 0.62 0.67
0.14 0.50 0.45 0.71
0.07 0.16 0.47 0.36 0.62 0.90
0.07 0.16 0.49 0.37 0.63 0.91 0.96
0.05 0.54 0.19 0.16 0.19 0.23 0.23 0.23
-0.03 0.07 0.08 0.06 0.04 0.07 0.07 0.04 0.12
-0.02 0.07 0.14 0.11 0.09 0.05 0.04 0.03 0.13 0.41
-0.03 0.03 -0.01 0.19 0.13 0.03 -0.01 0.10 0.33 0.37
-0.05 -0.03 0.21 0.18 0.19 0.05 -0.03 -0.02 0.07 0.25 0.33 0.58
-0.02 0.15 0.25 0.09 -0.09 -0.08 0.07 0.19 0.30 0.50 0.69
-0.05 0.01 0.13 0.18 0.13 -0.05 -0.04 -0.04 0.06 0.22 0.32 0.48 0.62 0.73
-0.12 -0.02 0.14 0.16 0.10 0.01 -0.05 -0.04 0.05 0.18 0.24 0.41 0.56 0.59 0.69
-0.11 -0.02 0.12 0.14 0.09 -0.05 -0.05 0.03 0.16 0.18 0.37 0.52 0.55 0.63 0.83
1
0.8
0.6
0.4
0.2
0
-0.2
-0.4
-0.6
-0.8
-1

C1-9

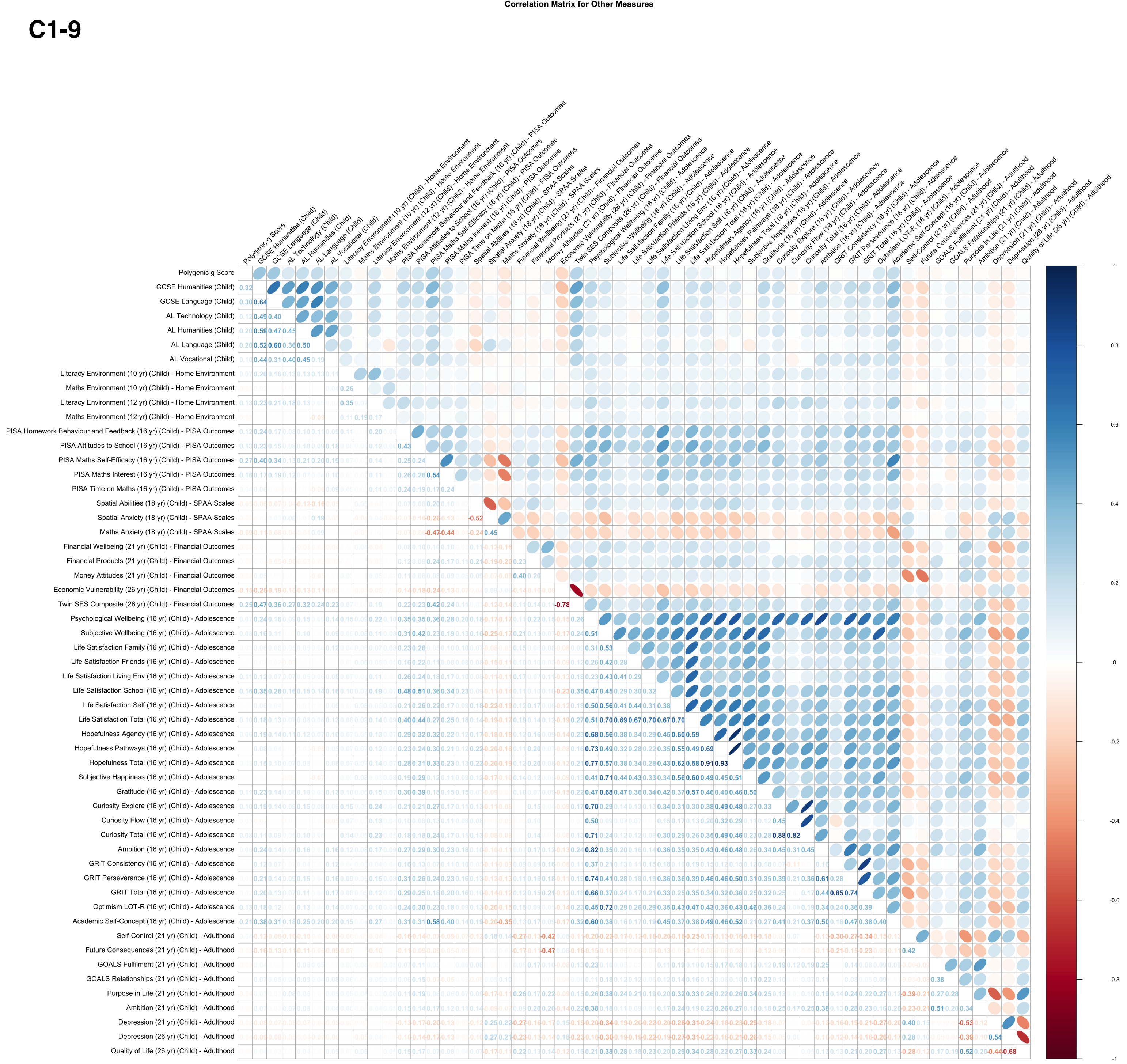
Correlation Matrix for Other Measures
Polygenic g Score
GCSE Humanities (Child)
GCSE Language (Child)
AL Technology (Child)
AL Humanities (Child)
AL Language (Child)
AL Vocational (Child)
Literacy Environment (10 yr) (Child) - Home Environment
Maths Environment (10 yr) (Child) - Home Environment
Literacy Environment (12 yr) (Child) - Home Environment
Maths Environment (12 yr) (Child) - Home Environment
PISA Homework Behaviour and Feedback (16 yr) (Child) - PISA Outcomes
PISA Attitudes to School (16 yr) (Child) - PISA Outcomes
PISA Maths Self-Efficacy (16 yr) (Child) - PISA Outcomes
PISA Maths Interest (16 yr) (Child) - PISA Outcomes
PISA Time on Maths (16 yr) (Child) - PISA Outcomes
Spatial Abilities (18 yr) (Child) - SPAA Scales
Spatial Anxiety (18 yr) (Child) - SPAA Scales
Maths Anxiety (18 yr) (Child) - SPAA Scales
Financial Wellbeing (21 yr) (Child) - Financial Outcomes
Financial Products (21 yr) (Child) - Financial Outcomes
Money Attitudes (21 yr) (Child) - Financial Outcomes
Economic Vulnerability (26 yr) (Child) - Financial Outcomes
Twin SES Composite (26 yr) (Child) - Financial Outcomes
Psychological Wellbeing (16 yr) (Child) - Adolescence
Subjective Wellbeing (16 yr) (Child) - Adolescence
Life Satisfaction Family (16 yr) (Child) - Adolescence
Life Satisfaction Friends (16 yr) (Child) - Adolescence
Life Satisfaction Living Env (16 yr) (Child) - Adolescence
Life Satisfaction School (16 yr) (Child) - Adolescence
Life Satisfaction Self (16 yr) (Child) - Adolescence
Life Satisfaction Total (16 yr) (Child) - Adolescence
Hopefulness Agency (16 yr) (Child) - Adolescence
Hopefulness Pathways (16 yr) (Child) - Adolescence
Hopefulness Total (16 yr) (Child) - Adolescence
Subjective Happiness (16 yr) (Child) - Adolescence
Gratitude (16 yr) (Child) - Adolescence
Curiosity Explore (16 yr) (Child) - Adolescence
Curiosity Flow (16 yr) (Child) - Adolescence
Curiosity Total (16 yr) (Child) - Adolescence
Ambition (16 yr) (Child) - Adolescence
GRIT Consistency (16 yr) (Child) - Adolescence
GRIT Perseverance (16 yr) (Child) - Adolescence
GRIT Total (16 yr) (Child) - Adolescence
Optimism LOT-R (16 yr) (Child) - Adolescence
Academic Self-Concept (16 yr) (Child) - Adolescence
Self-Control (21 yr) (Child) - Adulthood
Future Consequences (21 yr) (Child) - Adulthood
GOALS Fulfilment (21 yr) (Child) - Adulthood
GOALS Relationships (21 yr) (Child) - Adulthood
Purpose in Life (21 yr) (Child) - Adulthood
Ambition (21 yr) (Child) - Adulthood
Depression (21 yr) (Child) - Adulthood
Depression (26 yr) (Child) - Adulthood
Quality of Life (26 yr) (Child) - Adulthood
1
0.8
0.6
0.4
0.2
0
-0.2
-0.4
-0.6
-0.8
-1

C1-10

Correlation Matrix for Common Latent Factors Across Ages (and Raters)

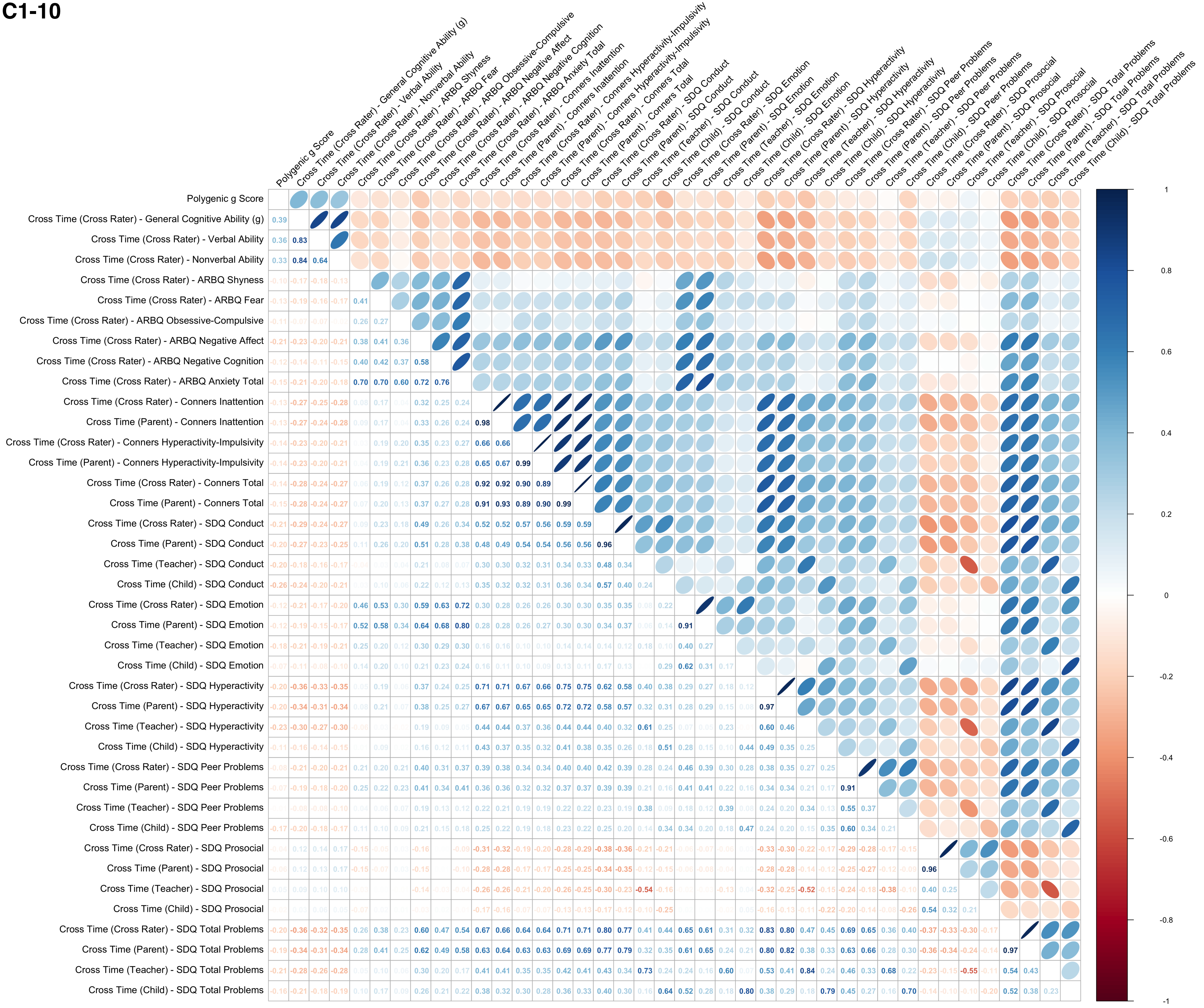

Supplement: Supplement 3 [file media-3.pdf]
