# Supplementary material for: Charting the cognitive development of children using adult ‘polygenic g scores’
Source: bioRxiv. 2025 Dec 23:2025.12.19.695378. Preprint. [Version 1] doi: 10.64898/2025.12.19.695378 (PMC12767516; doi:10.64898/2025.12.19.695378)

General Cognitive Ability (g) – Cross Rater

C10-1

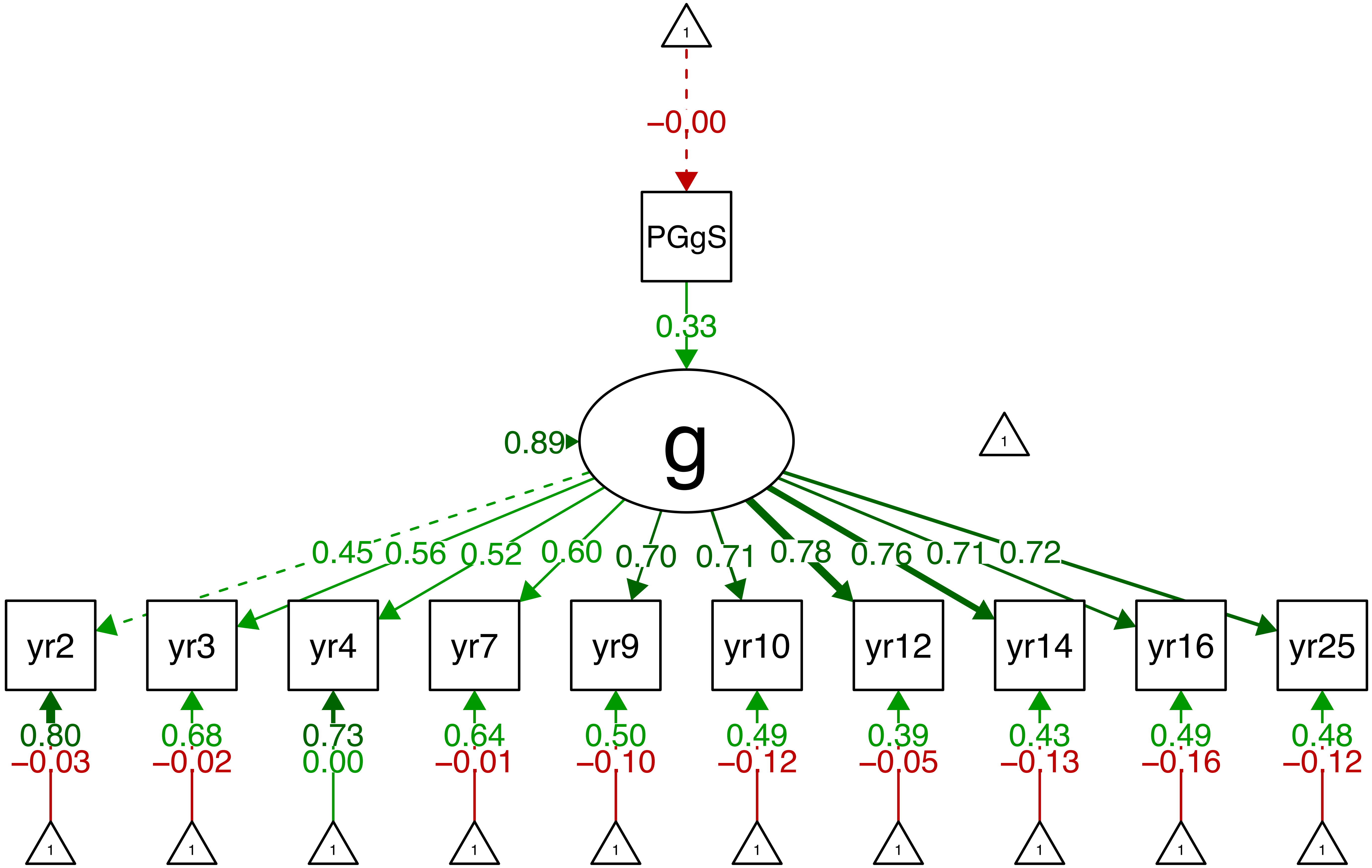

C10-2

Verbal Ability – Cross Rater

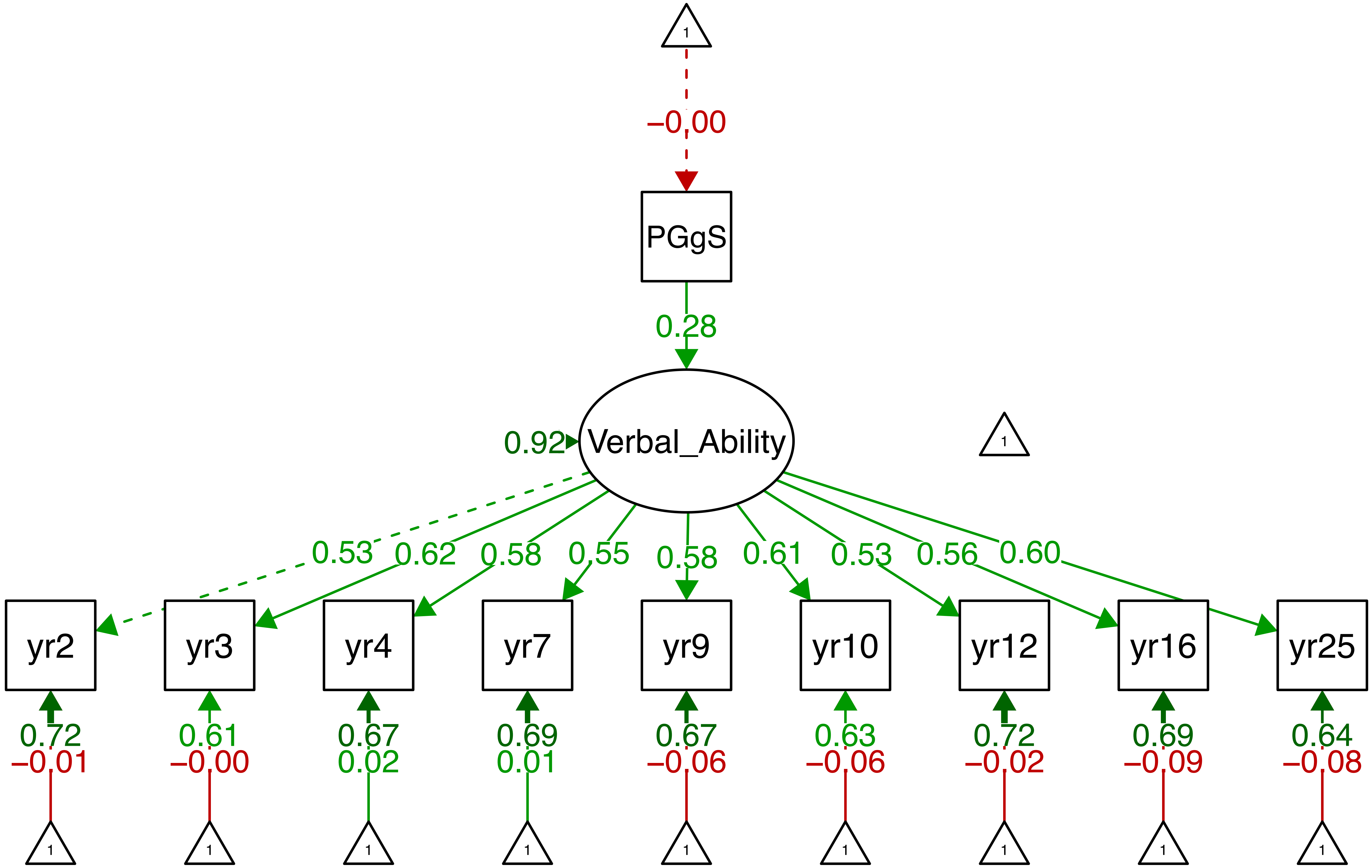

C10-3

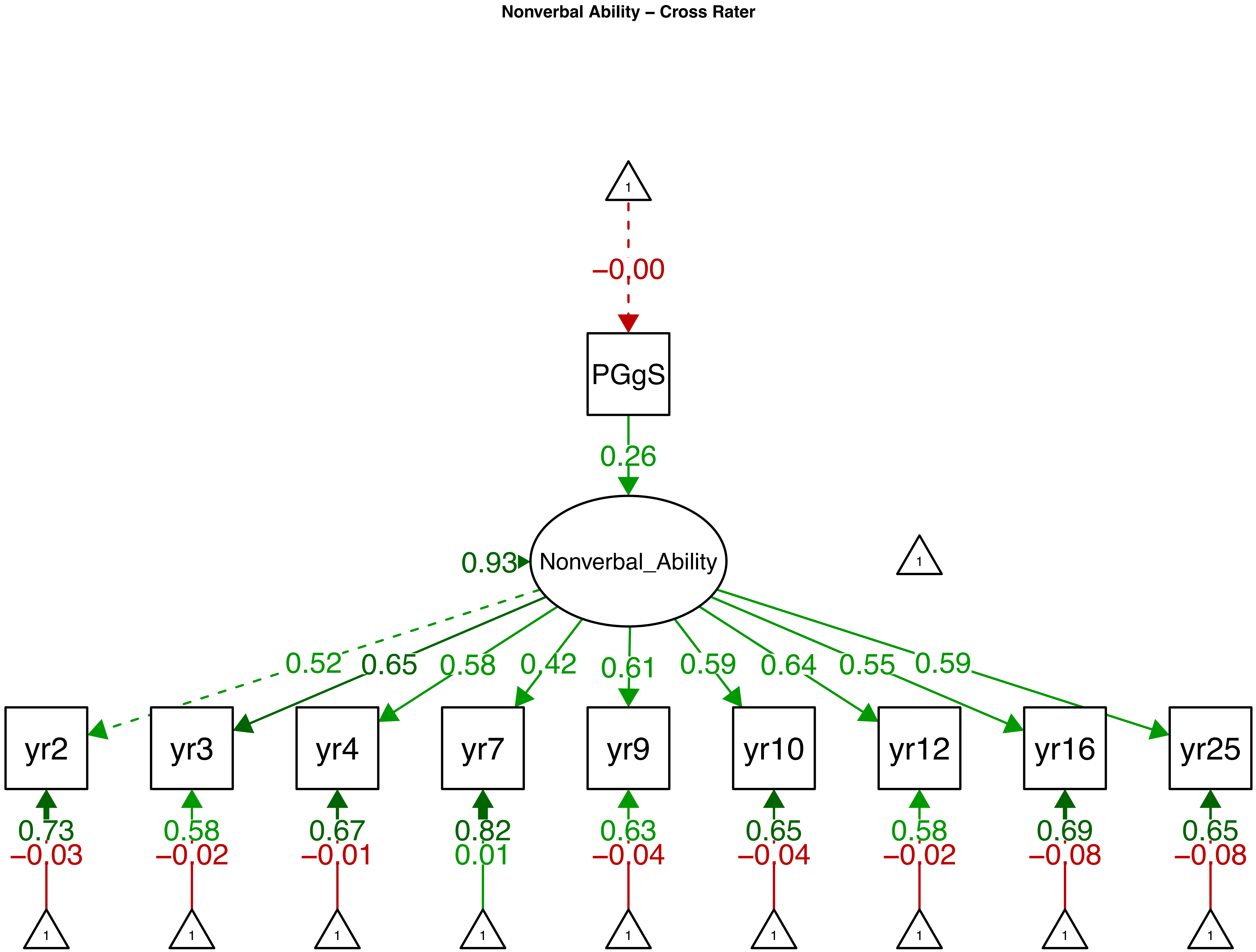

Nonverbal Ability – Cross Rater
1
–0.00
PGgS
0.26
0.93
Nonverbal_Ability
1
0.52
0.65
0.58
0.42
0.61
0.59
0.64
0.55
0.59
yr2
yr3
yr4
yr7
yr9
yr10
yr12
yr16
yr25
0.73
–0.03
0.58
–0.02
0.67
–0.01
0.82
0.01
0.63
–0.04
0.65
–0.04
0.58
–0.02
0.69
–0.08
0.65
–0.08
1
1
1
1
1
1
1
1
1

C10-4

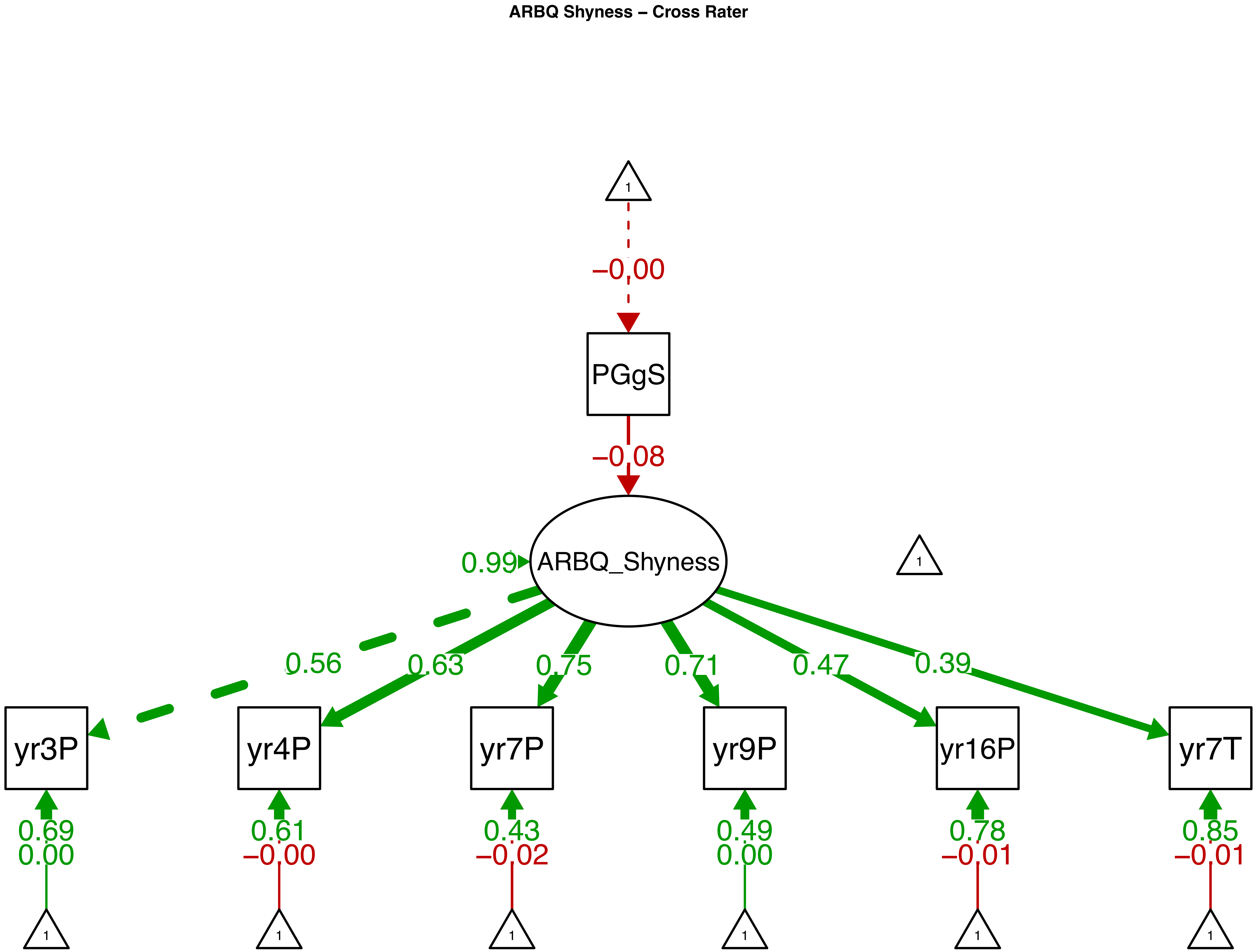
ARBQ Shyness – Cross Rater
1
−0.00
PGgS
−0.08
0.99
ARBQ_Shyness
1
0.56
0.63
0.75
0.71
0.47
0.39
yr3P
yr4P
yr7P
yr9P
yr16P
yr7T
0.69
0.00
0.61
−0.00
0.43
−0.02
0.49
0.00
0.78
−0.01
0.85
−0.01
1
1
1
1
1
1

C10-5

ARBQ Fear – Cross Rater

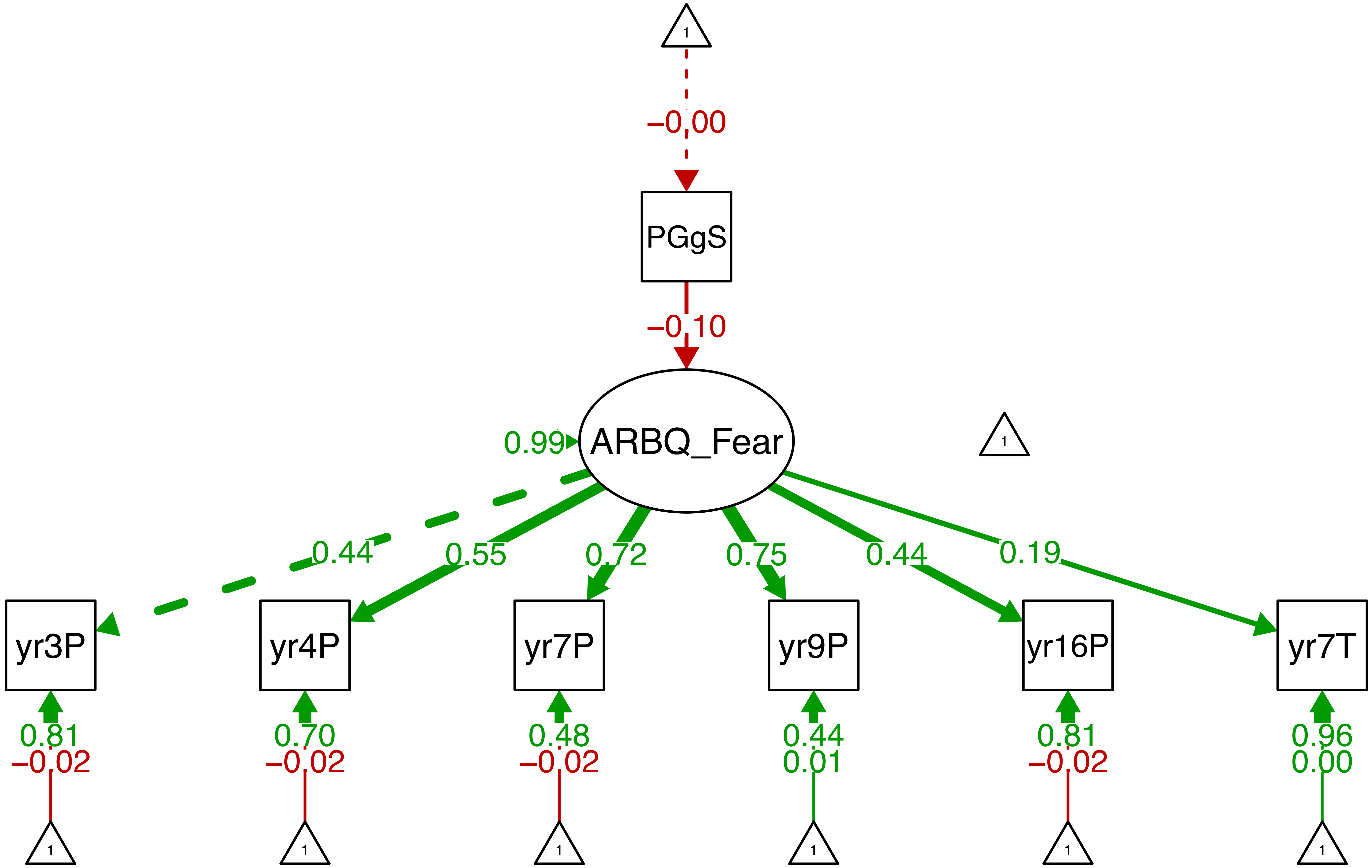

C10-6

ARBQ Obsessive–Compulsive – Cross Rater

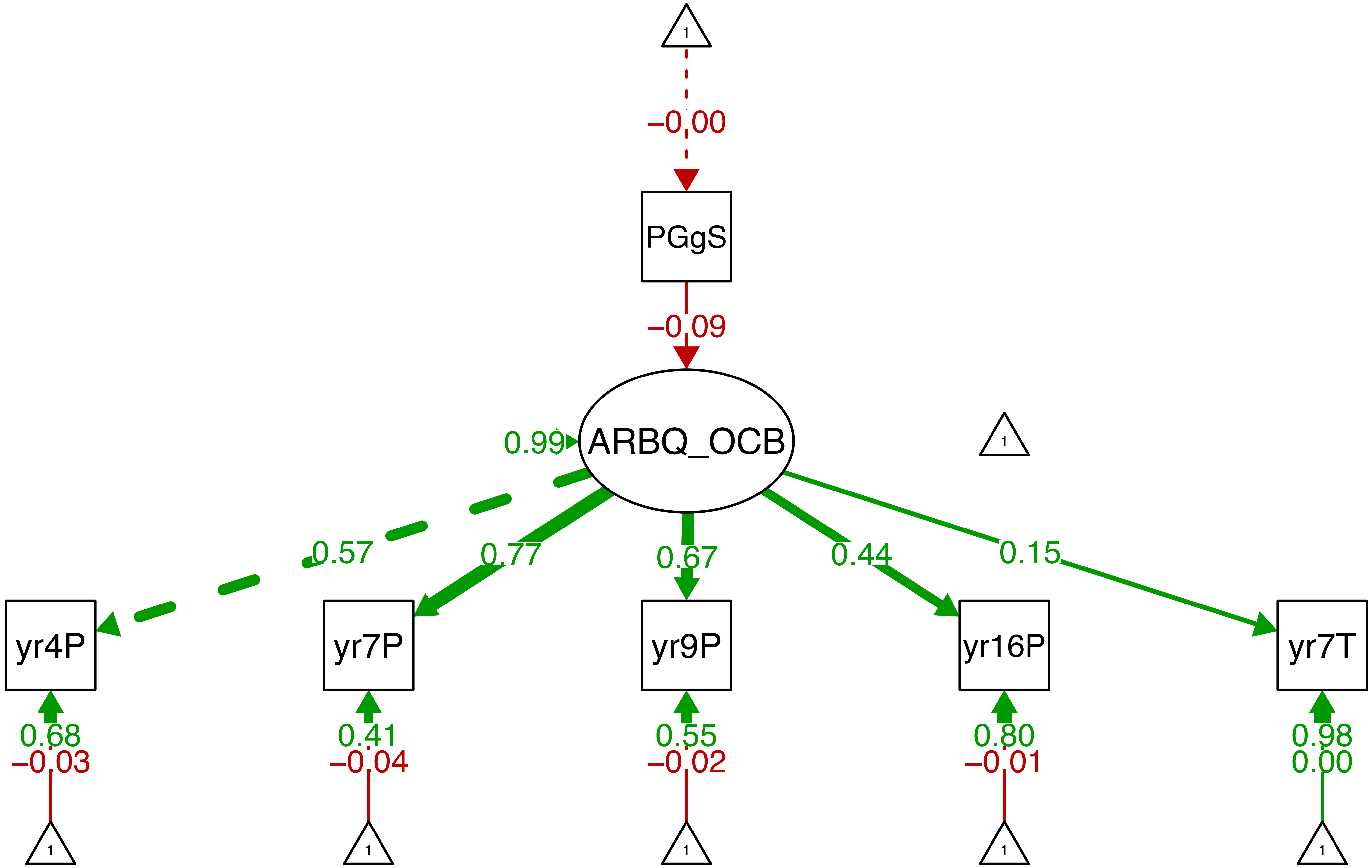

ARBQ Negative Affect – Cross Rater

C10-7

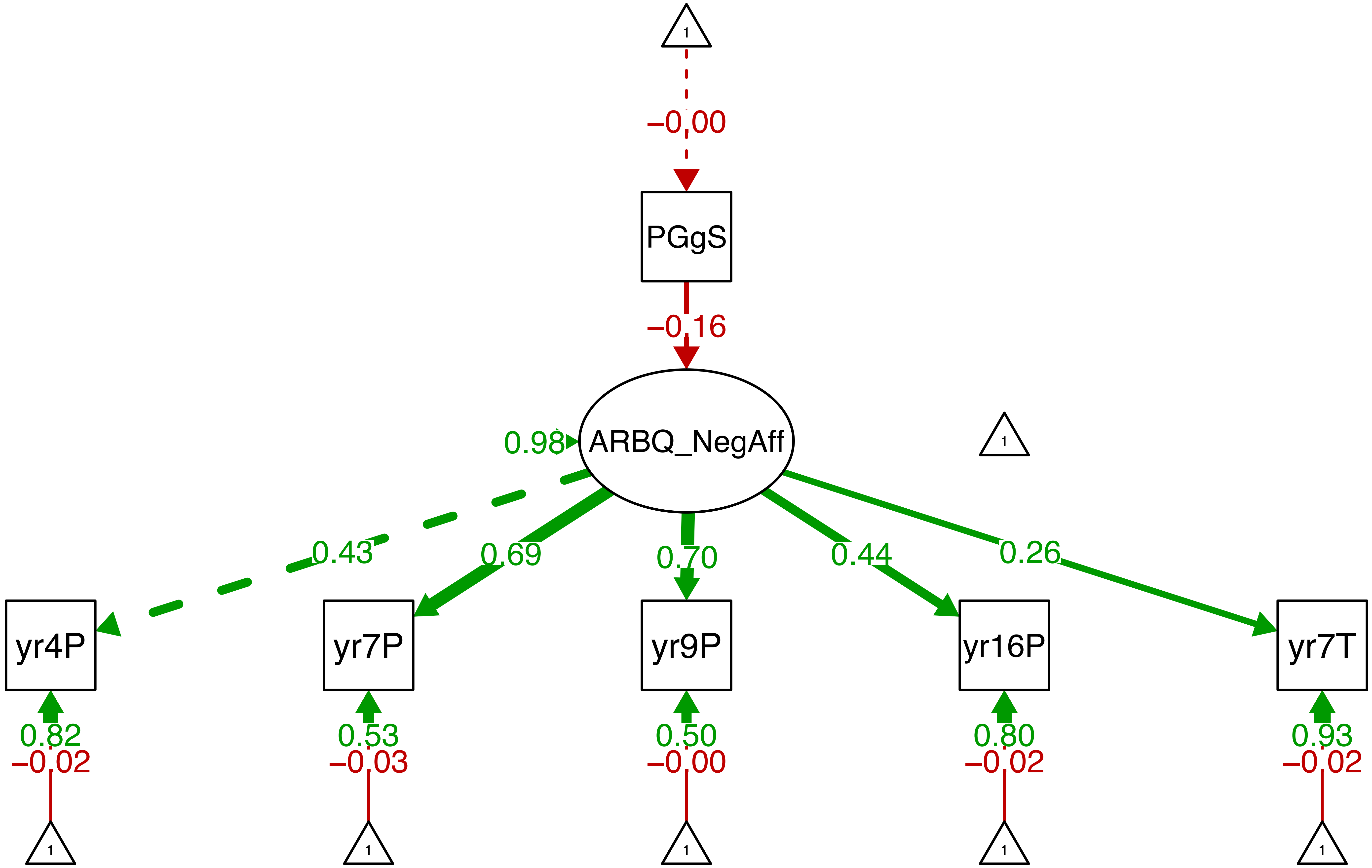

C10-8

ARBQ Negative Cognition – Cross Rater

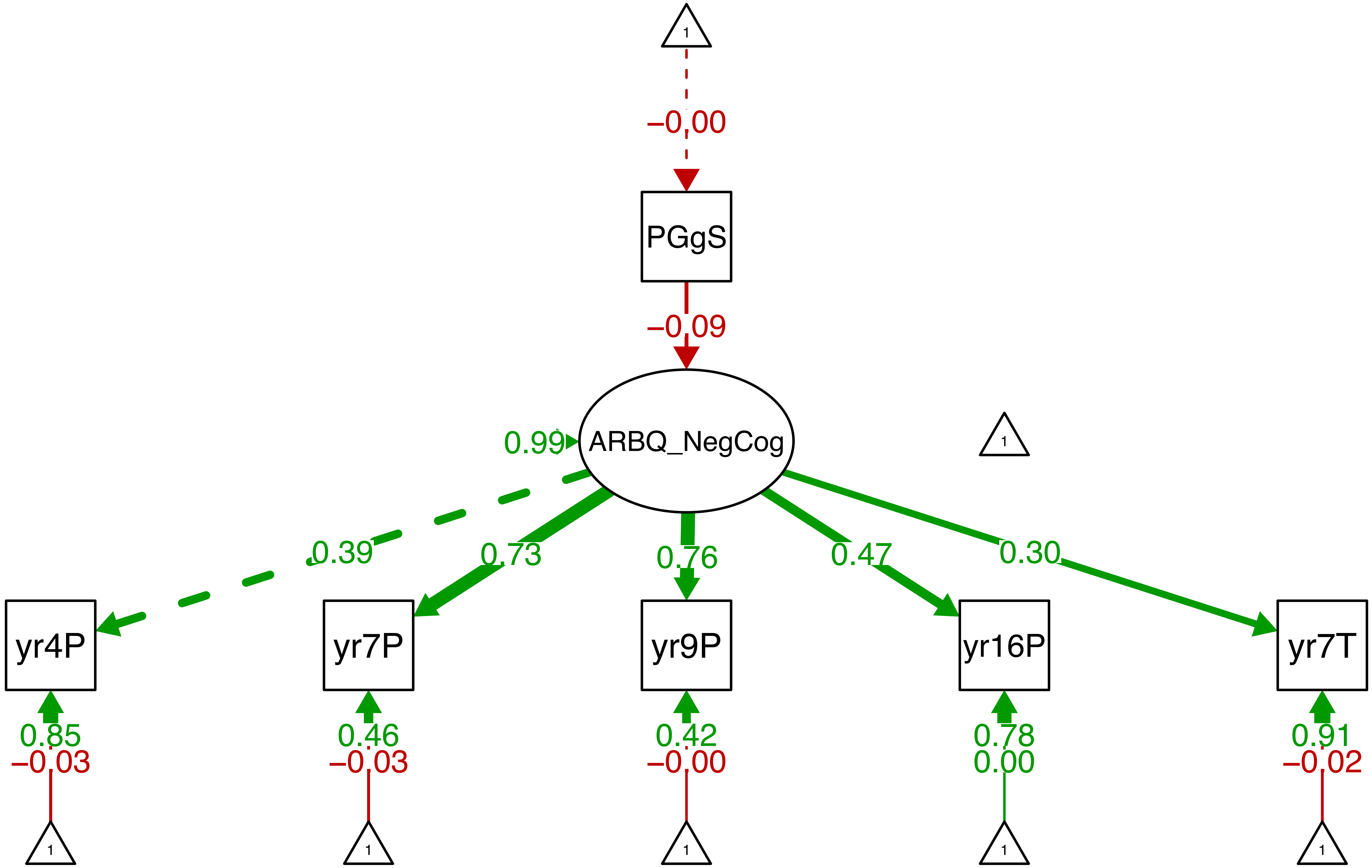

C10-9

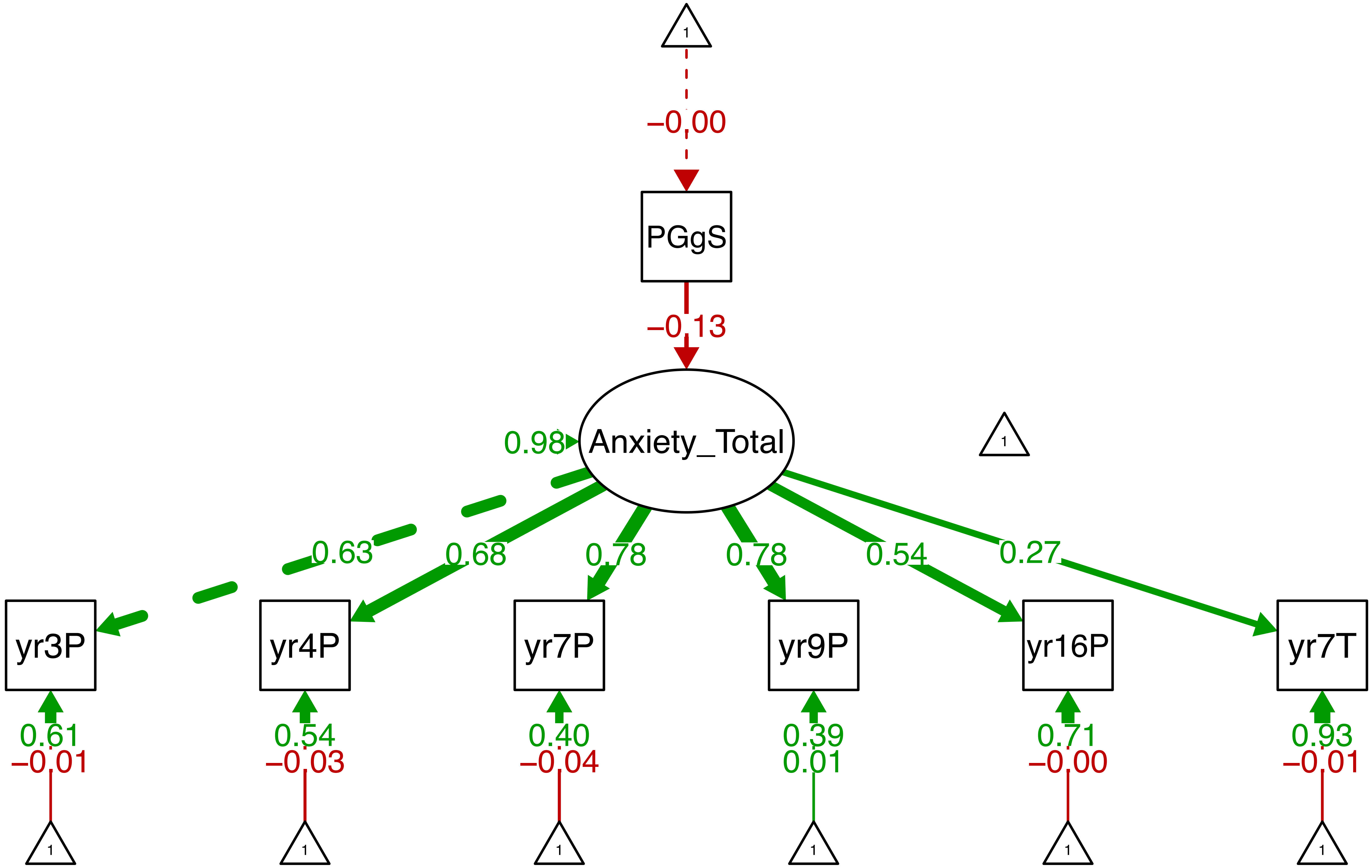
1
−0.00
PGgS
−0.13
0.98
Anxiety_Total
1
0.63
0.68
0.78
0.78
0.54
0.27
yr3P
yr4P
yr7P
yr9P
yr16P
yr7T
0.61
−0.01
0.54
−0.03
0.40
−0.04
0.39
0.01
0.71
−0.00
0.93
−0.01
1
1
1
1
1
1

Conners Inattention – Cross Rater

C10-10

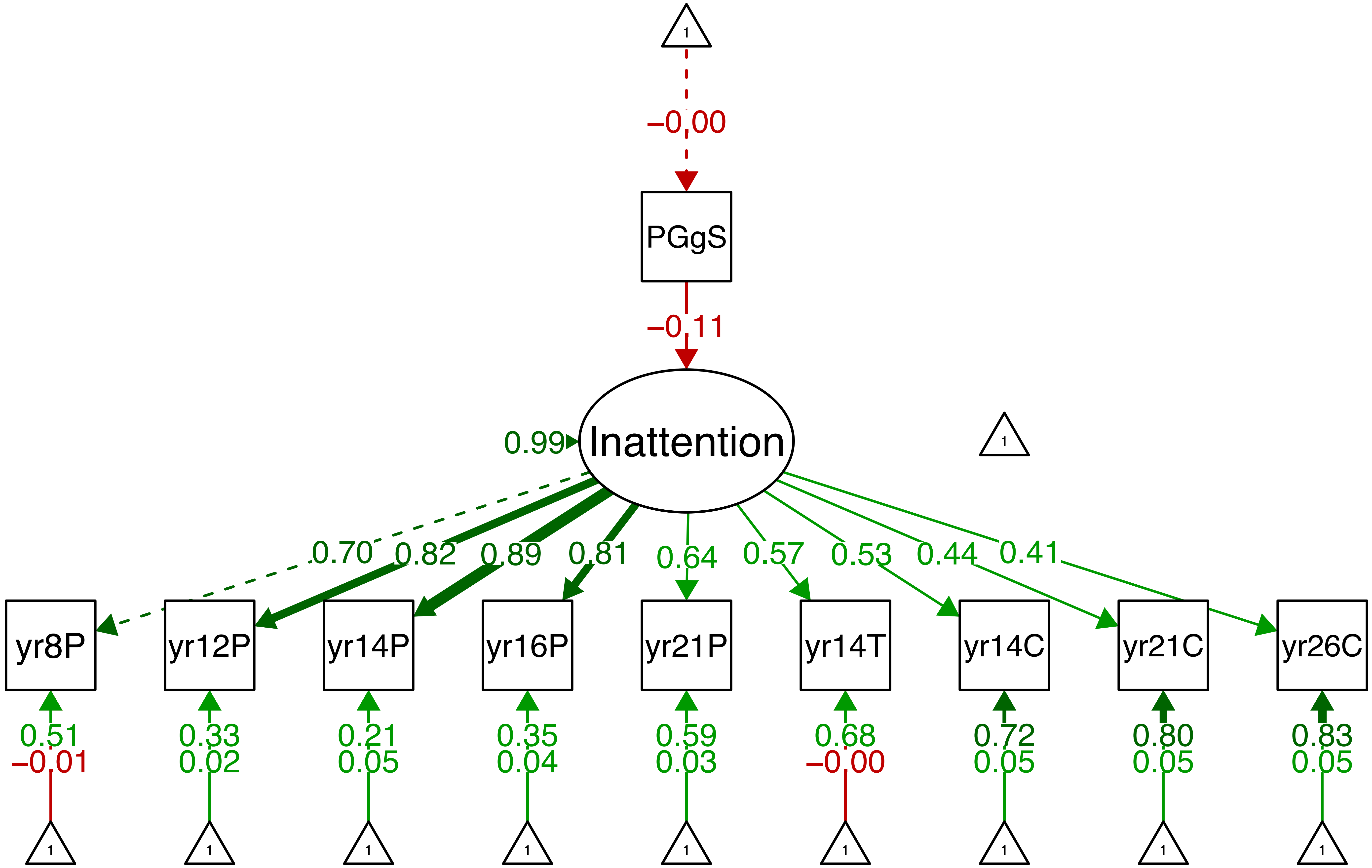

Conners Inattention – Parent Only

C10-11

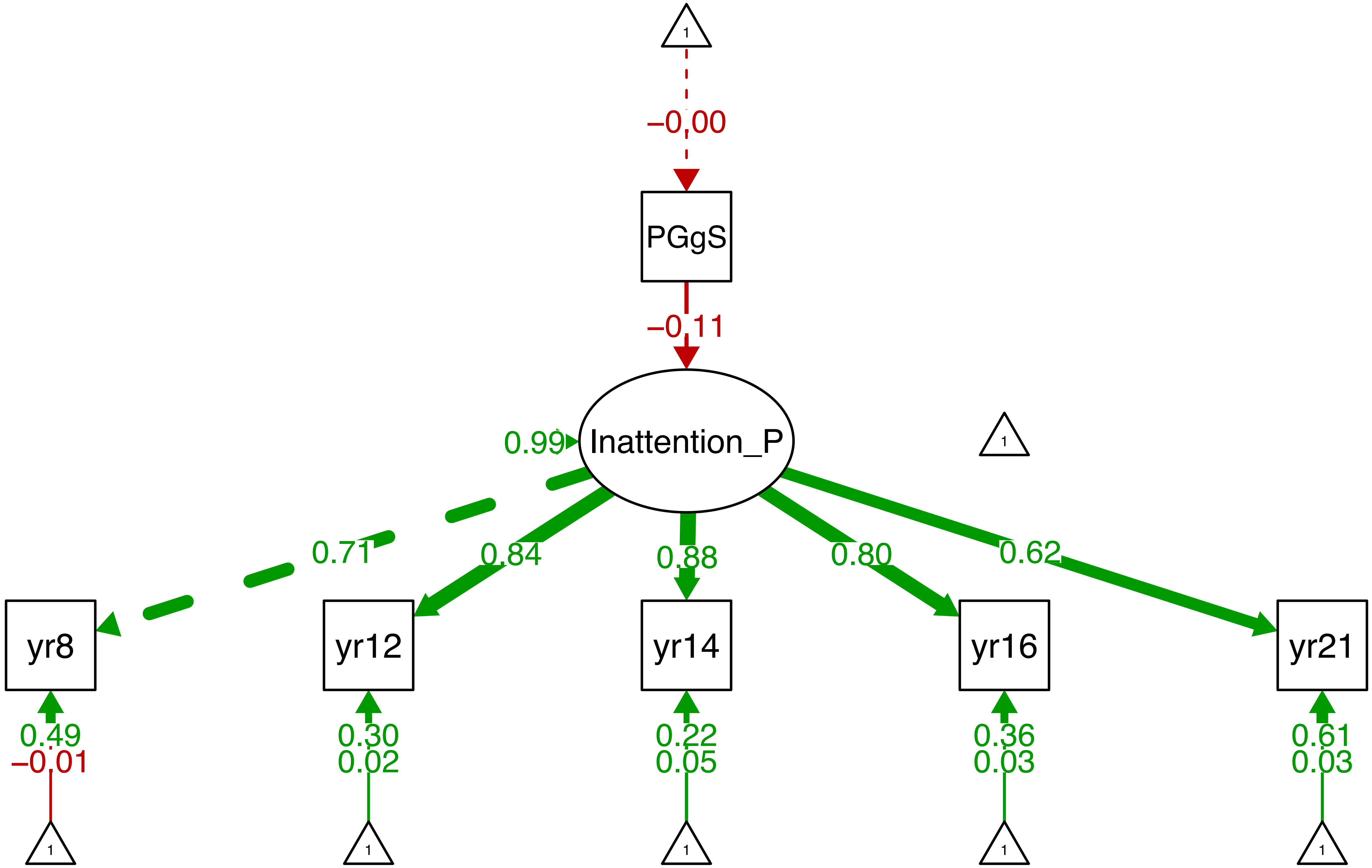

Conners Hyperactivity–Impulsivity – Cross Rater

C10-12

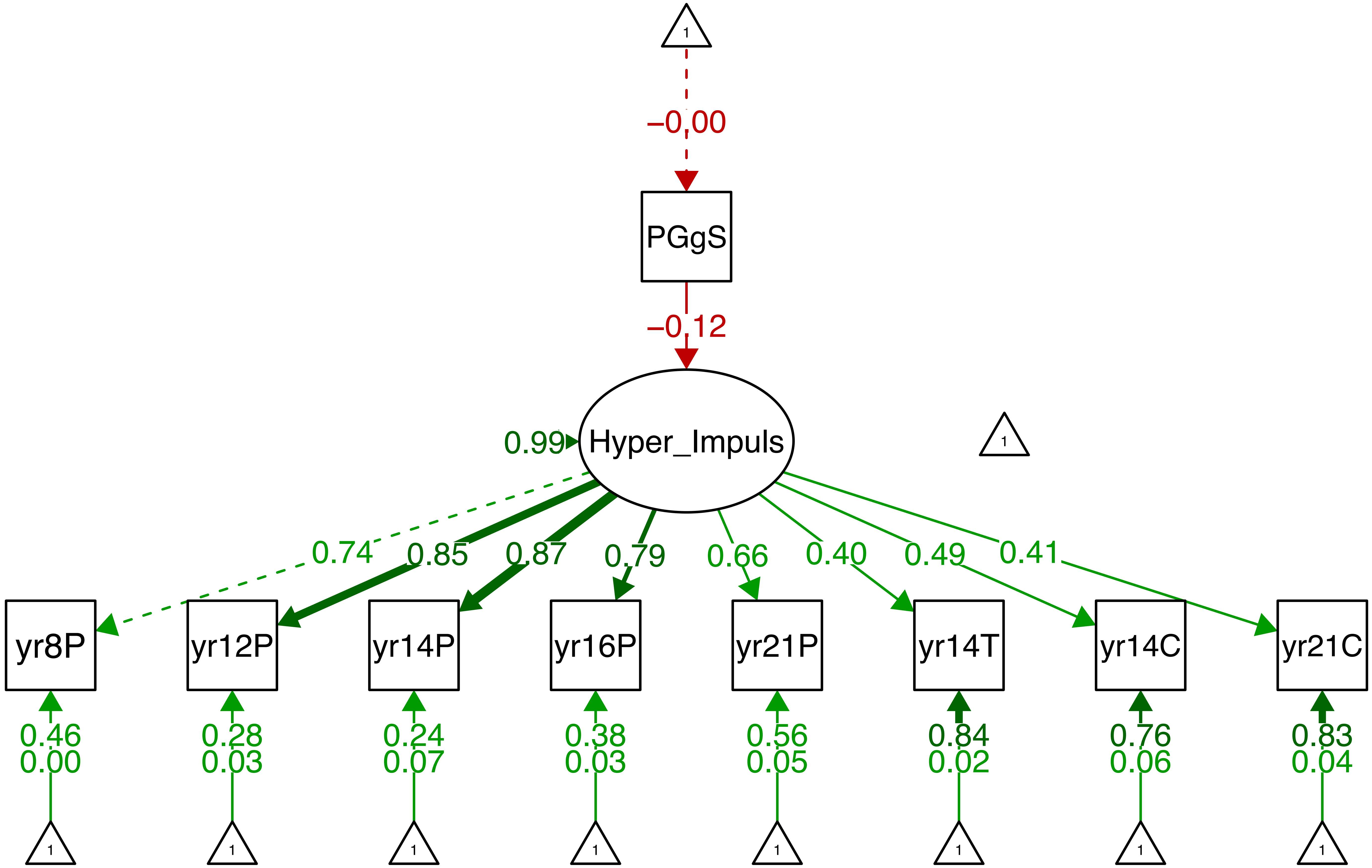

C10-13

Conners Hyperactivity–Impulsivity – Parent Only

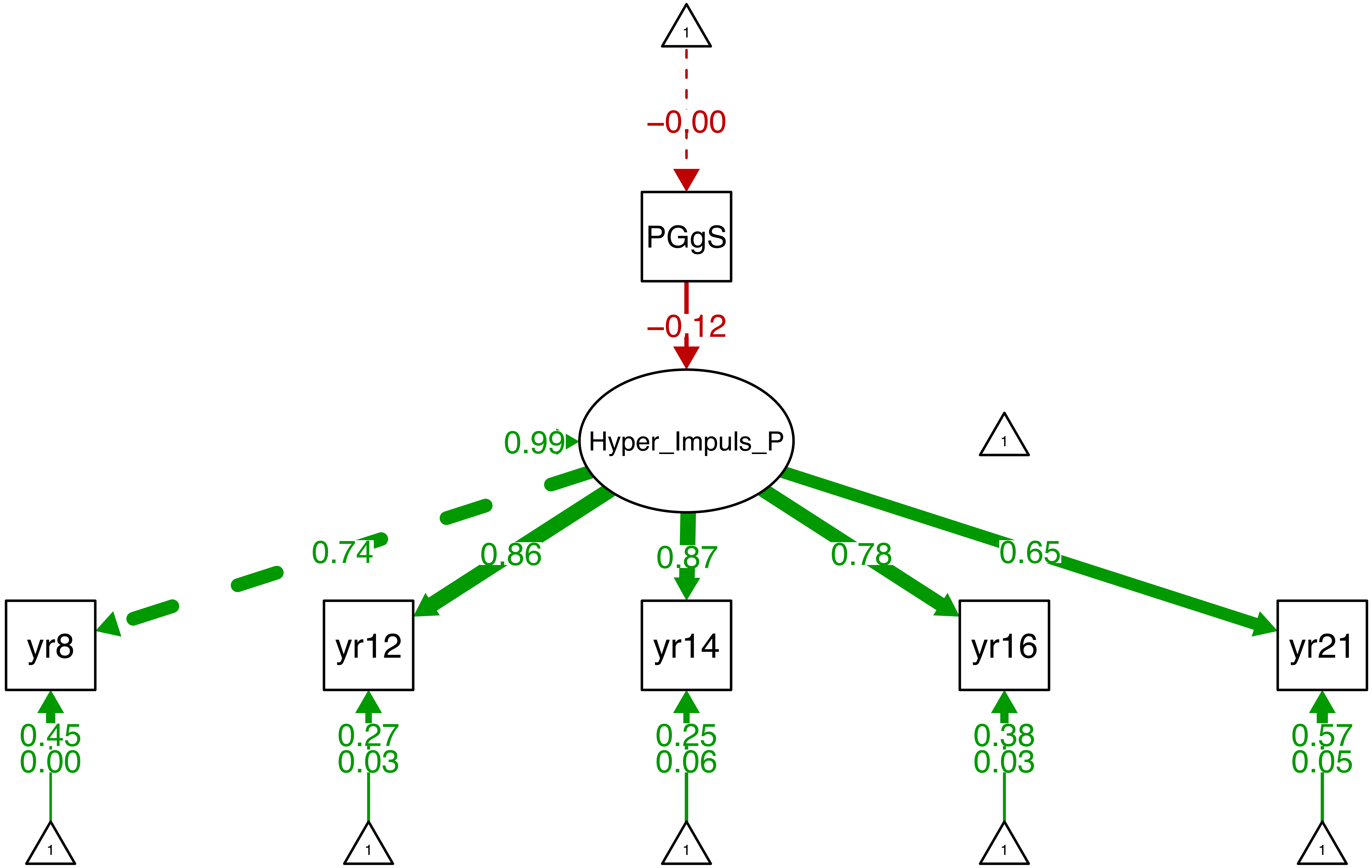

Conners Total – Cross Rater

C10-14

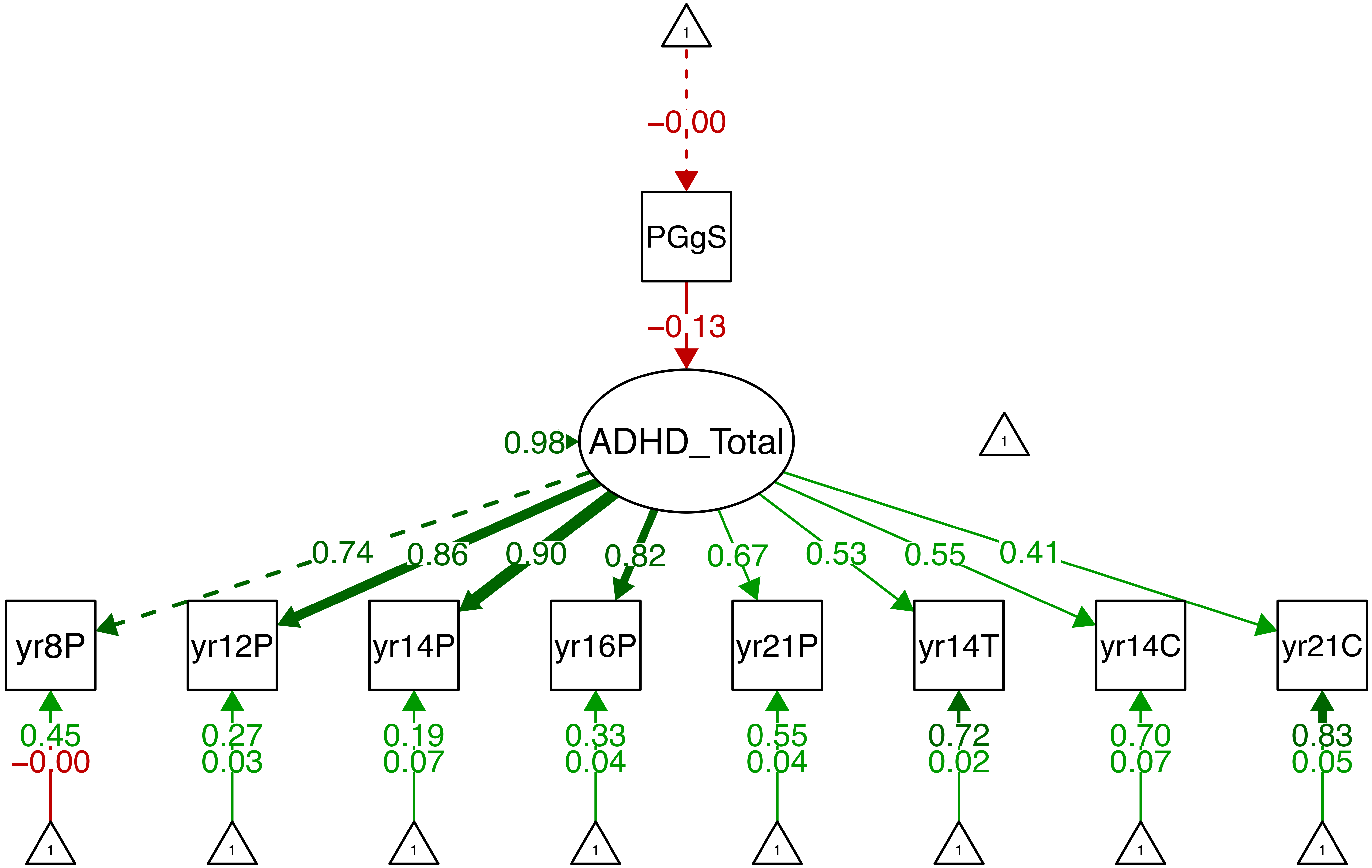

Conners Total – Parent Only

C10-15

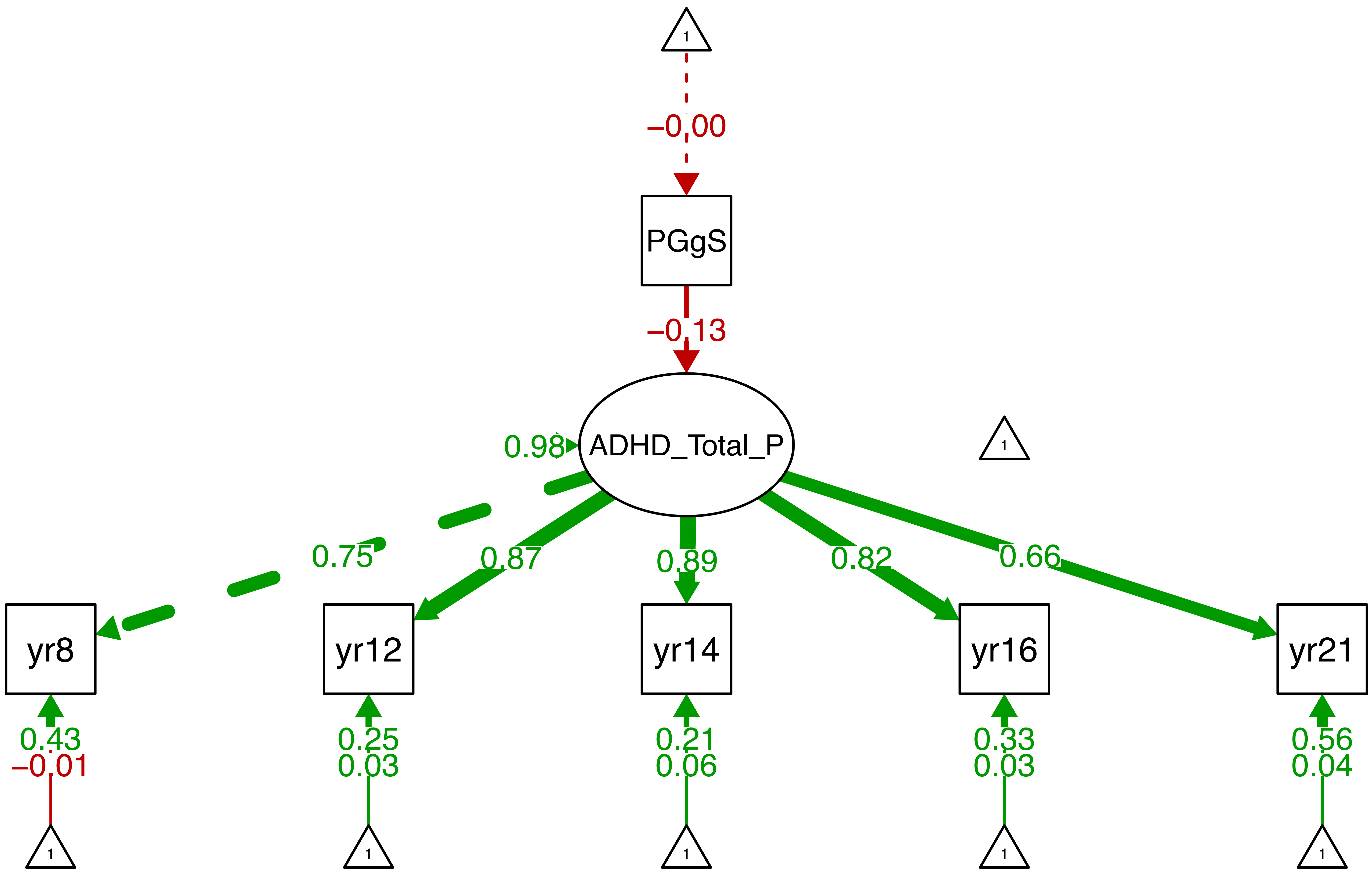

SDQ Conduct – Parent Only

C10-16

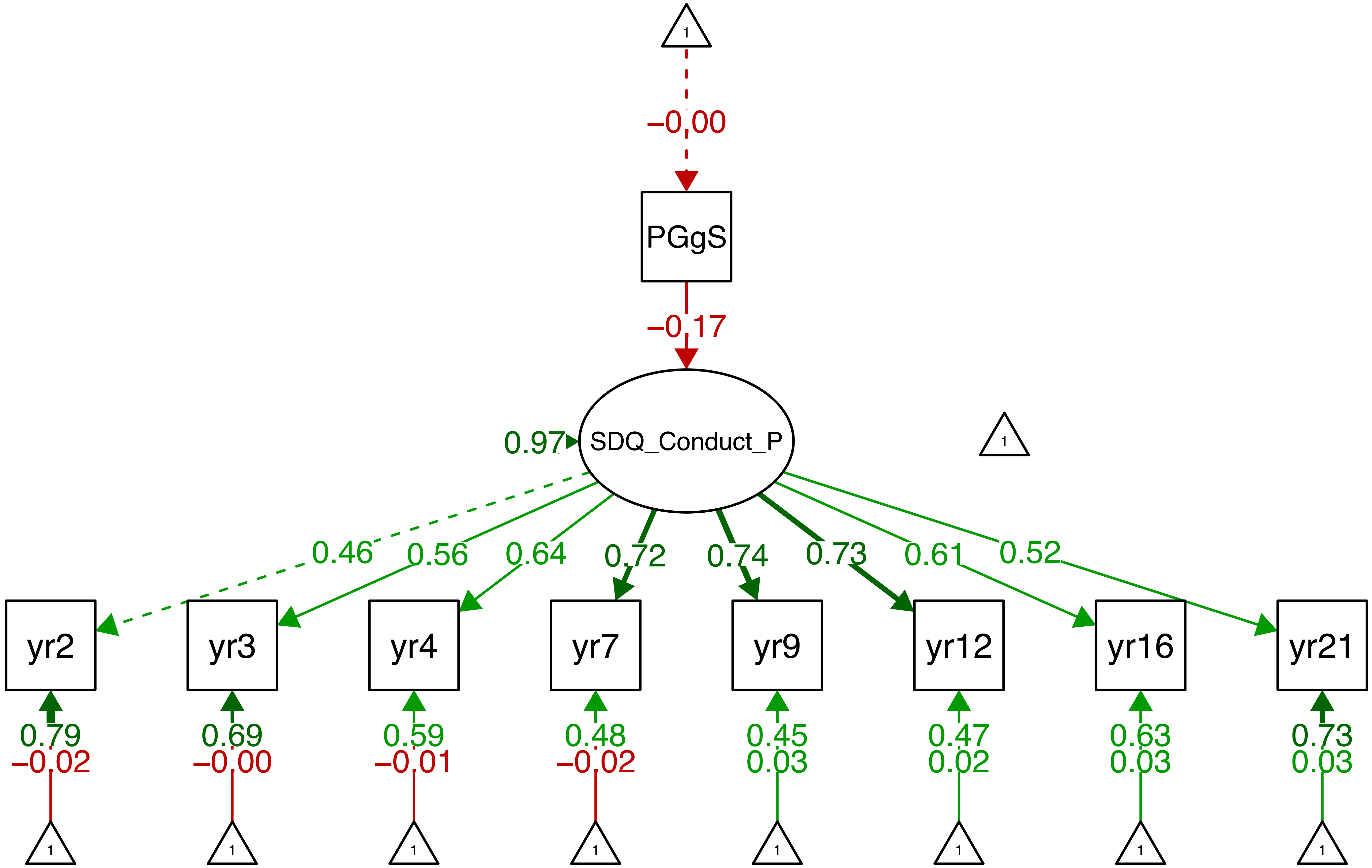

SDQ Conduct – Teacher Only

C10-17

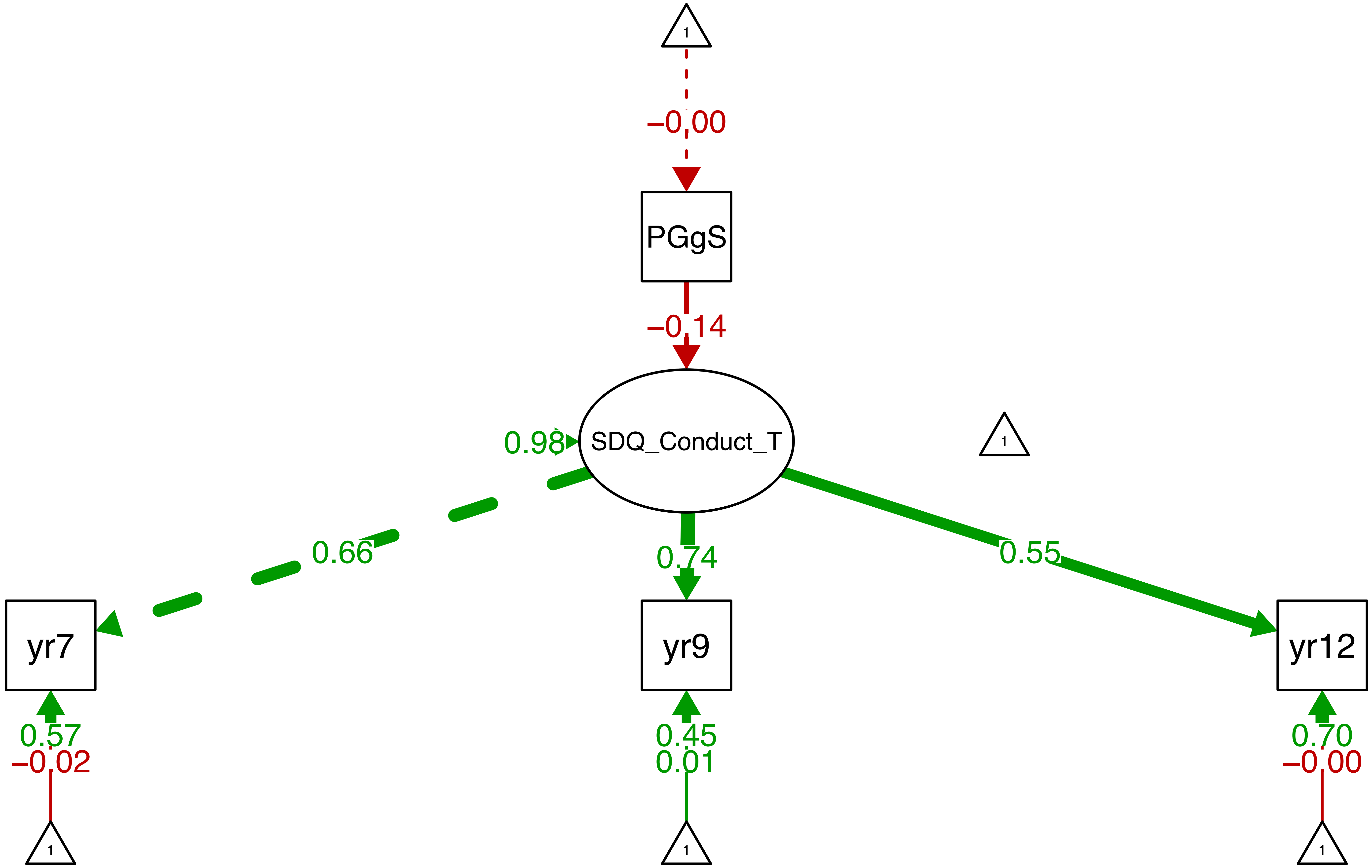

C10-18

SDQ Conduct – Child Only

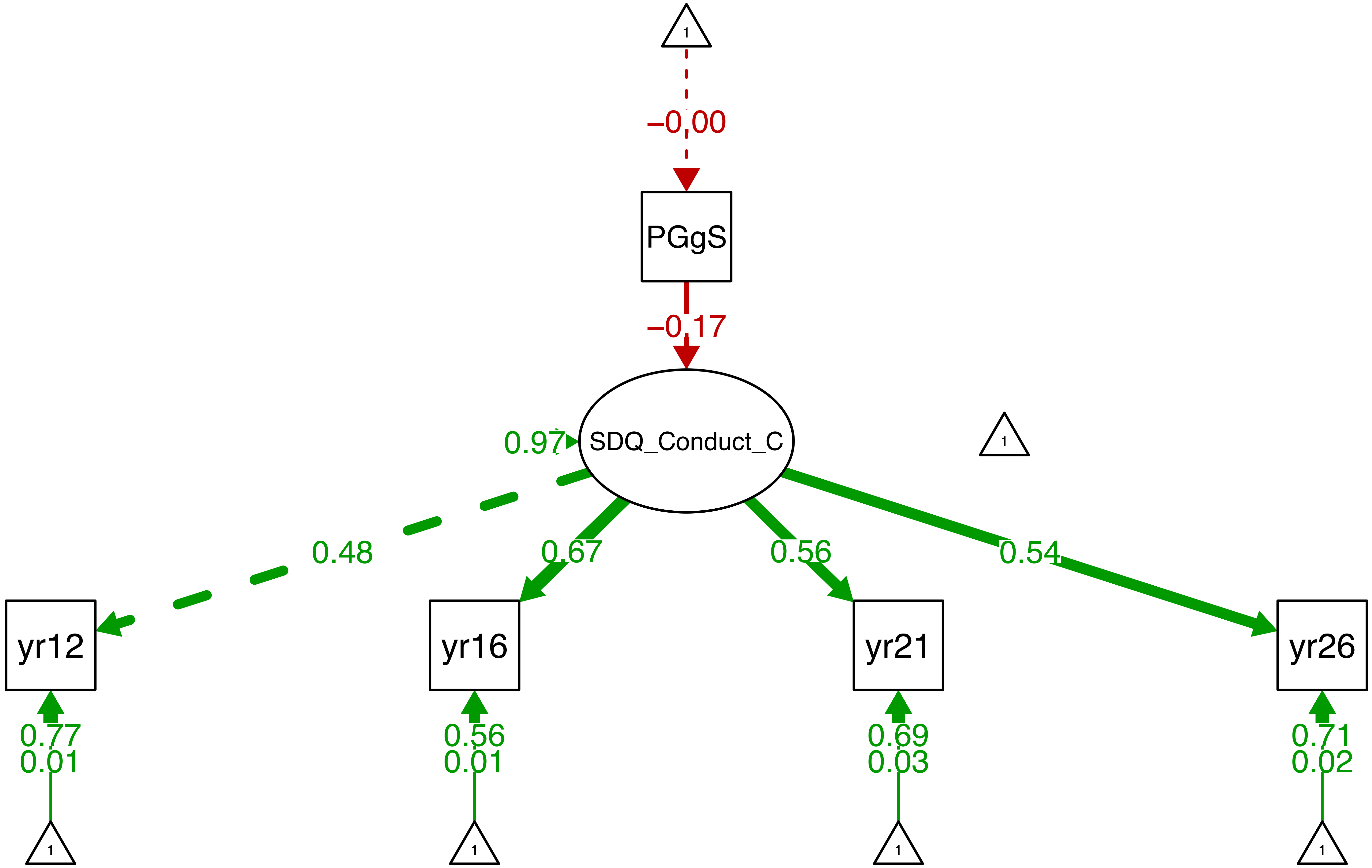

C10-19

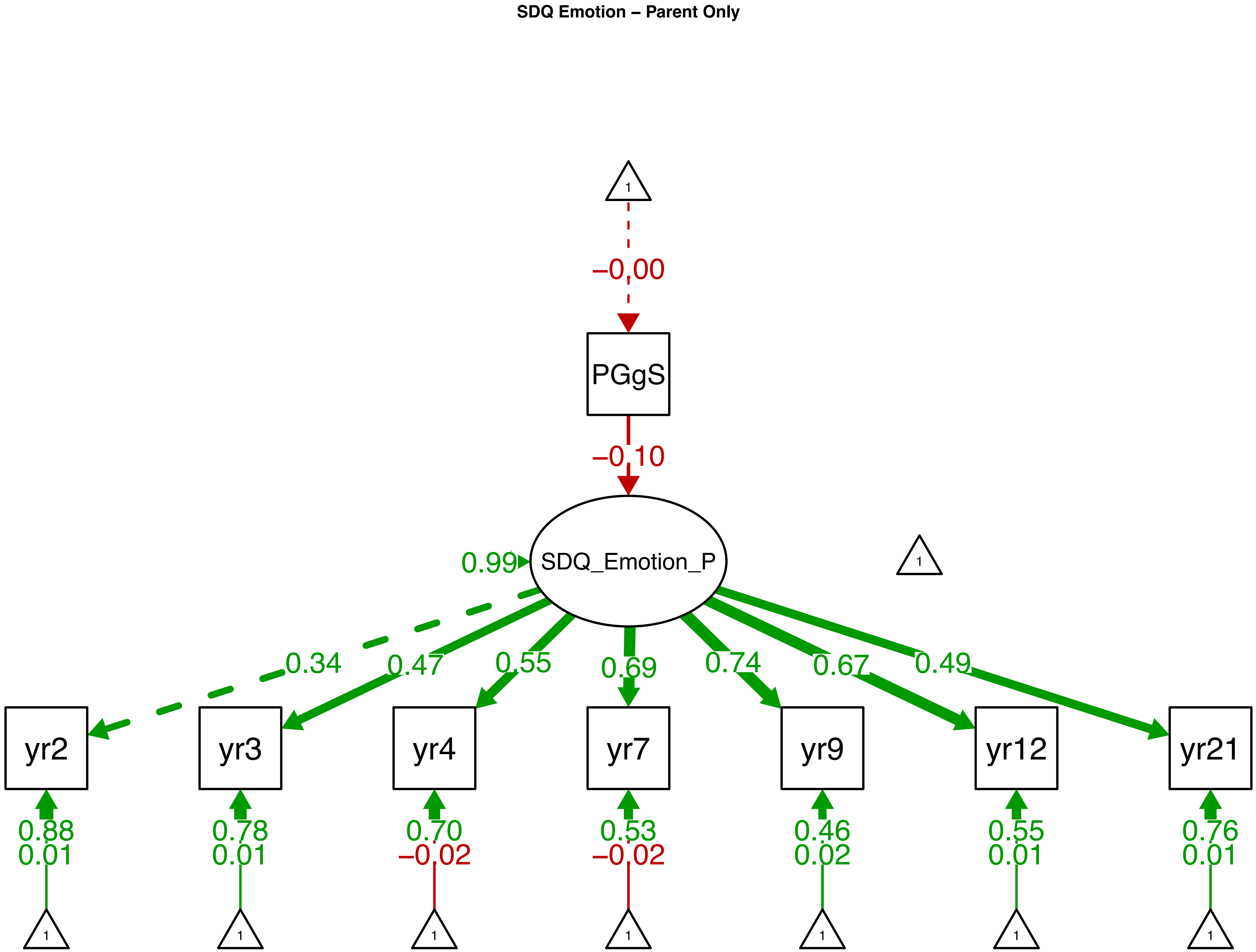
SDQ Emotion – Parent Only
1
−0.00
PGgS
−0.10
0.99
SDQ_Emotion_P
1
0.34
0.47
0.55
0.69
0.74
0.67
0.49
yr2
yr3
yr4
yr7
yr9
yr12
yr21
0.88
0.01
0.78
0.01
0.70
−0.02
0.53
−0.02
0.46
0.02
0.55
0.01
0.76
0.01
1
1
1
1
1
1
1

SDQ Emotion – Teacher Only

C10-20

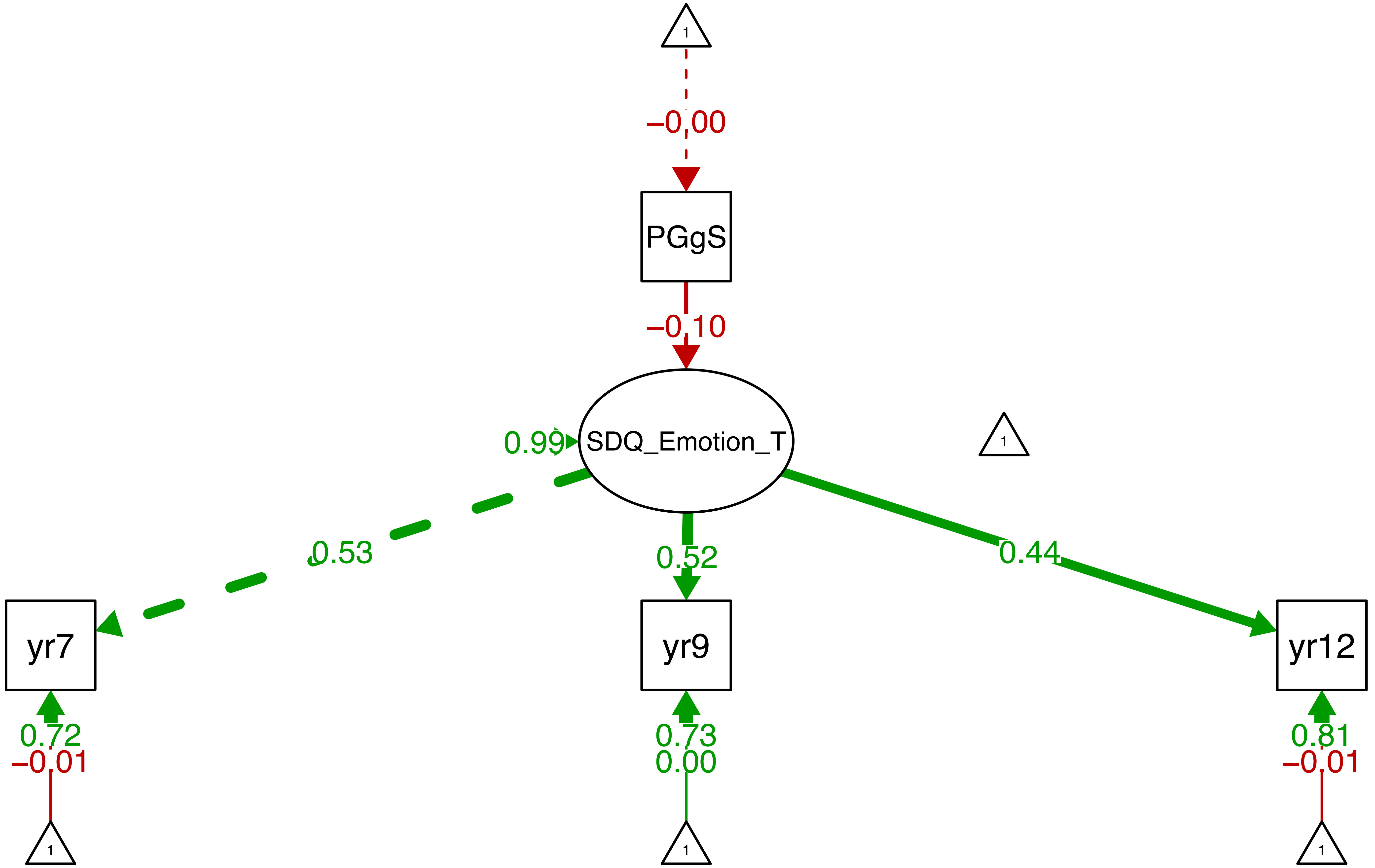

SDQ Emotion – Child Only

C10-21

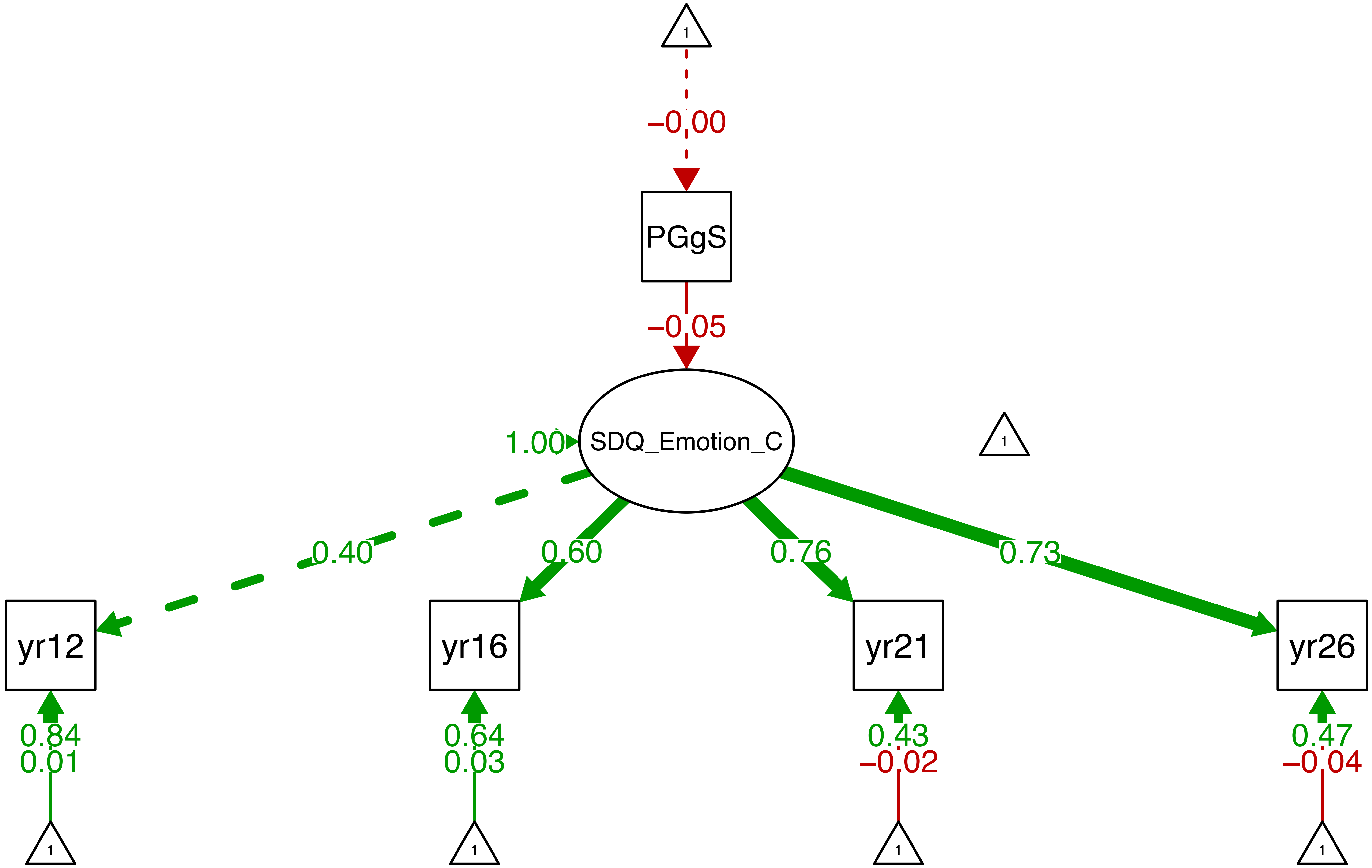

C10-22

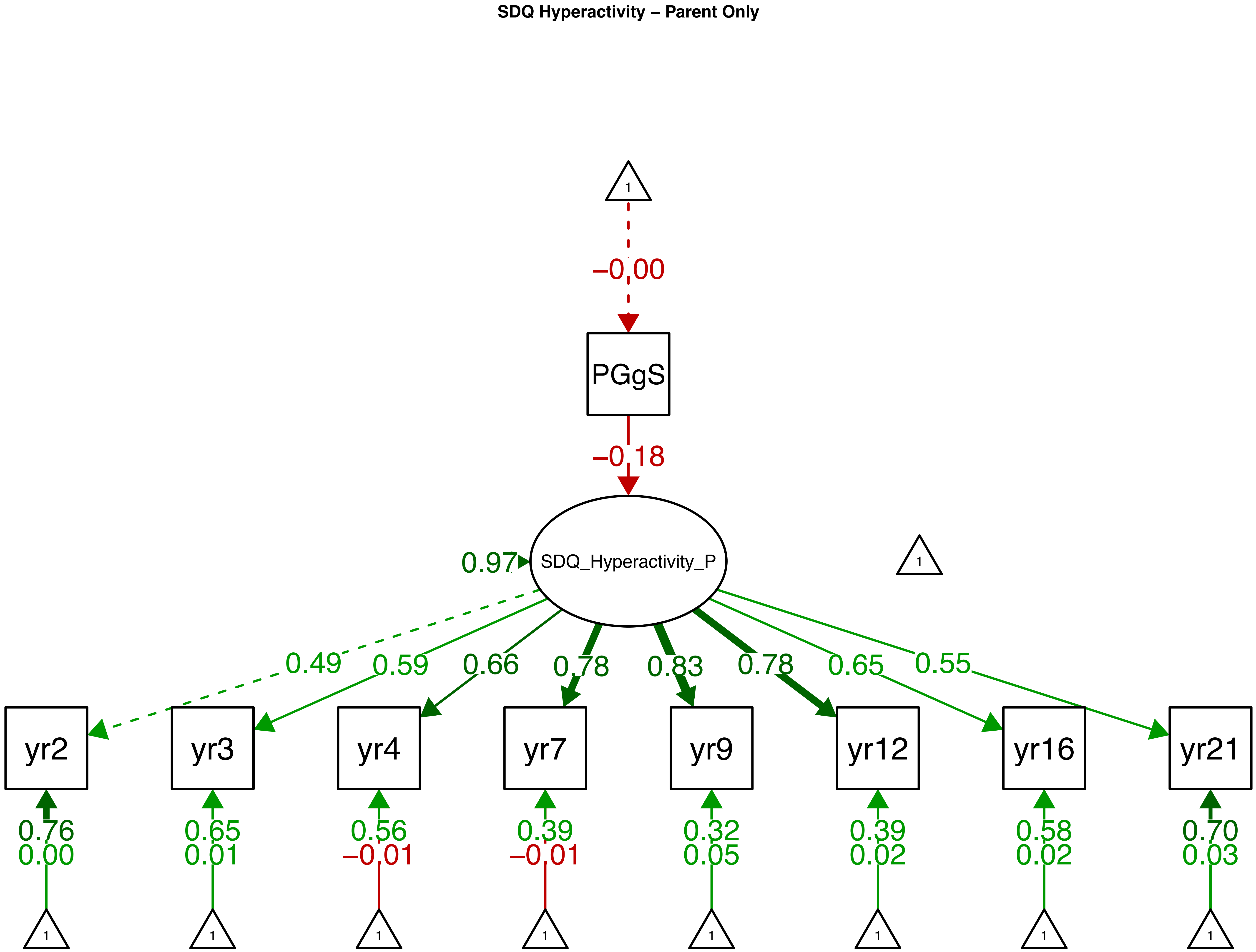

SDQ Hyperactivity – Parent Only
1
−0.00
PGgS
−0.18
0.97
SDQ_Hyperactivity_P
1
0.49
0.59
0.66
0.78
0.83
0.78
0.65
0.55
yr2
yr3
yr4
yr7
yr9
yr12
yr16
yr21
0.76
0.00
0.65
0.01
0.56
−0.01
0.39
−0.01
0.32
0.05
0.39
0.02
0.58
0.02
0.70
0.03

C10-23

SDQ Hyperactivity – Teacher Only

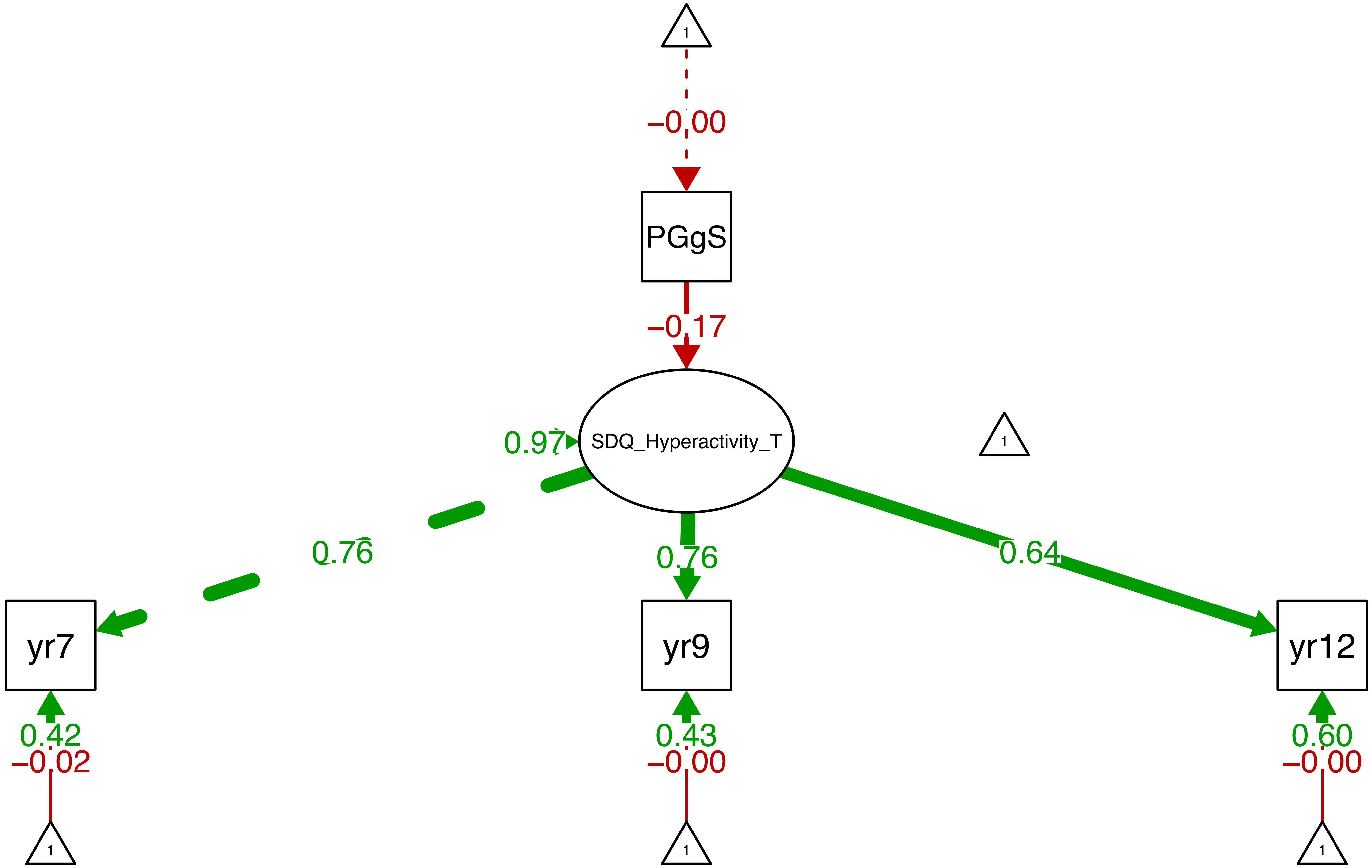

SDQ Hyperactivity – Child Only

C10-24

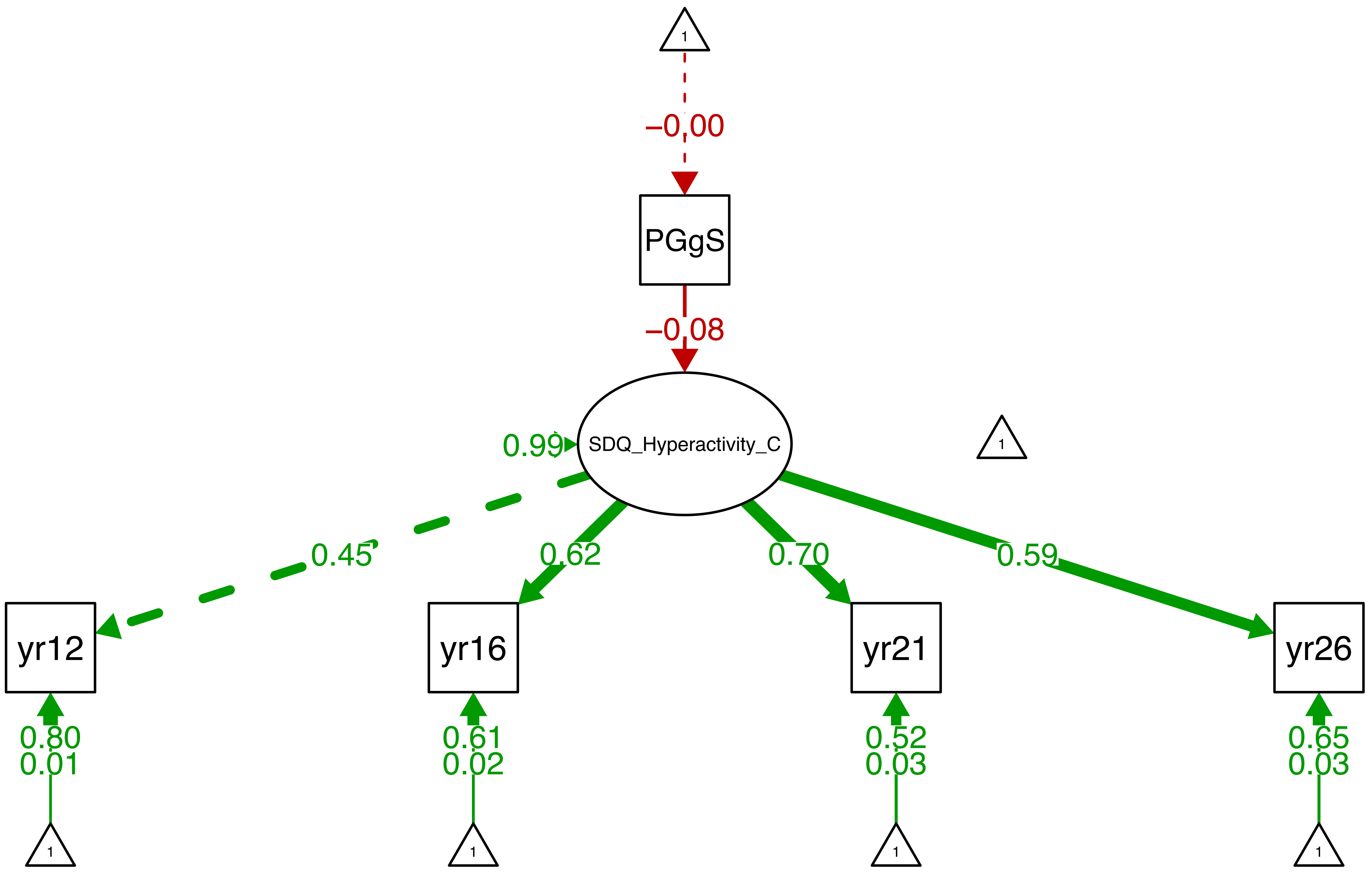

SDQ Peer Problems – Parent Only

C10-25

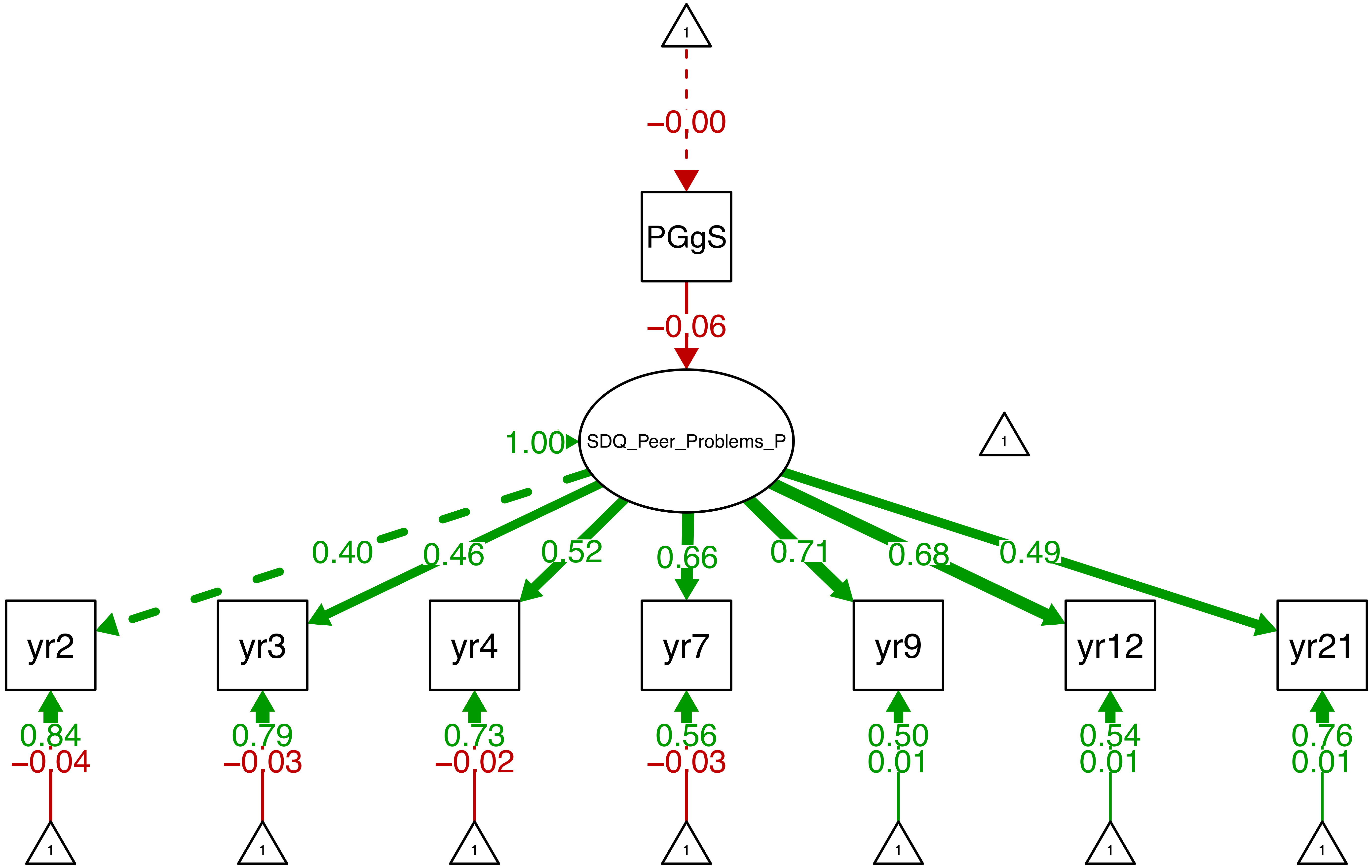

C10-26

SDQ Peer Problems – Teacher Only

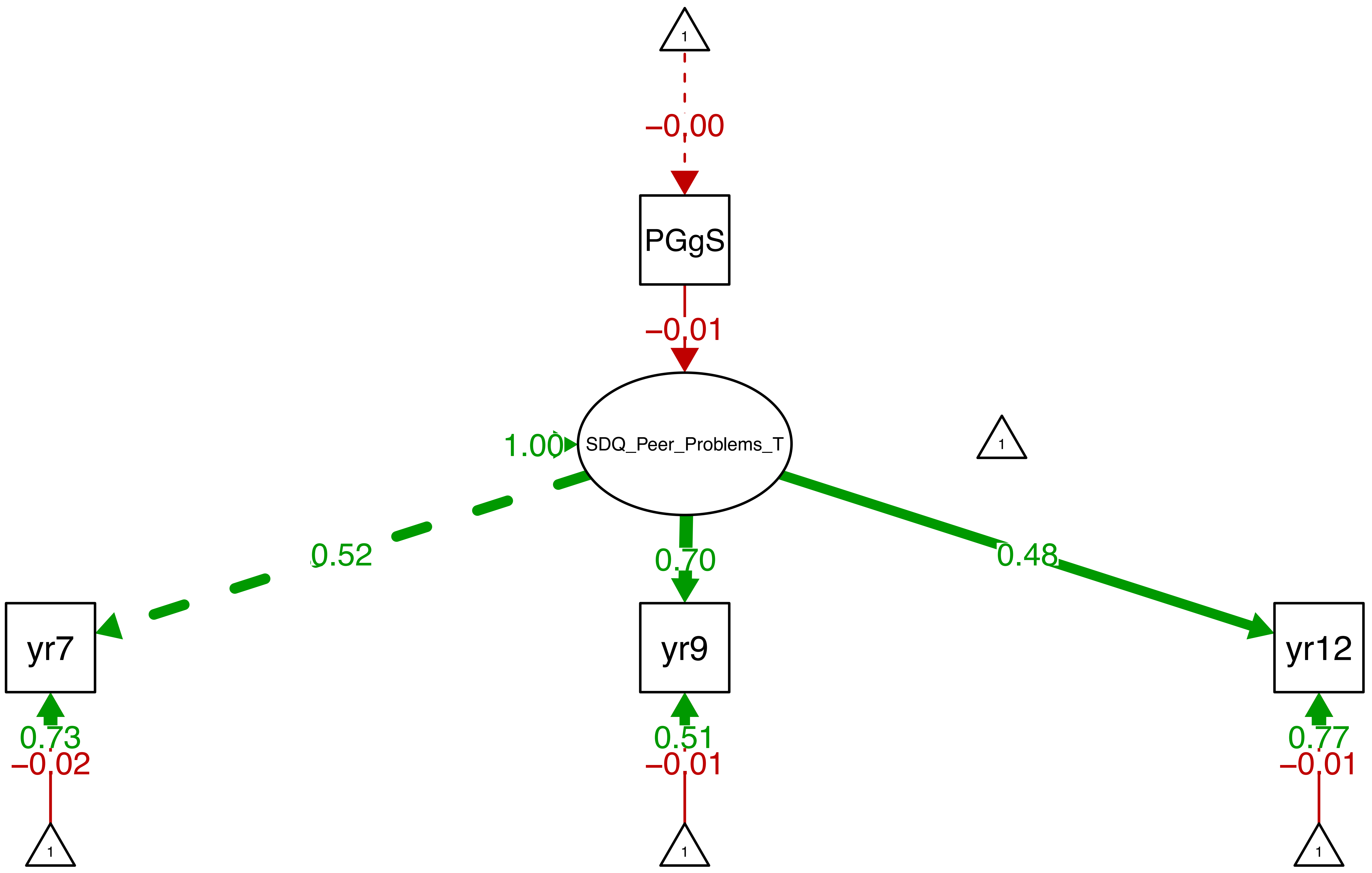

C10-27

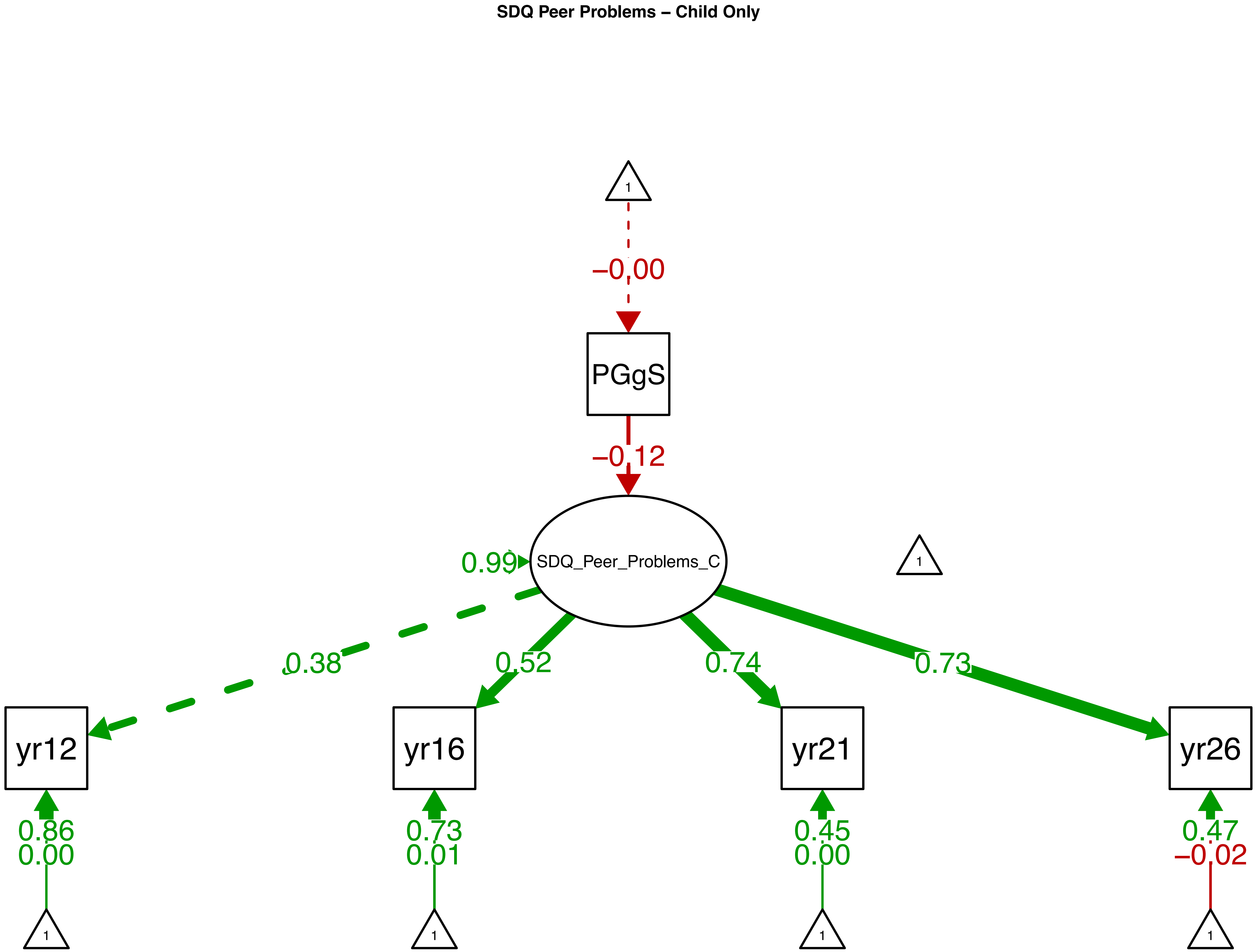
SDQ Peer Problems – Child Only
1
−0.00
PGgS
−0.12
0.99
SDQ_Peer_Problems_C
1
0.38
0.52
0.74
0.73
yr12
yr16
yr21
yr26
0.86
0.00
0.73
0.01
0.45
0.00
0.47
−0.02
1
1
1
1

SDQ Prosocial – Parent Only

C10-28

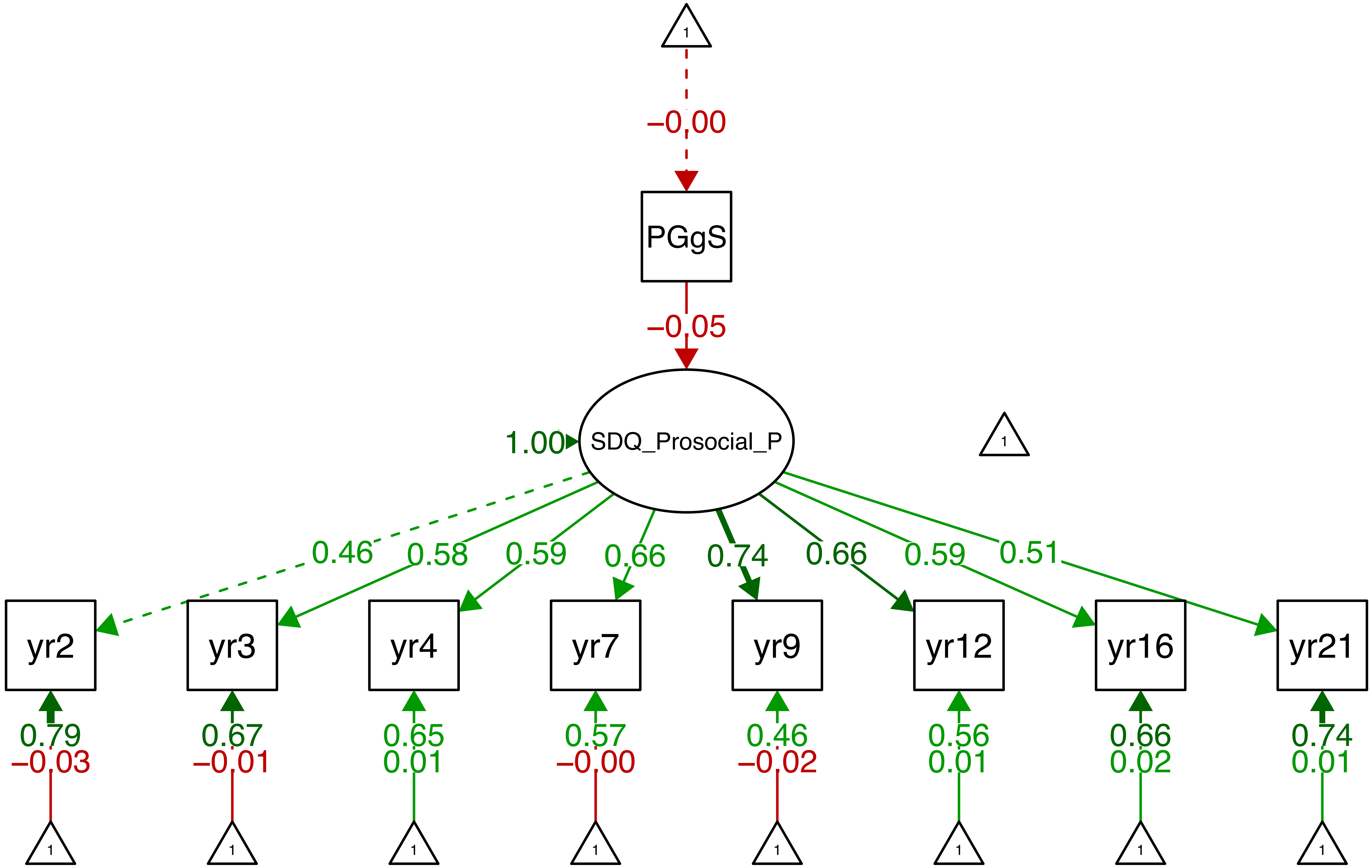

SDQ Prosocial – Teacher Only

C10-29

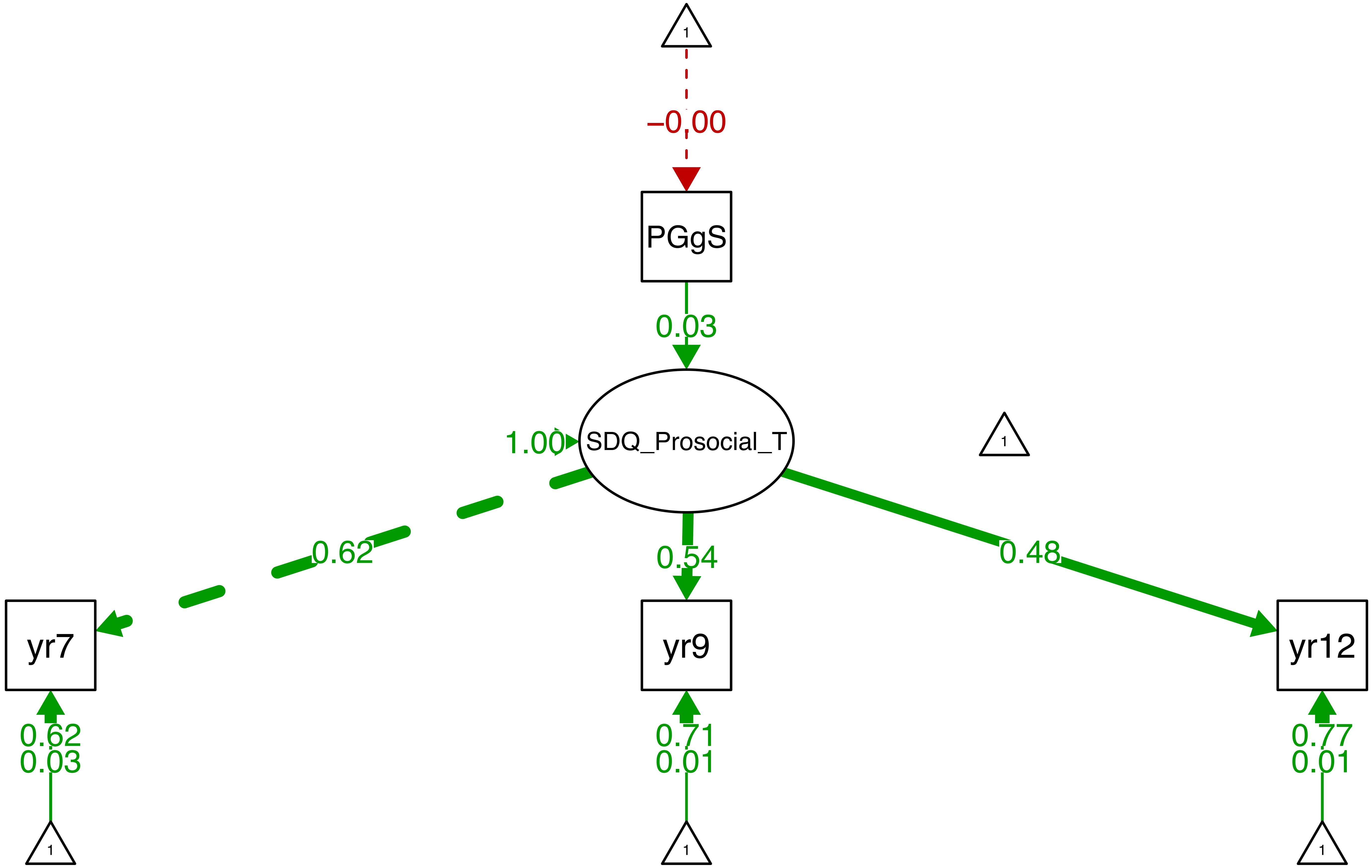

C10-30

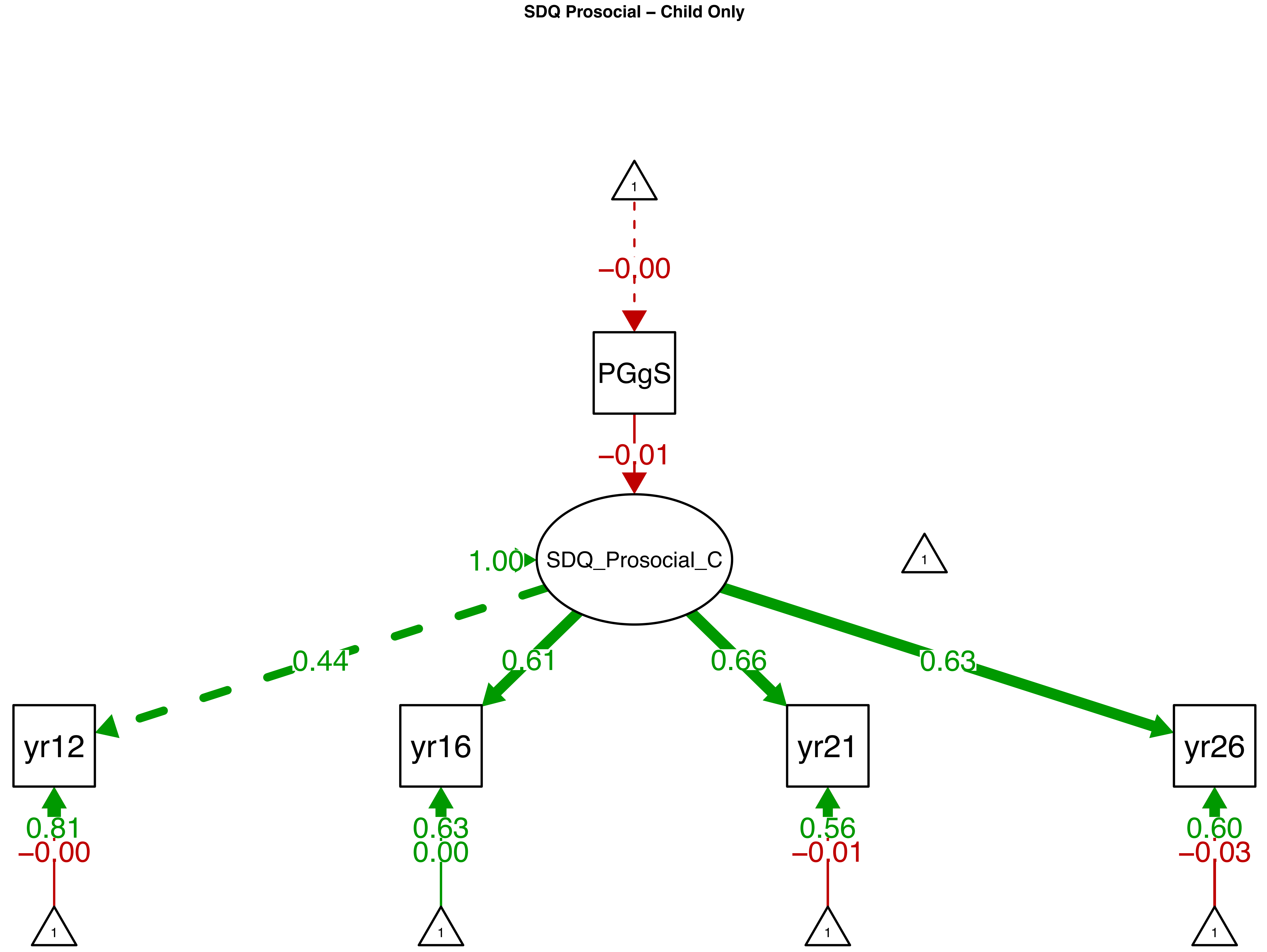
SDQ Prosocial – Child Only
1
–0.00
PGgS
–0.01
1.00
SDQ_Prosocial_C
1
0.44
0.61
0.66
0.63
yr12
yr16
yr21
yr26
0.81
–0.00
0.63
0.00
0.56
–0.01
0.60
–0.03
1
1
1
1

C10-31

SDQ Total Problems – Parent Only

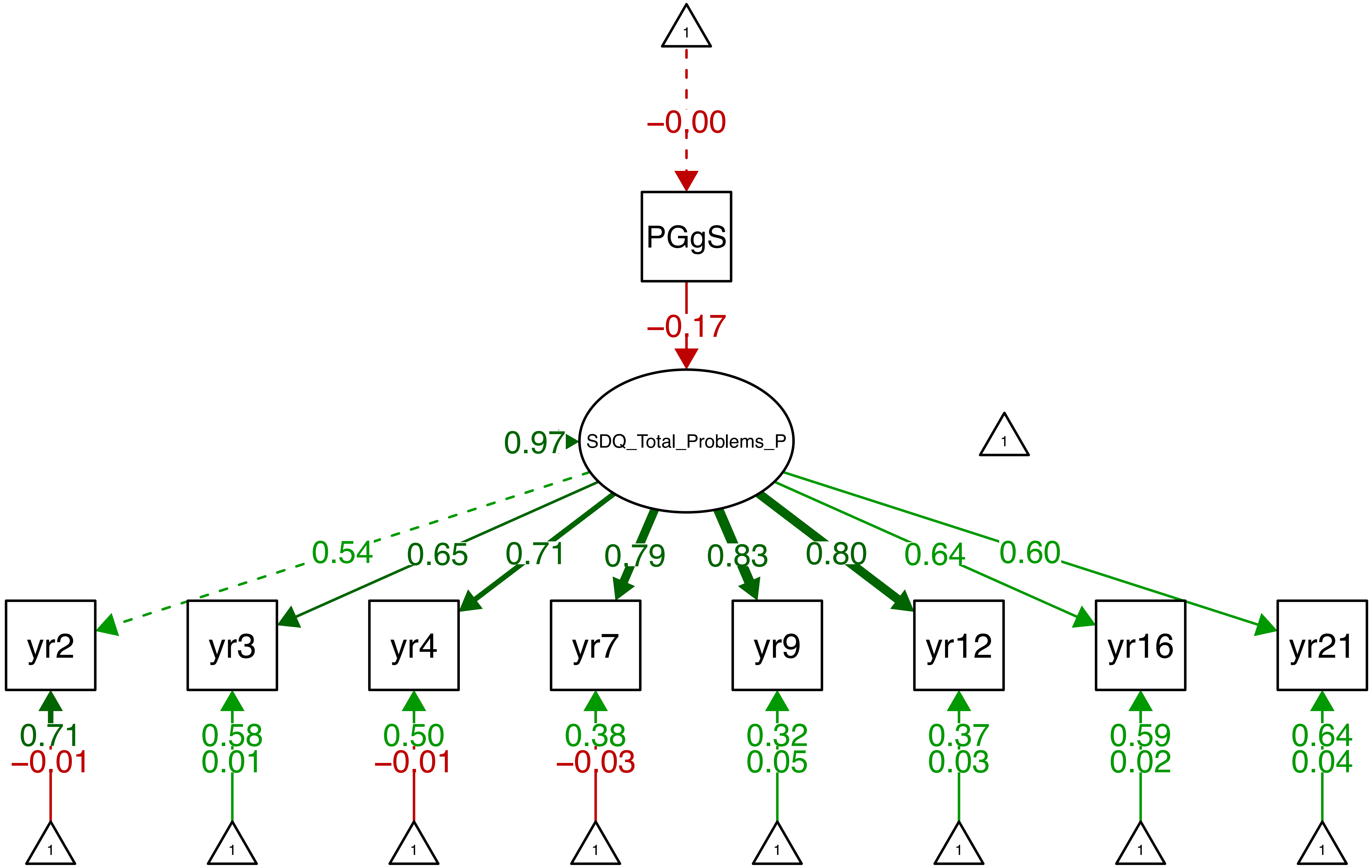

C10-32

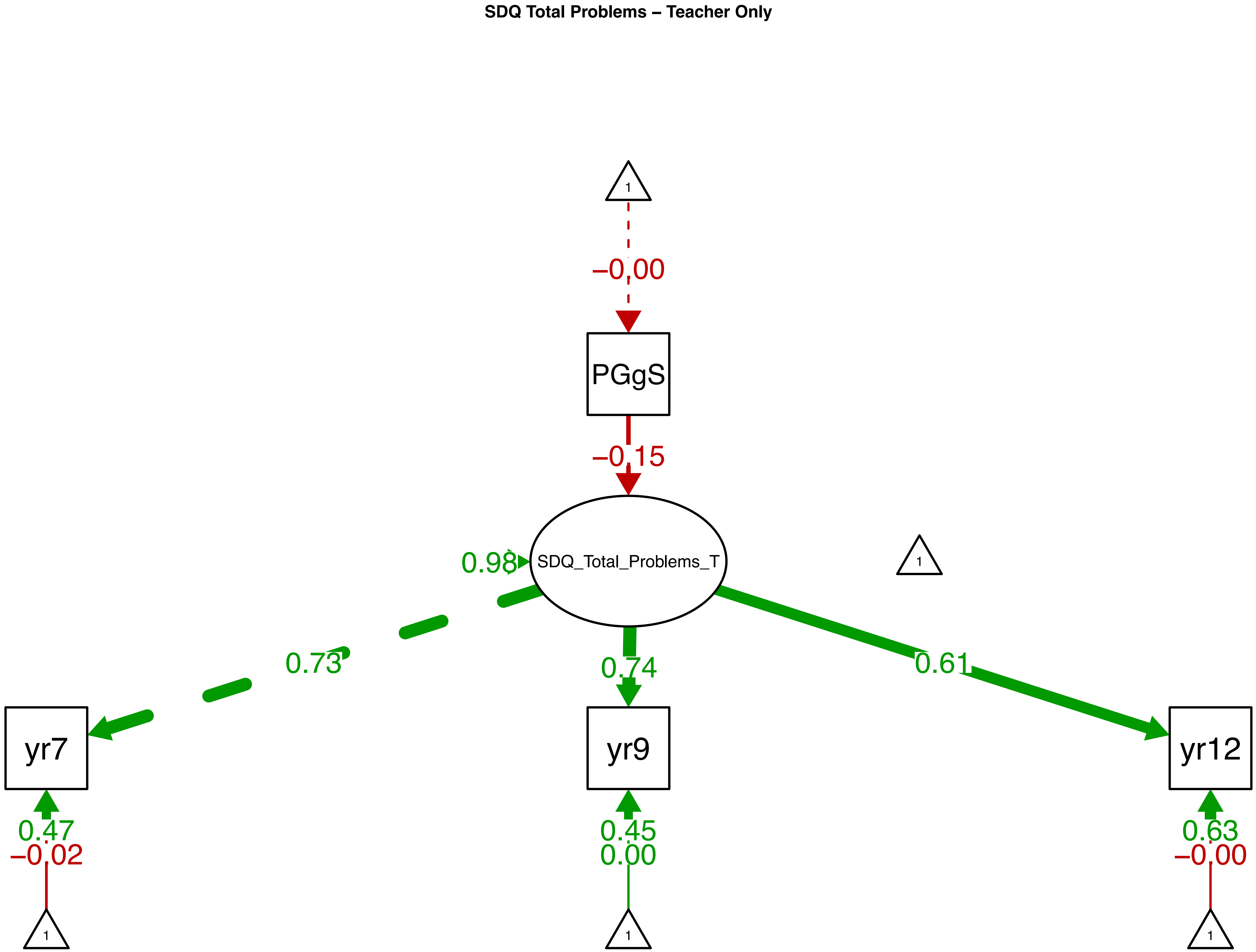
SDQ Total Problems – Teacher Only
1
−0.00
PGgS
−0.15
0.98
SDQ_Total_Problems_T
1
0.73
0.74
0.61
yr7
yr9
yr12
0.47
−0.02
0.45
0.00
0.63
−0.00
1
1
1

SDQ Total Problems – Child Only

C10-33

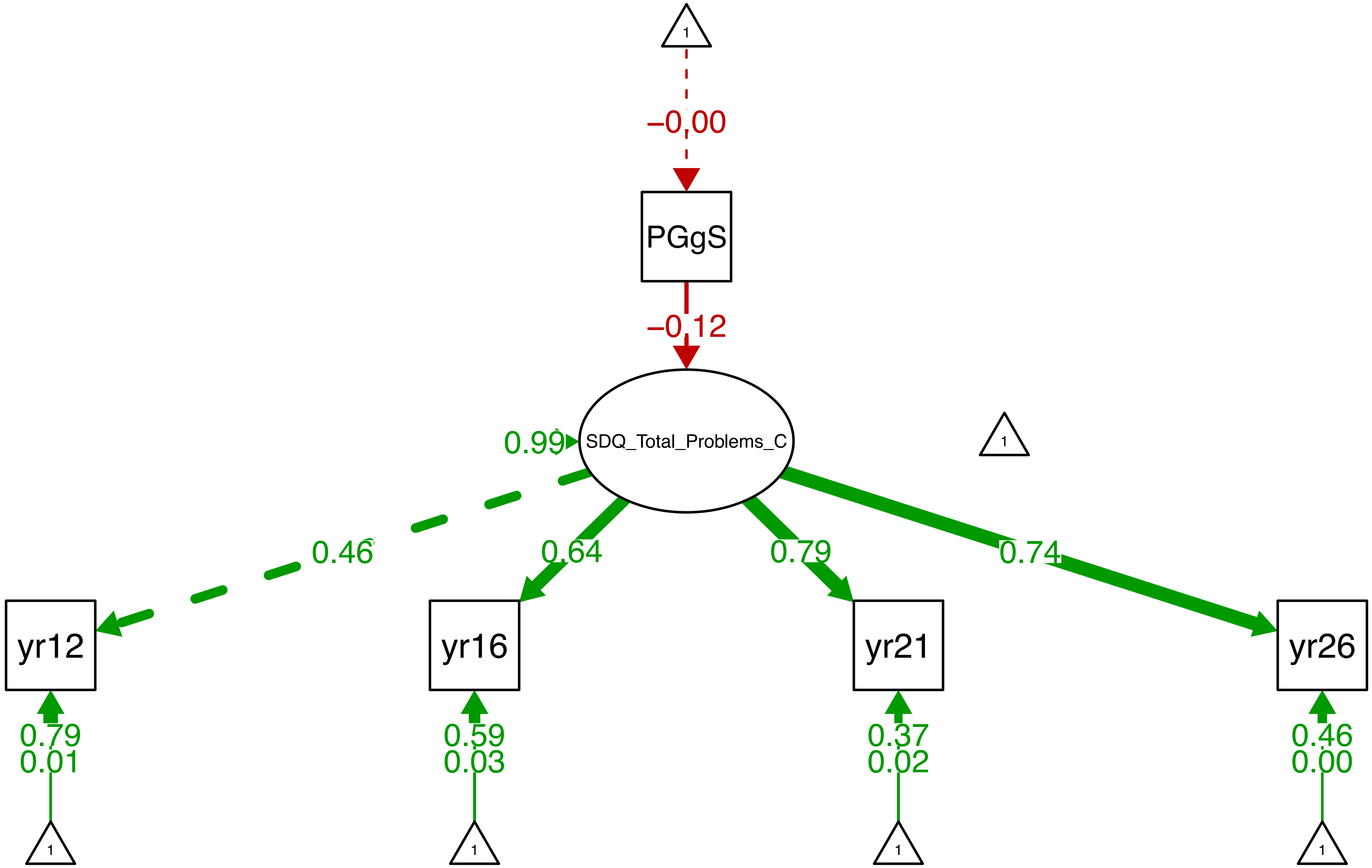

C10-34 SDQ Conduct Problem - Cross Rater

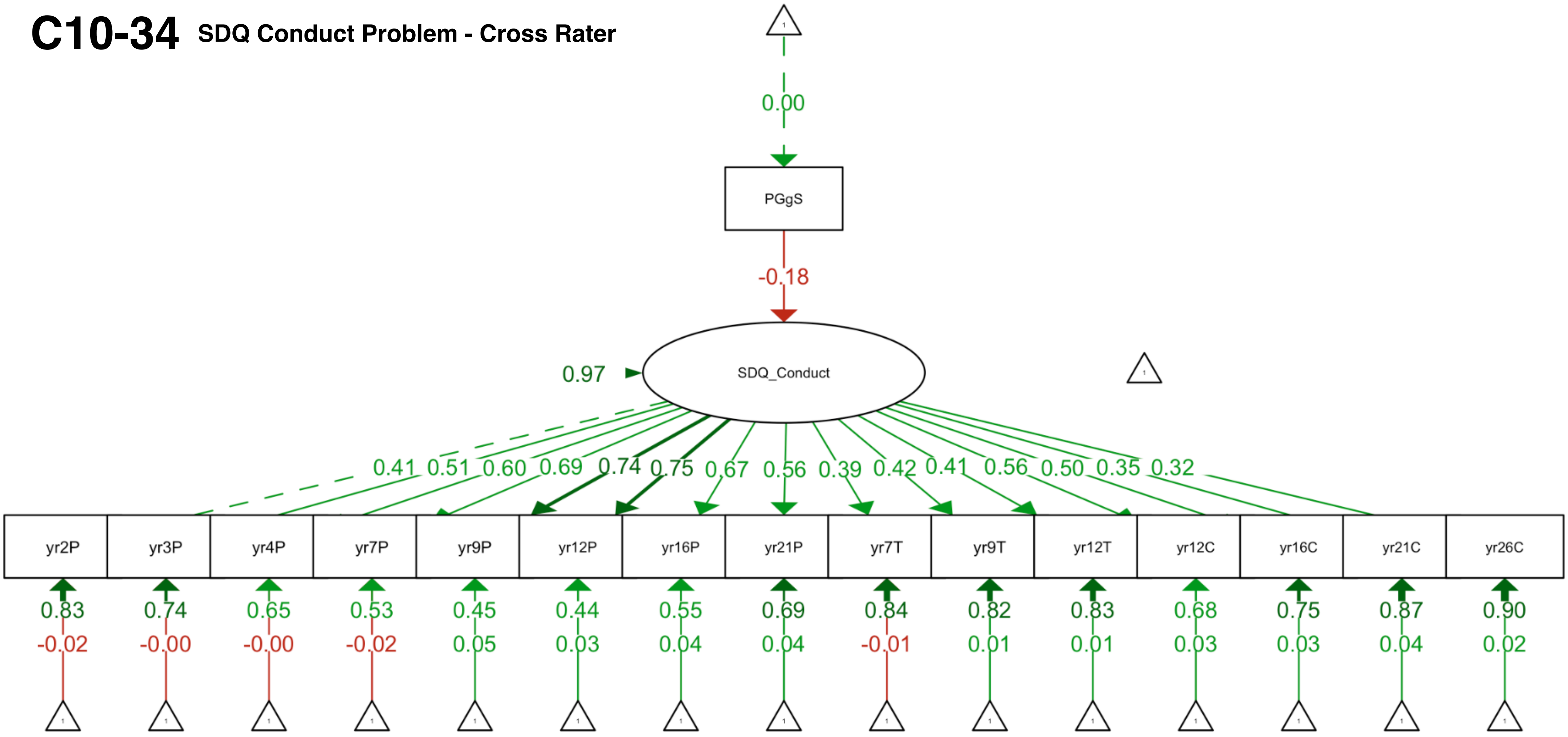

C10-35 SDQ Emotional Problem - Cross Rater

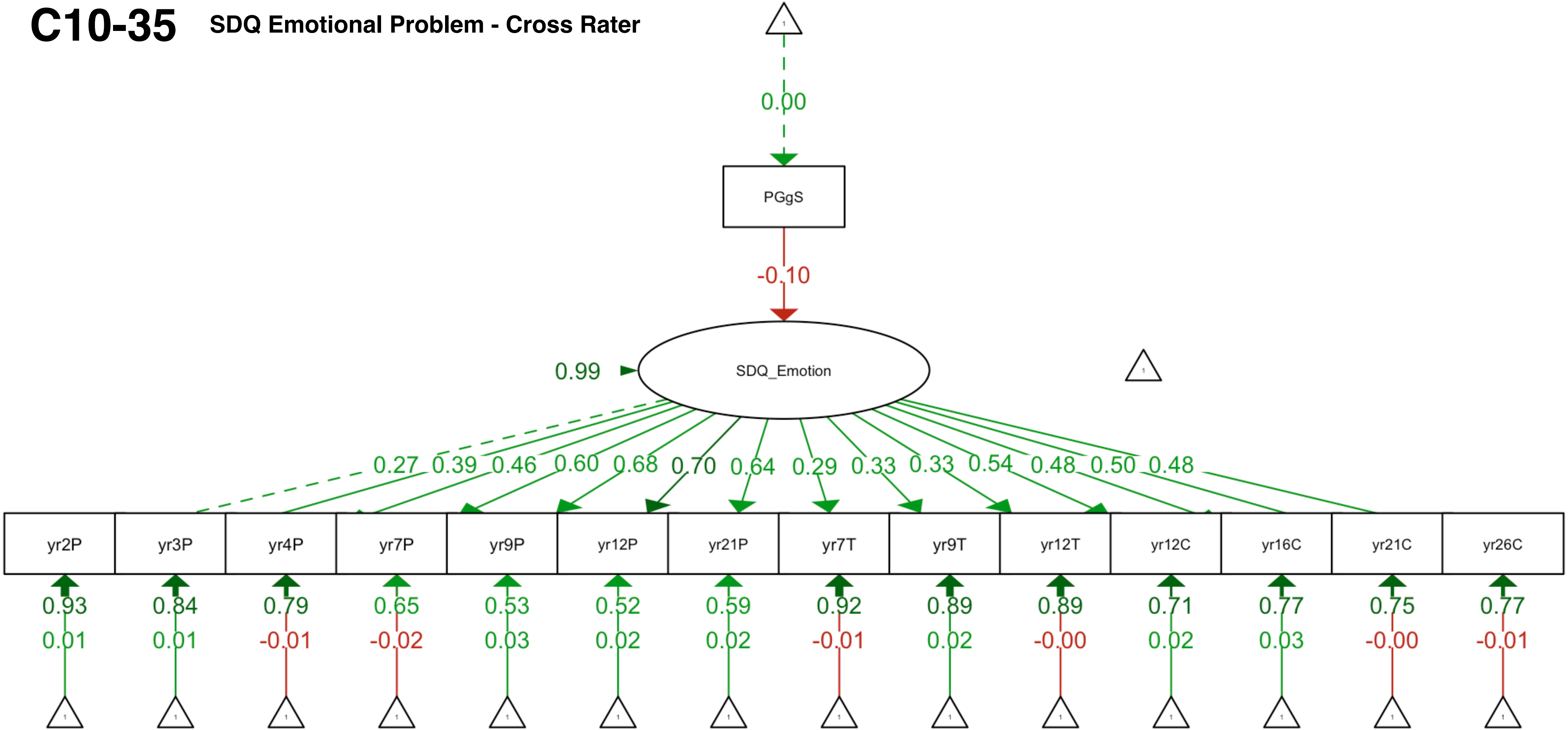

C10-36 SDQ Hyperactivity - Cross Rater

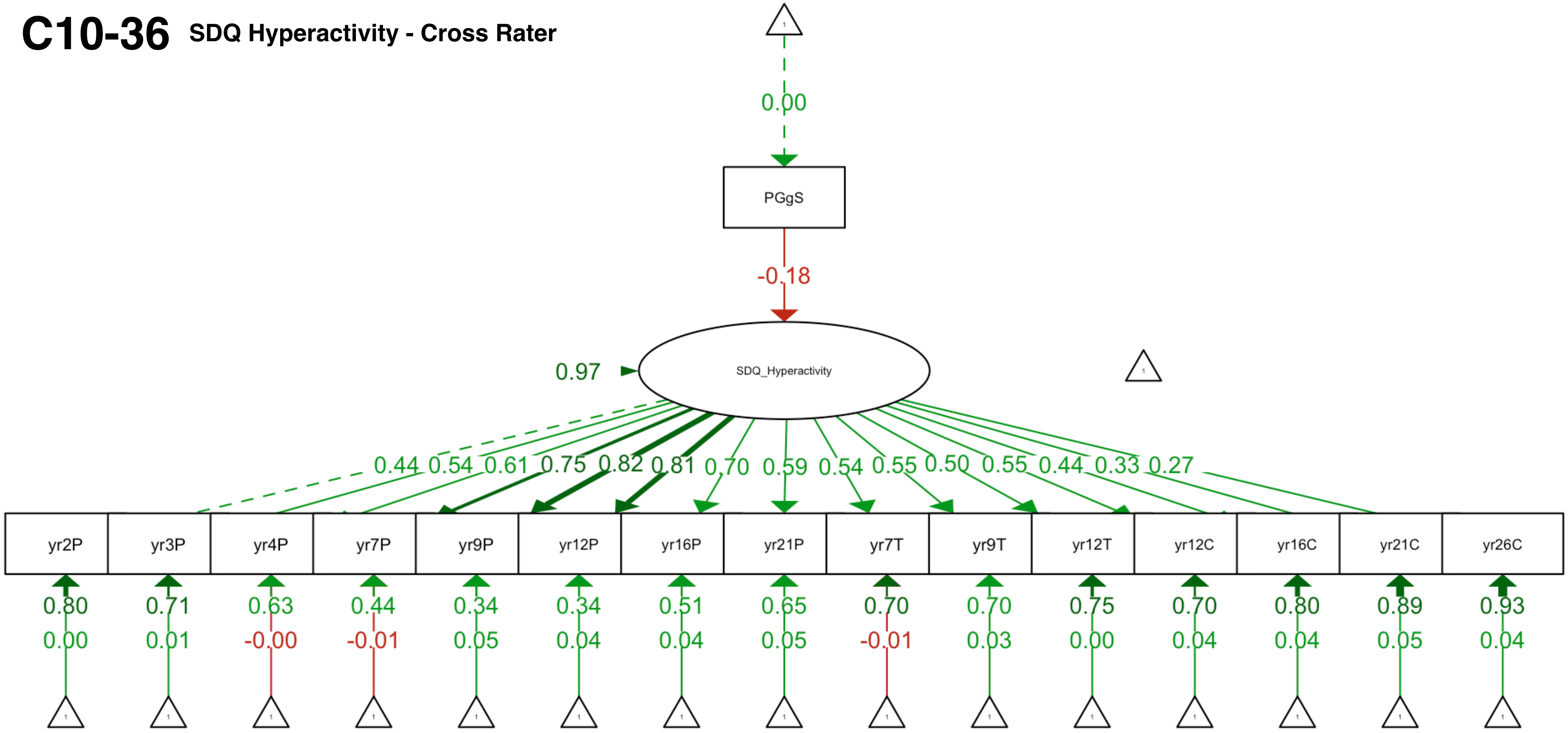

C10-37 SDQ Peer Problem - Cross Rater

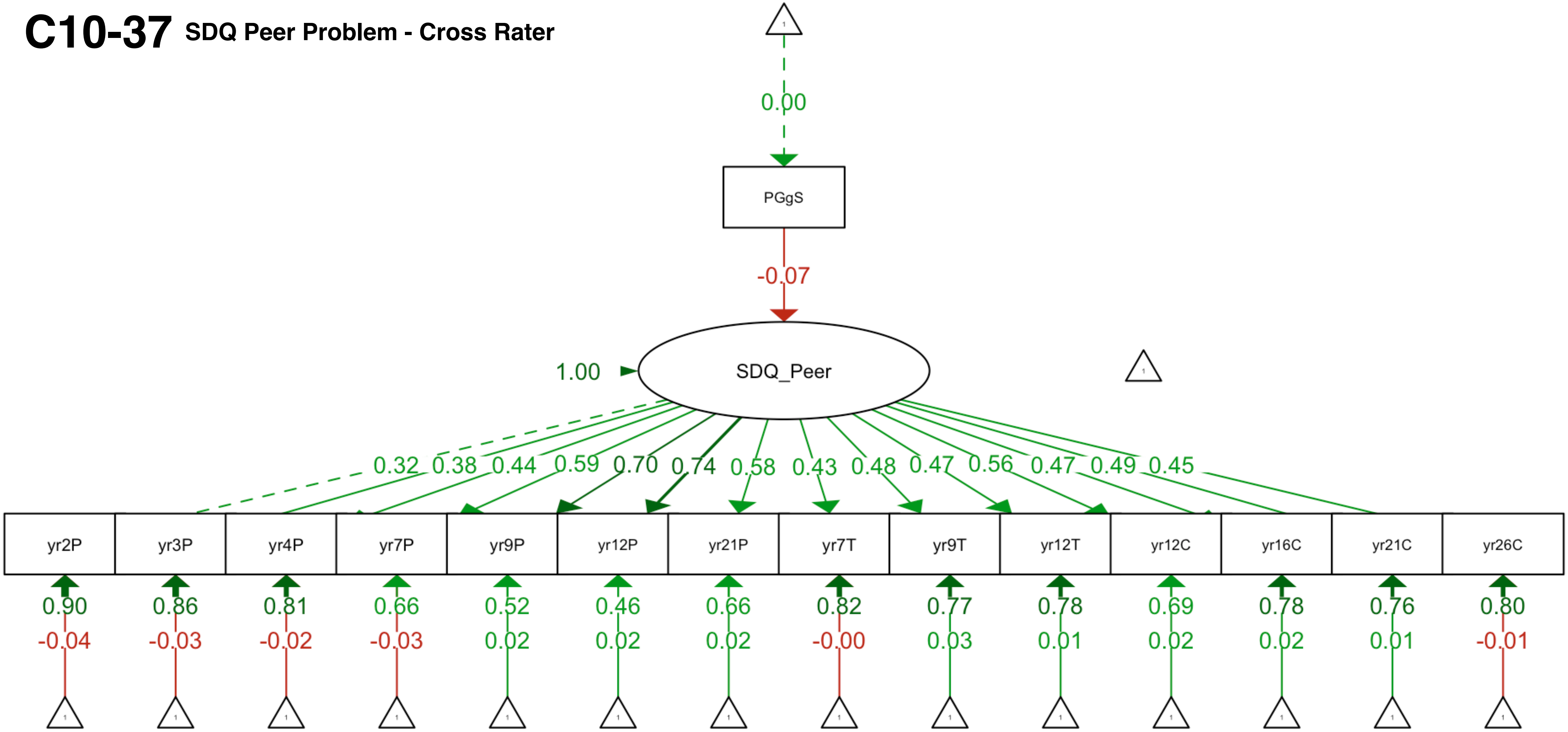

C10-38 SDQ Prosocial - Cross Rater

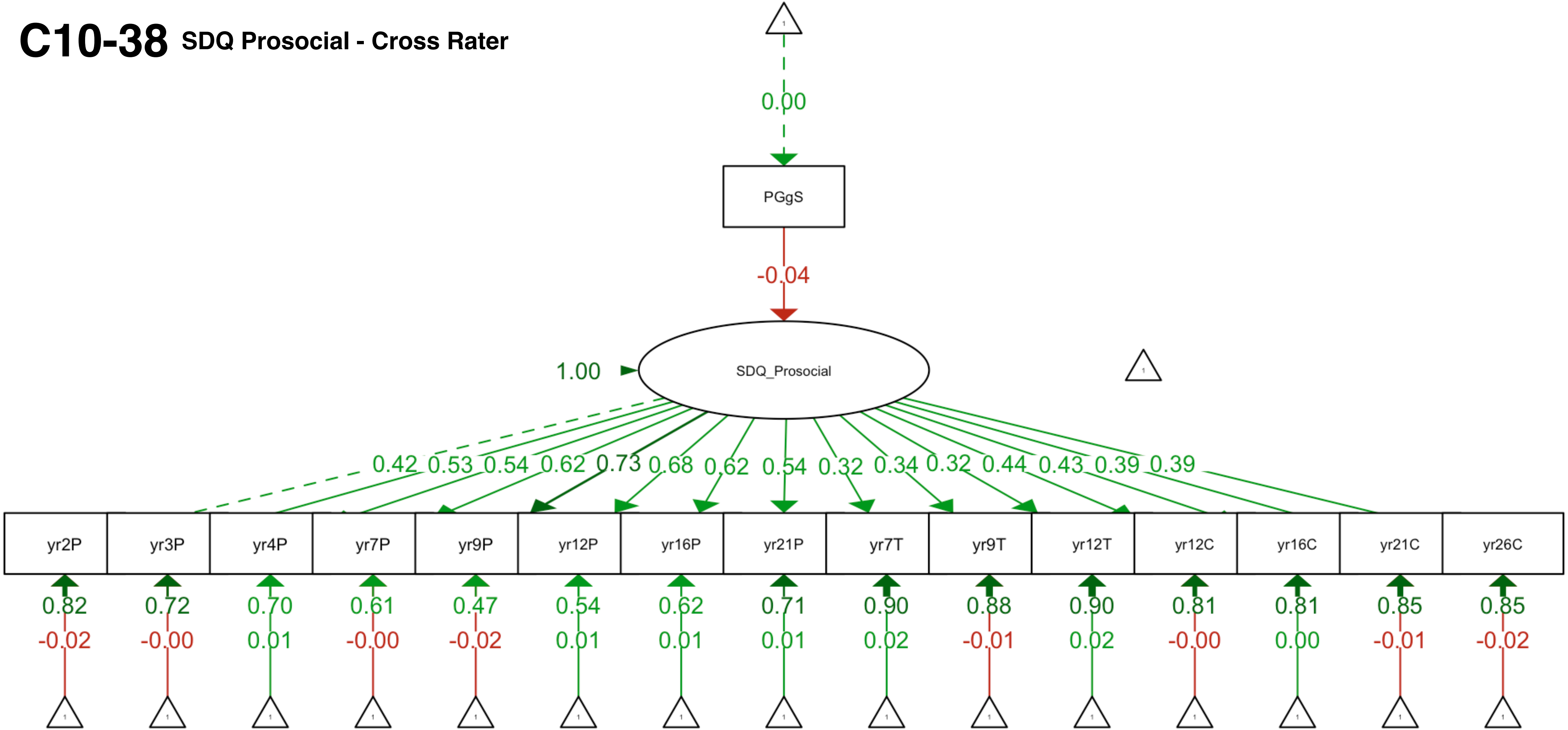

C10-39 SDQ Total Problems - Cross Rater

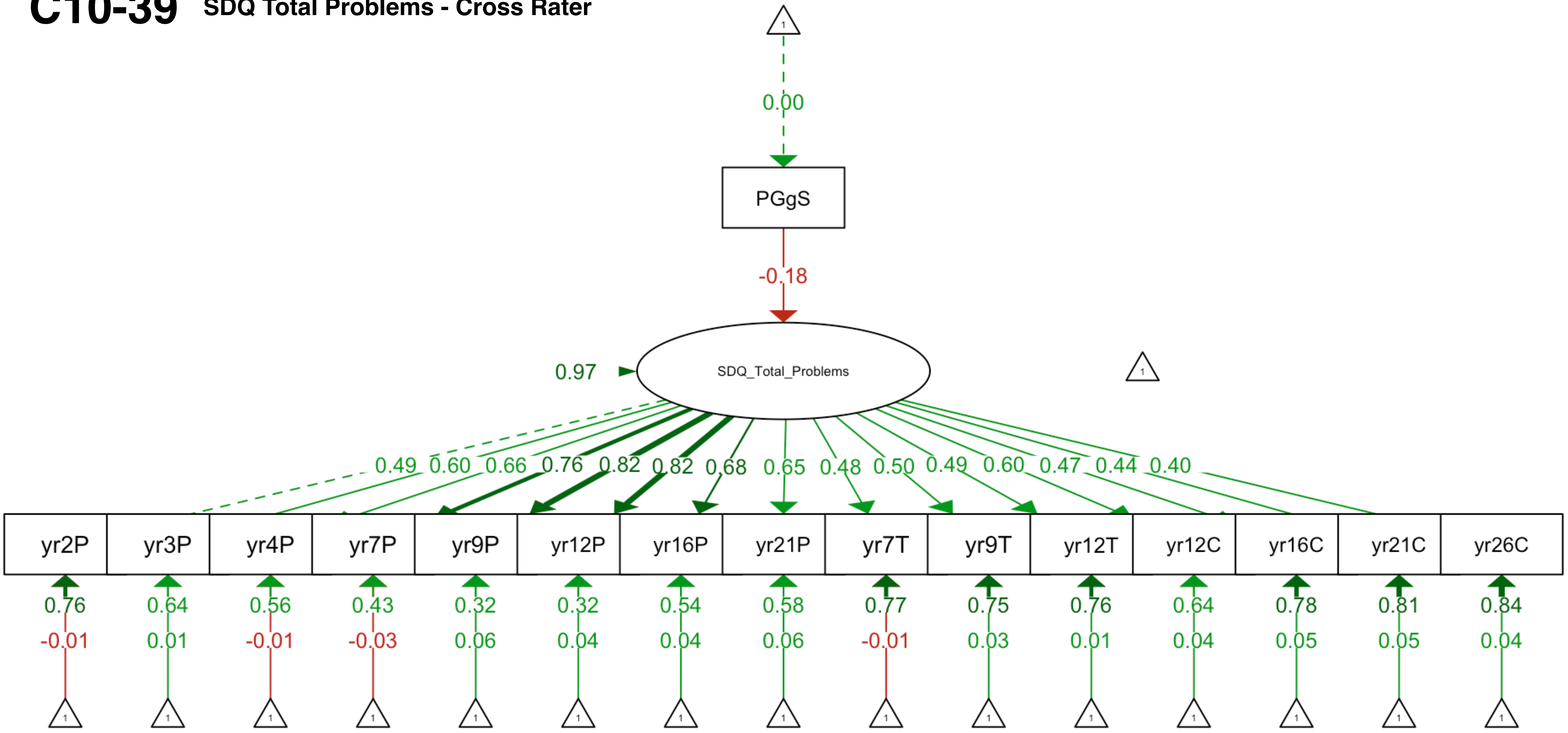

Supplement: Supplement 4 [file media-4.pdf]
